# Use of selective gut decontamination in critically ill children: protocol for the Paediatric Intensive Care and Infection Control (PICnIC) pilot study

**DOI:** 10.1136/bmjopen-2022-061838

**Published:** 2022-03-11

**Authors:** Alanna Brown, Paloma Ferrando, Mariana Popa, Gema Milla de la Fuente, John Pappachan, Brian Cuthbertson, Laura Drikite, Richard Feltbower, Theodore Gouliouris, Isobel Sale, Robert Shulman, Lyvonne N Tume, John Myburgh, Kerry Woolfall, David A Harrison, Paul R Mouncey, Kathryn M Rowan, Nazima Pathan

**Affiliations:** 1 Intensive Care National Audit and Research Centre, London, UK; 2 Institute of Life and Human Sciences, University of Liverpool, Liverpool, UK; 3 PICU, Southampton Children's Hospital, Southampton, UK; 4 Department of Critical Care, Sunnybrook Health Sciences Centre, Toronto, Ontario, Canada; 5 Clinical Trials Unit, Intensive Care National Audit and Research Centre, London, UK; 6 University of Leeds, Leeds, UK; 7 Department of Medicine, Cambridge University, Cambridge, UK; 8 PPI representative, UK, UK; 9 Department of Pharmacy, University College London Hospitals NHS Foundation Trust, London, UK; 10 School of Health and Society, University of Salford, Salford, UK; 11 The George Institute for Global Health, Newtown, New South Wales, Australia; 12 University of Liverpool, Liverpool, UK; 13 Department of Paediatrics, University of Cambridge, Cambridge, UK; 14 Cambridge University Hospitals NHS Foundation Trust, Cambridge, UK

**Keywords:** infection control, paediatric intensive & critical care, microbiology

## Abstract

**Introduction:**

Healthcare-associated infections (HCAIs) are a major cause of morbidity and mortality in critically ill children. In critically ill adults, there are data that suggest the use of Selective Decontamination of the Digestive tract (SDD), alongside standard infection control measures reduce mortality and the incidence of HCAIs. SDD-enhanced infection control has not been compared directly with standard infection prevention strategies in the Paediatric Intensive Care Unit (PICU) population. The aim of this pilot study is to determine the feasibility of conducting a multicentre cluster randomised controlled trial (cRCT) in critically ill children comparing SDD with standard infection control.

**Methods and analysis:**

Paediatric Intensive Care and Infection Control is a parallel group pilot cRCT, with integrated mixed-methods study, comparing incorporation of SDD into infection control procedures to standard care. After a 1-week pretrial ecology surveillance period, recruitment to the cRCT will run for a period of 18 weeks, comprising: (1) baseline control period (2) pre, mid and post-trial ecology surveillance periods and (3) intervention period. Six PICUs (in England, UK) will begin with usual care in period 1, then will be randomised 1:1 by the trial statistician using computer-based randomisation, to either continue to deliver usual care or commence delivery of the intervention (SDD) in period 2. Outcomes measures include parent and healthcare professionals’ views on trial feasibility, adherence to the SDD intervention, estimation of recruitment rate and understanding of potential patient-centred primary and secondary outcome measures for the definitive trial. The planned recruitment for the cRCT is 324 participants.

**Ethics and dissemination:**

The trial received favourable ethical opinion from West Midlands—Black Country Research Ethics Committee (reference: 20/WM/0061) and approval from the Health Research Authority (IRAS number: 239324). Informed consent is not required for SDD intervention or anonymised data collection but is sought for investigations as part of the study, any identifiable data collected and monitoring of medical records. Results will be disseminated via publications in peer-reviewed medical journals.

**Trial registration number:**

ISRCTN40310490.

**Strengths and limitations of this study**

- The study will examine the processes that are important in a future clinical trial of Selective Decontamination of the Digestive tract (SDD) in the paediatric intensive care unit (PICU) setting.
- The study uses a GMP-certified commercial SDD preparation under licence from the George Institute, Australia (Verita Pharma Pty, Australia).
- The study will evaluate the perspective of parents and the views of stakeholders including caregivers and the multi-disciplinary team on the processes needed to undertake a trial of SDD in the PICU.
- The use of SDD-enhanced infection control requires it to be implemented unit-wide, along with the support of local microbiology, pharmacy and infection control teams for delivery and monitoring in sites randomised to implement it.
- This is a pilot study, so is not designed to assess effectiveness of the intervention; the study outcomes will inform the feasibility of a future trial based on clinical outcomes.

## Introduction

In critically ill children, healthcare-associated infections (HCAIs) are a major cause of morbidity and mortality, with a reported prevalence of 7%–14%.^1–5^ HCAIs can develop either as a direct result of healthcare interventions such as medical or surgical treatment, or from being in contact with a healthcare setting. In the critical care setting, the high use of invasive devices such as endotracheal tubes, vascular and urinary catheters increase the risk of secondary infection by opportunistic organisms. In particular, respiratory HCAIs (ventilator associated pneumonia, VAP) may occur with spread of commensal and other organisms from oro-pharyngeal and upper gastrointestinal compartments into the lungs.^4 5^

Evidence from adult intensive care studies suggests that using Selective Decontamination of the Digestive tract (SDD) alongside standard infection control measures reduces mortality and VAP.^6 7^ It has been shown that the use of SDD influences the microbiological ecology of the unit, thereby reducing incidence of HCAIs in both exposed and non-exposed patients. Despite this, SDD has not been routinely adopted due to concerns that it may promote antimicrobial resistance.^6 8^ Recent ecological studies conducted in adult intensive care have found that SDD was associated with a reduction in antibiotic utilisation^9–13^; two large cluster randomised controlled trials (cRCTs) have been recently undertaken to further evaluate the clinical effects of SDD in adult intensive care. One reported no change in the incidence of blood stream infections, but the incidence of ventilator acquired pneumonia and overall antimicrobial use have not yet been reported.^14^ Another large-scale multicentre study using the same formulation as the Paediatric Intensive Care and Infection Control (PICnIC) study has recently completed enrolment of critically ill adults in Australia, Canada and the UK.^15^

SDD has yet to be compared directly with modern infection control protocols within the paediatric intensive care unit (PICU) population. The only trial data suggest a reduction in incidence of VAP but not mortality, however the study was underpowered and the observed mortality was very low.^16^ Therefore, a clinical trial comparing SDD with standard infection control methods is required. Given the paucity of data describing the use of SDD in PICU and to establish the appropriate safety and ecological monitoring protocols, it is first imperative to establish whether a large, multicentre trial is feasible.

The PICnIC pilot study is a feasibility study designed to determine whether it is possible to conduct a cRCT of SDD in critically ill children who are likely to be ventilated for >48 hours, and to explore and test the acceptability of key components of the study to healthcare professionals and families of patients.

## Methods and analysis

### Aim

To determine whether it is feasible to conduct a multicentre trial in critically ill children comparing SDD with standard infection control procedures.

### Objectives

- To test the ability to randomise PICUs to either control or intervention.
- To test the willingness and ability of healthcare professionals to screen and recruit eligible children.
- To estimate the recruitment rate of eligible children.
- To test adherence to the SDD protocol (including tolerance and application of a standardised SDD paste and suspension in a paediatric population).
- To test the procedures for assessing and collecting selected clinical and ecological outcomes and for adverse event (AE) reporting.
- To assess the generalisability of the study.
- To explore parent and healthcare professional views on the acceptability of the proposed trial, including recruitment and consent procedures and patient centred outcomes

### Study setting

Six PICUs based in England, UK with a diverse geographical/demographic population representative of national (UK) PICU activity and size.

### Design

**Figure 1:**
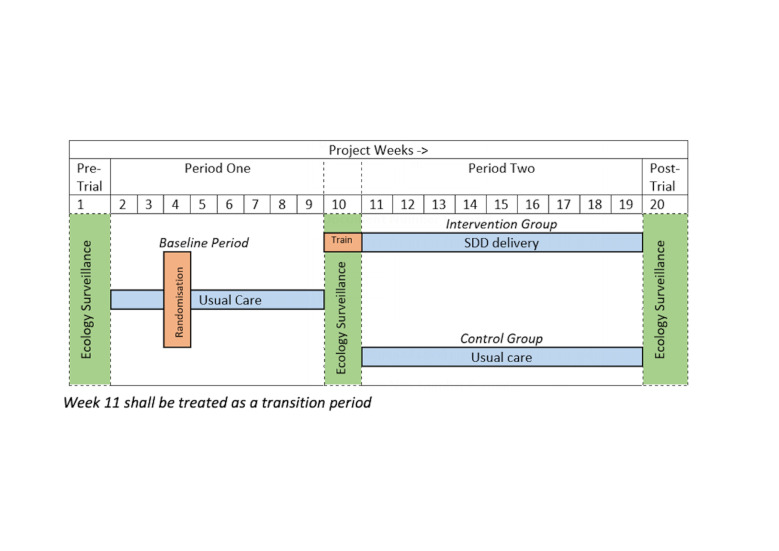
Trial design. SDD, Selective Decontamination of the Digestive.

**Figure 2:**
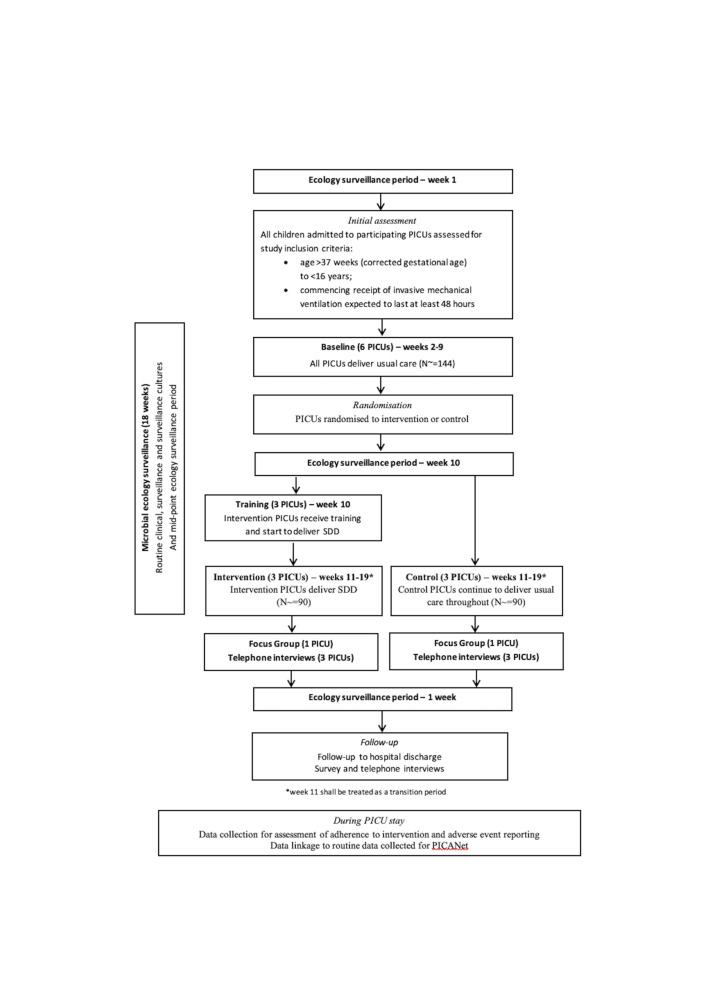
Trial schema of eligibility, site randomisation, study intervention, ecology surveillance, embedded mixed-methods study, follow-up. PICANet, Paediatric Intensive Care Audit Network; PICU, paediatric intensive care unit; SDD, Selective Decontamination of the Digestive.

External pilot, parallel group cRCT with integrated mixed-methods study. After a 1-week pretrial ecology surveillance period, recruitment to the cRCT will run for a period of 18 weeks, comprising: (1) baseline control period (‘period 1’—weeks 2–9); (2) mid-trial ecology surveillance period (week 10) and (3) intervention period (‘Period 2’—weeks 11–19). On completion of period 2, an additional 1-week post-trial ecology surveillance period will be carried out (figure 1). Sites will be randomised 1:1 by the trial statistician using computer-based randomisation, to either continue to deliver usual care or commence delivery of the intervention (SDD) in period 2 (figure 2).

### Screening

Potentially eligible patients presenting to the participating unit will be screened against the inclusion/exclusion criteria by the local clinical team, supported by the site research team. Screening Logs will record the reason patients are eligible but are subsequently not enrolled.

### Eligibility: ecology surveillance periods

#### Inclusion criteria

- All patients admitted to the PICU, regardless of ventilation status, during any of the three ecological surveillance periods.

#### Exclusion criteria

- None.

### Eligibility: period 1 and period 2

#### Inclusion criteria

- >37 weeks corrected gestational age to <16 years.
- Receiving invasive mechanical ventilation, expected to last at least 48 hours.
- Expected to remain on invasive mechanical ventilation until the day after tomorrow (from time of screening).

#### Exclusion criteria

- Known allergy, sensitivity or interaction to polymyxin E (colistin), tobramycin or nystatin.
- Known to be pregnant.
- Death perceived as imminent.

### Microbiology sampling

#### Samples taken

- Nasopharyngeal.
- Stool/rectal swabs.
- Urine (if clinically indicated).
- Sputum/secretions from the endotracheal tube (If clinically indicated).
- Wound swabs, if present (if clinically indicated).

During the designated ecology surveillance periods, samples will be taken on admission and then taken on a Friday, if the patient has not had samples taken in the previous 48 hours.

During periods 1 and 2, samples will be taken on admission and then twice weekly until discharge. For patient stays of less than 7 days, samples should be taken on the day of discharge.

### Trial intervention

During the Ecology Surveillance periods, period 1 and for sites randomised to usual care in period 2, there is no intervention. Patients will receive all standard infection control measures (per the site’s specific policies) but will receive no study specific intervention.

For sites randomised to the intervention in period 2, it will form part of the standard infection control strategy in the participating PICU. In addition to usual care, an SDD-enhanced infection control regimen will be delivered to all eligible patients using a Good Manufacturing Practice (GMP)-certified commercial SDD preparation under licence from The George Institute, Australia (Verita Pharma Pty, Australia). (Within online supplemental file 1, online supplemental table 1) for SDD administration details, (online supplemental tables 2 and 3) for composition and characteristics of SDD, (online supplemental tables 4 and 5) for SDD formulation, online supplemental appendix 1 for GMP licence, online supplemental appendices 2–8 for SDD stability data and online supplemental appendix 9 for PICnIC labels). Dosing of the SDD suspension will be calculated according to age (table 1):

10.1136/bmjopen-2022-061838.supp1 Supplementary data

**Table 1:** SDD suspension dosing

	0–4 years	5–12 years	≥13 years
Polymyxin E (Colistin)	25 mg	50 mg	100 mg
Tobramycin	20 mg	40 mg	80 mg
Nystatin	0.5×106 IU	1×106 IU	2×106 IU
	2.**5** mL	**5** mL	10 mL

SDD, Selective Decontamination of the Digestive.

1. A 6 hourly topical, application of a pea-sized (0.5 g) SDD paste containing 2% polymyxin E (colistin), 2% tobramycin and 2% nystatin to the buccal mucosa and oropharynx.
2. A 6 hourly administration of SDD suspension administered via the most proximal feeding tube into the stomach containing polymyxin E (colistin), tobramycin and nystatin.

SSD preparations will be distributed by the manufacturer in temperature-controlled (2°C–8°C), patient specific kits to the participating hospital pharmacies. Each kit contains a 5-day supply of SDD treatment which includes a bottle of SDD suspension powder for reconstitution and 20×1 mL syringes containing Oral Paste. When a kit is allocated to a patient and the SDD powder is reconstituted into suspension, the kit can be stored at room temperature (≤27°C) for up to 5 days. SDD treatment should be started within 6 hours of the patient being identified as eligible and continue for a maximum of 30 days (treatment period).

Treatment will continue until the patient is extubated or no longer mechanically ventilated (in tracheostomised patients). Patients subsequently reintubated (either during this PICU admission or readmission to PICU from another inpatient area) during the treatment period will restart the intervention. All other usual care will be provided at the discretion of the treating clinical team.

### Consent procedures

Children who are eligible for PICnIC will often become so during a period of life-threatening illness. This is a stressful time for parents/guardians during which time there are ethical concerns both about the burden placed of trying to understand the trial and their ability to provide informed consent.^17^

Posters displayed throughout the PICU will explain ecology sampling procedures and invite parents/guardians to have further discussions with research staff. The SDD intervention will be delivered, in additional to standard infection control policies, to all eligible patients in sites randomised to the SDD intervention during period 2. Research staff will not seek individual consent for this. This approach is in line with guidance from the Ottawa Statement on the ethical design and conduct of cRCT.^18 19^ (See online supplemental file 2) for poster and consent forms).

10.1136/bmjopen-2022-061838.supp2 Supplementary data

Consent will be required for:

- Any additional study-specific samples to be taken, stored and analysed prior to being taken solely for the study and not as part of routine care.
- Identifiable data collected and processed for participation in the mixed methods aspects of the study (questionnaire and interview).
- Monitoring of medical records.

Consent will not be required for:

- Samples taken as part of routine care (eg, admission samples).
- Anonymised data collection and processing from routine sources.
- Delivery of SDD. For PICUs randomised to the intervention during period 2, all children meeting the eligibility criteria will receive SDD as the standard practice.

Children may be withdrawn from trial-specific data collection by the request of parents who decline participation in the research. All data collected up to the point of withdrawal will be retained and included in the study analysis.

If the patient has died, the parents/guardians will be approached for consent for the monitoring of medical records and identifiable data collected, and for the interview aspect of the study once established whether it is appropriate to approach for consent.

### Data collection

Detailed guidance for the collection of data will be provided in the trial-specific standard operating procedure. It will include:

- Demographics.
- Date/time of commencing mechanical ventilation.
- Date/time identified as eligible.
- Antibiotic usage throughout admission (route, type, duration, frequency, dose).
- Date/time of final extubation.
- Details of any HCAI (confirmed/presumed).
- Date/time of PICU discharge.
- Date/time of hospital discharge.
- SDD delivery (dose for age, date time first dose, dose per day, change of dose, protocol deviation and reason for deviation)

No identifiable participant data will be required by the ICNARC Clinical Trials Unit (CTU) and all participant data will be stored securely. ICNARC is registered under the Data Protection Act (1998), and all ICNARC CTU staff have undergone data protection and International Conference on Harmonisation (ICH) Good Clinical Practice (GCP) training.

Patients will be followed up until discharge from a participating PICU. Data entered onto the secure trial database will undergo validation checks for completeness, accuracy and consistency of data. Queries on incomplete, inconsistent, or non-adherent data will be sent to the relevant PICU team for resolution.

Routine linkage will be made for all patients with the Paediatric Intensive Care Audit Network (PICANet) through the PICANet ID and trial number to obtain:

- Baseline demographics and risk factors, including predicted risk of death.
- Secondary outcomes of critical care and acute hospital mortality, organ support received

### Safety monitoring

AE reporting will follow the Health Research Authority (HRA) guidelines on safety reporting in studies which do not use Investigational Medicinal Products.

AEs will be recorded during period 2, from enrolment until PICU discharge (including readmissions from other inpatient areas until 30 days from being identified as eligible). For patients receiving SDD intervention, only AEs deemed possibly, probably or definitely related to the trial intervention should be reported to ICNARC CTU, apart from NG tube blockages which should be reported even if not deemed related. For patients receiving usual care, only NG tube blockages will be reported.

The following events have been prespecified as potential AEs:

- NG tube blockage.
- Choking on paste
- Allergic reaction to SDD.

The following events are exempt for reporting as AEs or serious AEs (SAEs)

- Deterioration of condition or death that is not related to the trial intervention.
- AEs of other drugs not specified in the protocol.

Any event classified as ‘severe’, ‘life-threatening’ or ‘fatal’ in severity is considered an SAE and must be reported to ICNARC CTU. If the SAE is evaluated by the chief investigator or clinical member of the Trial Management Group (TMG) as a related and unexpected SAE, the ICNARC CTU will submit a report to the Research Ethics Committee (REC) within 15 calendar days.

### Patient and public involvement

Caregivers of children admitted to PICU and a former patient were involved in prioritising the outcomes and designing the study protocol. Their input has continued with patient and public involvement (PPI) representatives on the study oversight panel. A patient representative (former PICU patient) is a coinvestigator and is an author of this manuscript.

### Outcome measures

As this is a pilot study, outcome measures for the study will be focused on assessing the feasibility of a larger scale definitive study.

The ability to randomise PICUs to either control or intervention will be assessed by the successful random assignment of three PICUs to the intervention without delay to subsequent phases of the trial.

The willingness and ability of healthcare professionals to screen and recruit eligible children will be assessed by the proportion of eligible children recorded on study screening logs successfully recruited to the pilot cRCT and the reported reasons for non-recruitment.

The potential recruitment rate for a future definitive cRCT trial of SDD-enhanced infection control in eligible children will be estimated by combining the proportion of eligible children recruited to the pilot cRCT with the size of the potentially eligible population (estimated from nesting the screening log data from participating PICUs within the national UK PICU data from PICANet).

Adherence to the SDD protocol will be assessed by the proportion of eligible children allocated to the intervention receiving (1) both elements and (2) each individual element of the SDD intervention, the number of days on which these elements were received relative to days eligible for the SDD intervention and the reported reasons for nonadherence.

Procedures for assessing and collecting selected clinical and ecological outcomes and for AE reporting will be assessed by the proportion of children with complete data for these outcomes including, for ecological outcomes, the proportion consenting to additional study specific sample collection.

Generalisability of the study results to all UK PICUs will be assessed by comparing baseline characteristics and outcomes for children recruited to the pilot cRCT with data from all potentially eligible children (receiving invasive mechanical ventilation for at least three calendar days) within participating PICUs and within all UK PICUs (from PICANet).

With the aim of understanding potential patient-centred primary and secondary outcome measures for the definitive cRCT, the following potential outcome measures will be reported:

- HCAI (confirmed/presumed) and microbiology results (if positive sample).
- Hospital mortality.
- PICU mortality.
- Mortality within 30 days postenrolment.
- Length of hospital stay.
- Length of stay in PICU.
- Duration of mechanical ventilation.
- Organ support received.

### Statistical methods

#### Sample size

The PICnIC pilot study is set up to test the feasibility of the protocol to recruit eligible patients. Therefore, there is no primary outcome to be compared between the two groups and, hence, a usual power calculation to determine sample size is not appropriate. Instead, the sample size has been determined to be adequate to estimate critical parameters to be tested to a necessary degree of precision. Based on available data from PICANet, it is anticipated participating sites will see approximately 4.5 eligible children per week, therefore, the anticipated recruitment rate is three children per PICU per week providing a total of approximately 324 children in 18 weeks, of which 90 would receive the intervention. The sample size of children receiving usual care would be sufficient to estimate a binary outcome present in 20% of the population with a precision of ±5%.

#### Analysis

An overview of the planned analyses for the PICnIC pilot study is provided below. The full statistical analysis plan will be lodged on the trial website ahead of database lock.

A Consolidated Standards of Reporting Trials flow diagram will be used to summarise the number and percentage of children screened, recruited and followed up. This will include the proportion of eligible children successfully recruited and the reported reasons for non-recruitment.

Recruitment to the pilot cRCT will be presented as a rate per site per week over the two recruitment periods, overall, by treatment group and by site. Potential reasons for variation in recruitment rates will be explored. The potential recruitment rate for a future definitive cRCT will be estimated by combining the proportion of eligible children recruited to the pilot cRCT with the size of the potentially eligible population (estimated from PICANet).

Baseline demographic and clinical data will be summarised overall and for each of the two treatment groups in each of the two time periods but not subjected to statistical testing.

The proportion of eligible children allocated to the intervention that received both elements and each individual element of the SDD intervention will be reported as well as the number of days on which these elements were received relative to days eligible for the SDD intervention. The reported reasons for nonadherence will be detailed.

Data completeness of clinical and ecological outcomes and for AE reporting will be summarised.

Patient characteristics for children recruited to the pilot cRCT will be compared with those for potentially eligible children within participating PICUs and within all UK PICUs.

Potential patient-centred clinical outcome measures for the definitive trial will be estimated and reported (proportion or mean and SD, and intracluster correlation). As a pilot cRCT, there will be no statistical testing for any of the summary measures. Comparisons between groups will be used to estimate the potential magnitude of the treatment effect; p values will not be calculated or quoted.^20^ To account for cluster randomisation, we will use multilevel logistic or generalised linear regressions in the above analyses.

### Mixed methods study

The mixed-methods study will involve a questionnaire and interviews with parents/legal guardians of children involved in the pilot cRCT as well as focus groups, interviews and an online survey with healthcare professionals. These will be used to review and explore:

- Parent views on.
  - The acceptability of a definitive trial that includes the SDD intervention.
  - The acceptability of the recruitment and consent procedures for the definitive trial, including all proposed information materials.
  - Important, relevant, patient-centred primary and secondary outcomes for a definitive trial.
- Healthcare professionals’ views on.
  - The acceptability of implementation of the SDD intervention, recruitment and consent procedures.
  - The acceptability of collecting data to assess the selected clinical and ecological data.
  - The acceptability of the SDD intervention and to confirm interest in participation in a definitive trial in the wider PICU community.

#### Inclusion criteria

- Parents/legal guardians of children involved in the pilot cRCT, including those who withdraw from data collection.
- Healthcare professionals (including doctors, nurses, physios, pharmacists) working in PICUs that participate in the pilot cRCT.

#### Exclusion criteria

- Parents/legal guardians who do not speak English.

#### Parents/legal representative recruitment and consent

Healthcare professionals will seek consent from parents of recruited children, including those who withdraw from data collection, to complete a questionnaire or register interest in a telephone or online interview. Questionnaires (n=~100) will be completed after pilot trial recruitment discussions and interviews will be conducted by the UoL team within a month until information power^21^ is reached (n=~15–25 based on previous studies)

#### Healthcare professionals’ recruitment and consent

Healthcare professionals involved in the pilot cRCT will be invited via email to participate in a virtual focus group (n=2) or interview (≥10 depending on information power). An online survey will be distributed through UK PICU networks.

#### Data analysis of mixed-methods study

Interviews and focus groups will be transcribed, checked and anonymised as the study progresses. QSR NVivo software will be used to assist in the organisation and indexing of qualitative data. While thematic analysis^22 23^ will draw on the Theoretical Framework of acceptability.^24 25^ The focus will be modified to fit with the criterion of catalytic validity, whereby findings should be relevant to future research and practice (in particular, the design of, and information to inform decisions on the progression to, a definitive cRCT). Quantitative data from parent questionnaires and the online survey will be analysed using SPSS software, descriptive statistics and exact tests will be used, as appropriate. Data from each method will be analysed separately then synthesised through the use of constant comparative analysis.^26^

## Ethics and dissemination

The PICnIC pilot study will be conducted in accordance with the approved Trial Protocol, ICH GCP guidelines, the Data Protection Act (2018), the Mental Capacity Act (2005), as well as the ICNARC CTU’s research policies and procedures.

The study received favourable ethical opinion from West Midlands—Black Country Research Ethics Committee (Ref: 20/WM/0061) and approval from the HRA (IRAS number: 239324).

Informed consent is not required for SDD intervention or anonymised data collection but is sought for investigations as part of the study, any identifiable data collected and monitoring of medical records.

The final report, including a detailed description of the trial, results and recommendations for future policy and practice and future research, will be submitted to the National Institute of Health Research Health Technology Assessment Programme. Articles will be prepared for publication in peer-reviewed scientific journals, as well as relevant professional journals.

### Oversight

The TMG, led by the chief investigator, is responsible for the management of PICnIC. It meets regularly and includes the Investigators and ICNARC CTU trial team. PICnIC is managed by the ICNARC CTU in accordance with the Medical Research Council’s Good Research Practice: Principles and Guidelines^27^ which is based on the ICH guidelines on GCP^28^ principles and the UK Department of Health’s Policy Framework for Health and Social Care Research.^29^

A majority independent Trial Steering Committee (TSC) has been established to monitor trial progress and includes PPI representatives, experienced clinicians and researchers/statisticians, in addition to the chief investigator and head of research at ICNARC. An independent DMEC, comprising experienced clinicians and statisticians, has been established to monitor patient recruitment and retention, adherence and safety.

Cambridge University Hospitals National Health Service (NHS) Foundation Trust and The University of Cambridge is the trial sponsor. As the sponsor is an NHS organisation, NHS indemnity will apply for legal liability arising from the design, management and conduct of the research.

Ownership of the data sits with the sponsor with collaboration agreements in place to allow access to necessary partners on the grant. Once a final anonymised dataset is created at the end of the study, requests for access to data will be reviewed and approved by the TMG.

### Trial status

The paper presents protocol V.4.1, dated 17 December 2021. At the time of submission, patient recruitment was ongoing. Recruitment commenced in September 2021 with recruitment planned to complete in February 2022. Follow-up data collection will continue until the end of March 2022.

## Supplementary Material

Reviewer comments

Author's manuscript

## References

[1] Patrick SW, Kawai AT, Kleinman K, et al. Health care-associated infections among critically ill children in the US, 2007-2012. Pediatrics 2014;134:705–12. 10.1542/peds.2014-0613 25201802

[2] Field-Ridley A, Dharmar M, Steinhorn D, et al. ICU-Acquired weakness is associated with differences in clinical outcomes in critically ill children. Pediatr Crit Care Med 2016;17:53–7. 10.1097/PCC.0000000000000538 26492063 PMC5008971

[3] Choong K, Al-Harbi S, Siu K, et al. Functional recovery following critical illness in children: the "wee-cover" pilot study. Pediatr Crit Care Med 2015;16:310–8. 10.1097/PCC.0000000000000362 25651047 PMC4499478

[4] Heyland DK, Cook DJ, Griffith L, et al. The attributable morbidity and mortality of ventilator-associated pneumonia in the critically ill patient. The Canadian critical trials Group. Am J Respir Crit Care Med 1999;159:1249–56. 10.1164/ajrccm.159.4.9807050 10194173

[5] Bekaert M, Timsit J-F, Vansteelandt S, et al. Attributable mortality of ventilator-associated pneumonia. Am J Respir Crit Care Med 2011;184:1133–9. 10.1164/rccm.201105-0867OC 21852541

[6] J Francis J, M Duncan E, E Prior M, et al. Selective decontamination of the digestive tract in critically ill patients treated in intensive care units: a mixed-methods feasibility study (the SuDDICU study). Health Technol Assess 2014;18:1–170. 10.3310/hta18250 PMC4967810 24775071

[7] de Smet AMGA, Kluytmans JAJW, Cooper BS, et al. Decontamination of the digestive tract and oropharynx in ICU patients. N Engl J Med 2009;360:20–31. 10.1056/NEJMoa0800394 19118302

[8] Canter RR, Harvey SE, Harrison DA, et al. Observational study of current use of selective decontamination of the digestive tract in UK critical care units. Br J Anaesth 2014;113:610–7. 10.1093/bja/aeu108 24829442

[9] Daneman N, Sarwar S, Fowler RA, et al. Effect of selective decontamination on antimicrobial resistance in intensive care units: a systematic review and meta-analysis. Lancet Infect Dis 2013;13:328–41. 10.1016/S1473-3099(12)70322-5 23352693

[10] Liberati A, D'Amico R, Torri V. Antibiotic prophylaxis to reduce respiratory tract infections and mortality in adults receiving intensive care. Cochrane Database Syst Rev 2004;1:Cd000022. 10.1002/14651858.CD000022.pub2 14973945

[11] Oostdijk EAN, de Smet AMGA, Blok HEM, et al. Ecological effects of selective decontamination on resistant gram-negative bacterial colonization. Am J Respir Crit Care Med 2010;181:452–7. 10.1164/rccm.200908-1210OC 19965807

[12] Plantinga NL, Bonten MJM. Selective decontamination and antibiotic resistance in ICUs. Crit Care 2015;19:259. 10.1186/s13054-015-0967-9 26104045 PMC4479224

[13] Price RJ, Cuthbertson BH, SuDDICU collaboration. Selective decontamination of the digestive tract and oropharynx: after 30 years of debate is the definitive answer in sight? Curr Opin Crit Care 2016;22:161–6. 10.1097/MCC.0000000000000281 26766392

[14] Wittekamp BH, Plantinga NL, Cooper BS, et al. Decontamination strategies and bloodstream infections with antibiotic-resistant microorganisms in ventilated patients: a randomized clinical trial. JAMA 2018;320:2087–98. 10.1001/jama.2018.13765 30347072 PMC6583563

[15] Billot L, Cuthbertson BH, et al. Protocol summary and statistical analysis plan for the selective decontamination of the digestive tract in intensive care unit patients (SuDDICU) crossover, cluster randomised controlled trial. Crit Care Resusc 2021;23. 10.51893/2021.2.oa5 PMC10692556 38045525

[16] Petros A, Silvestri L, Booth R, et al. Selective decontamination of the digestive tract in critically ill children: systematic review and meta-analysis. Pediatr Crit Care Med 2013;14:89–97. 10.1097/PCC.0b013e3182417871 22805154

[17] Woolfall K, Young B, Frith L, et al. Doing challenging research studies in a patient-centred way: a qualitative study to inform a randomised controlled trial in the paediatric emergency care setting. BMJ Open 2014;4:e005045. 10.1136/bmjopen-2014-005045 PMC4025463 24833694

[18] Weijer C, Grimshaw JM, Eccles MP, et al. The Ottawa statement on the ethical design and conduct of cluster randomized trials. PLoS Med 2012;9:e1001346. 10.1371/journal.pmed.1001346 23185138 PMC3502500

[19] Nix HP, Weijer C, Brehaut JC, et al. Informed consent in cluster randomised trials: a guide for the perplexed. BMJ Open 2021;11:e054213–e13. 10.1136/bmjopen-2021-054213 PMC8477335 34580104

[20] Chan CL, Leyrat C, Eldridge SM. Quality of reporting of pilot and feasibility cluster randomised trials: a systematic review. BMJ Open 2017;7:e016970. 10.1136/bmjopen-2017-016970 PMC5695336 29122791

[21] Malterud K, Siersma VD, Guassora AD. Sample size in qualitative interview studies: guided by information power. Qual Health Res 2016;26:1753–60. 10.1177/1049732315617444 26613970

[22] Boyatzis R. Transforming qualitative information: thematic analysis and code development, 1998.

[23] Braun V, Clarke V. Using thematic analysis in psychology. Qual Res Psychol 2006;3:77–101. 10.1191/1478088706qp063oa

[24] Deja E, Peters MJ, Khan I, et al. Establishing and augmenting views on the acceptability of a paediatric critical care randomised controlled trial (the fever trial): a mixed methods study. BMJ Open 2021;11:e041952–e52. 10.1136/bmjopen-2020-041952 PMC7949453 33692177

[25] Sekhon M, Cartwright M, Francis JJ. Acceptability of healthcare interventions: an overview of reviews and development of a theoretical framework. BMC Health Serv Res 2017;17:88. 10.1186/s12913-017-2031-8 28126032 PMC5267473

[26] Glaser BG. The constant comparative method of qualitative analysis. Soc Probl 1965;12:436–45. 10.2307/798843

[27] Medical Research Council good research practice: principles and guidelines, 2012, 2012. Available: https://mrc.ukri.org/publications/browse/good-research-practice-principles-and-guidelines/

[28] Academic. International conference on harmonisation of technical requirements for registration of pharmaceuticals for human use. guideline for good clinical practice, 1996.

[29] Health Research Authority. UK policy framework for health and social care research, 2017. Available: https://www.hra.nhs.uk/planning-and-improving-research/policies-standards-legislation/uk-policy-framework-health-social-care-research/

