# Supplementary material for: Use of selective gut decontamination in critically ill children: protocol for the Paediatric Intensive Care and Infection Control (PICnIC) pilot study
Source: BMJ Open. 2022 Mar 11;12(3):e061838. doi: 10.1136/bmjopen-2022-061838 (PMC8919465; doi:10.1136/bmjopen-2022-061838)
Supplement: Supplementary data [file bmjopen-2022-061838supp001.pdf]

Supplementary file 1 for protocol paper:

The use of selective gut decontamination in critically ill children: Paediatric Care and Infection Control (PICnIC): A protocol of a pilot cluster randomised trial

How does SDD work?

SDD paste and SDD suspension are both formulations containing three non-absorbable antibiotics and antifungals: colistin (polymyxin E), tobramycin and nystatin. All three are licensed antibacterial and antifungal drugs, and are currently used in critically ill patients when indicated.

In SDD therapy, the drugs are used to eradicate the gastro-intestinal carriage of potentially pathogenic micro-organisms including Staphylococcus aureus, methicillin-resistant Staphylococcus aureus (MRSA), aerobic Gram-negative bacilli and yeasts.[1]

The drugs are non-absorbable, ensuring that their concentrations remain high enough in the mouth and into the colon to selectively eradicate the carriage of pathogenic micro-organisms, whilst not influencing the protective anaerobic flora, thereby decreasing colonisation resistance.[1]

Colistin is a multicomponent antibiotic. It is a mixture of several closely related decapeptides (polymyxin E). The main components are polymyxin E1 and E2. Colistin has an antimicrobial spectrum and mode of action similar to that of polymyxin B, but is slightly less active. It is bactericidal to most Gram-negative bacteria.[1]

Tobramycin sulphate is an aminoglycoside antibiotic with good aqueous solubility. It acts against many strains of Gram-negative bacteria, including pseudomonas aeruginosa.[1]

Colistin and tobramycin act in synergy against proteus and pseudomonas species, and offer the most potent anti-pseudomonal combination, with an effective clearance of pseudomonas from the gut. Both agents absorb endotoxin released by aerobic Gram-negative bacilli. This feature is important because endotoxin can be absorbed into the bloodstream from the gut of seriously ill patients. This contributes to fever, inflammatory activation, shock and organ failure.[1,2]

Emergence of resistance to colistin is rare. Although there are bacteria producing tobramycin-inactivating enzymes, colistin is thought to protect tobramycin from being destroyed by these enzymes. Tobramycin is intrinsically the most active aminoglycoside against pseudomonas, and is minimally inactivated by saliva and faeces.

In critically ill patients, the use of intravenous antimicrobials suppress the patient's commensal species in the gastrointestinal tract. This is associated with overgrowth of extended-spectrum beta-lactamase (ESBL)-producing aerobic Gram-negative bacilli in the gut. The action of enteral colistin and tobramycin against aerobic Gram-negative bacilli prevents the persistence of ESBL producing aerobic Gram-negative bacilli.[3]

Nystatin is a non-absorbable polyene drug with wide antifungal activity, especially against candida species. It significantly reduces fungal carriage and overall fungal infections and is less likely to promote the emergence of resistant candidal strains compared to other antifungal agents.[4] It also has advantages of low cost and absence of side effects.[4]

The most widely used SDD regimen to date uses amphotericin B in combination with polymyxin (colistin) and tobramycin. For PICnIC, nystatin is used in place of amphotericin B

due to difficulties sourcing amphotericin B. Nystatin, like amphotericin B is a non-absorbable polyene with wide antifungal activity.[4]

How is SDD administered?
Every six hours a 'pea-sized' amount (0.5 g) of SDD paste will be topically applied to the buccal mucosa and oropharynx and an SDD suspension is administered to the gastrointestinal tract via the most proximal feeding tube, with dosing according to age (Supplementary Table 1):

Supplementary Table 1: SDD suspension dosing

	0 – 4 years	5 – 12 years	≥13 years
**Polymyxin E (Colistin)**	25mg	50mg	100mg
**Tobramycin**	20mg	40mg	80mg
**Nystatin**	0.5 x 10^6 IU	1 x 10^6 IU	2 x 10^6 IU
	**2.5ml**	**5ml**	**10ml**

Similarity to other compounds
The most widely used SDD regimen to date is a combination of polymyxin E (colistin), tobramycin and amphotericin B applied as an oral paste and suspension to treat both the throat and gut, respectively.

For PICNIC, nystatin will be used in place of amphotericin B due to difficulties sourcing amphotericin B. Nystatin, like amphotericin B is a non-absorbable polyene with wide antifungal activity.[5] Nystatin may prevent the emergence of resistant fungal strains such as candida species and has advantages such as its low cost and absence of side effects.

Chemistry, Manufacturing and controls.
The SDD paste and suspension has been manufactured in accordance with Good Laboratory Practice (GLP) and Good Manufacturing Practice (GMP) for clinical studies (See Appendix 1 for Verita Pharma GMP licence).

Development plan
An unpublished, inception cohort pilot study testing inclusion criteria and examining process measures and outcomes was conducted in an adult population by collaborators at The George Institute and informed the development of the PICnIC protocol. The outcomes of this study will inform the development of a full trial within paediatrics.

Physical, Chemical and pharmaceutical properties and formulation.
Study Drugs.
The active pharmaceutical ingredients of both the SSD paste (Supplementary Table 2) and SDD suspension (Supplementary Table 3) are colistin sulphate, tobramycin sulphate and nystatin.

Supplementary Table 2: Composition and Characteristics of SDD paste.

Name	SDD paste
International nomenclature	The paste is a semi-solid dosage forms containing finely dispersed solids, have a stiff consistency, and is intended for topical application.<br>Active pharmaceutical ingredients:<br>Colistin sulphate<br>Tobramycin (as sulphate)<br>Nystatin
Sponsor name	The George Institute for Global Health
Chemical abstract service number	Colistin : 1264-72-8<br>Tobramycin: 49842-07-1<br>Nystatin: 1400-61-9
Chemical structure	Colistin Sulphate 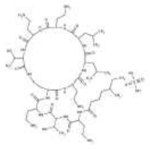<br>Tobramycin 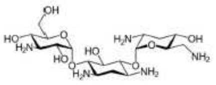<br>Nystatin 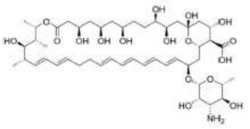
Molecular formula	Colistin : $2(C_{52}H_{98}N_{16}O_{13}).5(H_2SO_4)$<br>Tobramycin: $C_{18}H_{37}N_5O_9$<br>Nystatin: $C_{47}H_{75}NO_{17}$
Molecular weight	Colistin: 2801 g/mol<br>Tobramycin: 467.5 g/mol<br>Nystatin: 926.1 g/mol
Description	SDD paste is a mixture of antimicrobial powders – colistin sulphate, tobramycin sulphate and nystatin- with mineral oil light, petrolatum white and methocel E4M premium in an oral syringe.
Odour	Not applicable
Solubility	Not applicable
Properties	A smooth, yellow paste of uniform consistency

Supplementary Table 3: Composition and characteristics of SDD suspension.

Name	SDD suspension
International nomenclature	The suspensions is liquid preparations containing drug substance(s) and consist of solid particles dispersed throughout a liquid phase in which the particles are present in excess of the solubility. Some suspensions are prepared and ready for use, while others are solid mixtures intended for constitution before use with an appropriate vehicle.
Sponsor name	As per SDD paste
Chemical abstract service number	As per SDD paste
Structure	As per SDD paste
Molecular formula	As per SDD paste
Molecular weight	As per SDD paste
Description	SDD powder for suspension is a blend of antimicrobial powders- colistin sulphate, tobramycin sulphate and nystatin with a suspending agent (syrspend pH 4 dry), a preservative (potassium sorbate) and citric acid monohydrate in a bottle. Before use, each bottle is reconstituted with purified/distilled water to the required volume and then shaken.
Odour	Not applicable
Solubility	Part of the powder will be dissolved and part suspended
Properties	SDD suspension; after suspending: an opaque, straw coloured liquid

Manufacture.
The SDD paste and SDD suspension are manufactured through a series of proprietary processing steps and performed in accordance with GLP/GMP under licence at:
Verita Pharma Pty LTD
Unit 3, 4 Endeavour Rd
Taren Point
NSW 2229
Australia

Analysis and characterisation of study drug.
The identity of each container within each batch of raw material destined for study drug is confirmed by full pharmacopoeia analysis by validated high-performance liquid chromatography (HPLC) and microbiology methodology (in accordance with annex 8 of Pharmaceutical Inspection Co-operation Scheme guide to GMP manufacturing).

Homogeneity and potency of each finished product batch of investigational product is confirmed via similar methodology.

Impurities will be assessed by Verita Pharma using HPLC and other analytical methods during on-going stability studies.

Stability
The manufacturer stability testing program is focused on determining allowable shelf-life and ensuring the product maintains compliance with the claims made on the SDD paste and SDD suspension labels when exposed to routine usage and storage conditions.

Stability testing uses HPLC and microbiology analyses to ensure that study drugs remains homogenous, with potency within specifications and exhibits normally expected physical

characteristics, when stored for defined durations, at the ranges of temperature and subjected to reasonably expected temperature excursions.

Investigational products

Formulation.

SDD paste

The clinical products are formulated by combining the ingredients shown in Supplementary Table 4 using a series of proprietary processing steps prior dispensing into 1ml oral syringes. SDD paste is formulated to contain per 0.5g paste: colistin sulphate 10 mg tobramycin 10 mg and nystatin 125,000 IU.

Supplementary Table 4: General Investigational drug product information.

Ingredient	Specification	Purpose	Conc. (per gram)
Colistin sulphate	BP	Active	20mg
Nystatin	Eur. Ph. 8th ed. (BP)	Active	0.25mu
Tobramycin (as sulphate)	USP	Active	20mg
Mineral oil light	USP	Excipient	50mg
Methocel e4m premium	USP	Excipient	177mg
Petrolatum white	USP	Excipient	686mg*

BP = British Pharmacopoeia provides quality standards for UK pharmaceutical substances and medicinal products
Eur.Ph.8th ed.= European Pharmacopoeia 8th Edition is Europe's scientific and legal benchmark for pharmacopoeia standards
USP = United States Pharmacopeia has established standards for manufacturing and supplying drugs worldwide
Active = an active component in a medicines final formulation
Excipient = an excipient is an inactive substance for the purpose of bulking-up drug formulations
Methocel e4m premium = a renewable raw material, are water soluble polymer derided from cellulose
Petrolatum white = semi-solid mixture of hydrocarbons.
* For 'petrolatum white' quantity added to batch is calculated to quantum satis

SDD suspension

The clinical products are formulated by combining the ingredients shown in Supplementary Table 5 using a series of proprietary processing steps and dispensing into defined aliquots within each polyethylene terephthalate (PET) bottle, for storage. When the product is required for the trial, each bottle of antimicrobial powder is reconstituted to the desired volume with purified/distilled water. The formulation includes a suspending agent (SyrSpend sf ph4) to ensure that when the bottle is standing, the active powder (Nystatin) is evenly dispersed after shaking between administrations to the patient, thereby preventing 'caking' on the bottom of the bottle. Due to the difficulties experienced at participating sites in achieving consistent reconstitution and the increased viscosity (thickening) of the suspension after 3 days, the formulation was modified in September 2018 to reduce the amount of SyrSpend by 30%. The removal of 30% suspending agent has no influence on product efficacy, chemical attributes or on the active components.

Supplementary Table 5: General investigational drug product information

Ingredient	Specification	Purpose	Concentration After reconstitution per ml
Colistin sulphate	BP	Active	10mg
Nystatin	Eur. Ph. 8th ed. (BP)	Active	0.20mu
Tobramycin (as sulphate)	USP	Active	8mg
Syrspend sf ph4	USP	Excipient	45.5mg
Citric acid monohydrate	BP	Excipient	2.86mg
Potassium sorbate	USP	Preservative	2.0mg

BP = British Pharmacopoeia provides quality standards for UK pharmaceutical substances and medicinal products
Eur.Ph.8th ed. = European Pharmacopoeia 8th Edition is Europe's scientific and legal benchmark for pharmacopoeia standards
USP = United States Pharmacopeia has established standards for manufacturing and supplying drugs worldwide
Active = an active component in a medicines final formulation
Excipient = an excipient is an inactive substance for the purpose of bulking-up drug formulations
Preservative = a natural or synthetic chemical that prevents decomposition by microbial growth or by undesirable chemical changes
Syrspend sf ph4 = is a ready-to-use, all-in-one suspending and sweetening oral liquid
Citric acid monohydrate = a tricarboxylic acid and maintains stability of active ingredients
Potassium sorbate = is the potassium salt of sorbic acid, commonly used preservative in the conservation of liquid pharmaceutical preparations.

Final dosage form and presentation
SDD paste (0.5g) is supplied in 1ml BD oral syringe and is stored at 2-8°C. During patient use the paste can be stored in a temperature-controlled room of less than 25°C for up to one week. SDD suspension is supplied in antimicrobial powder form in a sealed PET bottle with a syringe filling adaptor under a child resistant screwcap and is stored at 2-8°C. After reconstitution of the powder, the suspension can be stored in a temperature-controlled room of less than 25°C for up to one week.

Container and packaging
Each SDD study drug kit contains, twenty 1ml BD oral syringes SDD paste, a single sealed PET bottle of SDD suspension (powder form) and a syringe filling adaptor and shipped under refrigerated/cold chain 2-8˚C (35°–46°F) conditions to the clinical trial site.

Storage and handling
The SDD study drug kits are to be stored at 2–8°C (35°–46°F) in a secure area. When SDD powder is reconstituted into suspension the drug kit (paste and suspension) can be stored in a temperature controlled room of less than 25°C for up to 1 week. Each pre-filled syringe is to be discarded after use (i.e. single use only). The PET bottle is to be returned to the SDD study drug kit after each dose. Hospitals will need to provide their own 10ml oral/enteral syringe to access and measure each dose from the PET bottle (which contains a syringe filling adaptor).

Shelf life
There is no information in the literature regarding stability testing of drugs containing colistin sulphate, tobramycin sulphate and nystatin as this is a new formulation. In reference literature, data concerning stability testing of drugs containing polymyxin/tobramycin/amphotericin - the most widely used SDD regimen to date – is limited.

Stability determinations/concentrations have therefore been assessed by HPLC and microbiology methods. Test methods have been developed and validated, and real-time ageing tests conducted to define safe storage parameters, including reasonably expected excursions.

The stability program for the SDD paste and SDD suspension is continually ongoing. The expiry date on the label will reflect the most recent shelf-life determination of the product contained within the syringe/bottle.
Current stability testing has demonstrated that:

1. SDD paste is stable at 2-8°C for up to 12 months.
2. SDD suspension is stable at 2-8°C for up to 2 weeks.
3. SDD powder for suspension is stable at 2-8°C for up to 12 months.
4. SDD paste, SDD powder for suspension and SDD suspension can be stored below 25°C for short excursions up to one week. (There is stability evidence up to one month, but this is not appropriate for the suspension because it is 'in use' at this stage. Suspension should be used as per the protocol once reconstituted.)

Refer to Appendices 2-8 for current stability results for SDD paste, SDD powder and SDD suspension.

Reference list

1. van Saene HKF, Silvestri L, de la Cal MA, Gullo A, editors. Infection Control in the Intensive Care Unit [Internet]. Milano: Springer Milan; 2012 [cited 2018 Oct 26]. Available from: http://link.springer.com/10.1007/978-88-470-1601-9
2. Pathan N, Burmester M, Adamovic T, Berk M, Ng KW, Betts H, et al. Intestinal injury and endotoxemia in children undergoing surgery for congenital heart disease. Am J Respir Crit Care Med [Internet]. 2011;184(11):1261–9. Available from: http://www.ncbi.nlm.nih.gov/pubmed/21868501
3. van der Spoel HI, Gerritsen RT. SDD for the Prevention and Control of Outbreaks. Selective Digestive Tract Decontamination in Intensive Care Medicine: a Practical Guide to Controlling Infection [Internet]. Milano: Springer Milan; 2008 [cited 2018 Oct 26]. p. 141–54. Available from: http://link.springer.com/10.1007/978-88-470-0653-9_11
4. Normand S, François B, Dardé M-L, Bouteille B, Bonnivard M, Preux P-M, et al. Oral nystatin prophylaxis of Candida spp. colonization in ventilated critically ill patients. Intensive Care Med [Internet]. 2005 Nov 30 [cited 2018 Oct 26];31(11):1508–13. Available from: http://www.ncbi.nlm.nih.gov/pubmed/16195905
5. Ruza F, Alvarado F, Herruzo R, Delgado MA, García S, Dorao P, et al. Prevention of nosocomial infection in a pediatric intensive care unit (PICU) through the use of selective digestive decontamination. Eur J Epidemiol [Internet]. 1998 Oct [cited 2018 Oct 25];14(7):719–27. Available from: http://www.ncbi.nlm.nih.gov/pubmed/9849834

Appendices

1. Verita Pharma most recent GMP Licence-MI-2016-LI-07350, dated 23 July 2020

Australian Government
Department of Health
Therapeutic Goods Administration

Licence to Manufacture Therapeutic Goods – Part 1

Licence Number:
MI-2016-LI-07350-1

Granted to:
Verita Pharma Pty Ltd
ABN: 89 604 520 532

Manufacturing Site Address:
Lot 8, 10 and 11 (SP92206), 2 Endeavour Road
Caringbah NSW 2229

The manufacturer above is hereby authorised under Section 38 of the *Therapeutic Goods Act 1989* to carry out the following steps in the manufacture of therapeutic goods at the manufacturing site address specified above.

Manufacturing Type	Sterility	Dosage Form	Product Category	Manufacturing Step
Medicine manufacture	Non Sterile	Semi Solids	Therapeutic Goods for Clinical Trials	Finished Product Manufacture
Medicine manufacture	Non Sterile	Semi Solids	Registered Therapeutic Good	Finished Product Manufacture
Medicine manufacture	Non Sterile	Powders Group	Therapeutic Goods for Clinical Trials	Finished Product Manufacture
Medicine manufacture	Non Sterile	Powders Group	Registered Therapeutic Good	Finished Product Manufacture
Medicine manufacture	Non Sterile	Oral Liquid, suspension	Therapeutic Goods for Clinical Trials	Finished Product Manufacture
Medicine manufacture	Non Sterile	Oil	Therapeutic Goods for Clinical Trials	Finished Product Manufacture
Medicine manufacture	Non Sterile	Spray	Therapeutic Goods for Clinical Trials	Finished Product Manufacture
Medicine manufacture	Sterile & Non Sterile	All Dosage Forms	Not Applicable	Testing chemical and physical

This Licence is the property of the Therapeutic Goods Administration and must be returned or destroyed upon demand.
This Licence remains valid until otherwise suspended or revoked by the Therapeutic Goods Administration.
The status of an Australian Licence may be viewed at https://www.ebs.tga.gov.au/

PO Box 100 Woden ACT 2606 ABN 40 939 406 804
Phone: 1800 020 653 Fax: 02 6203 1605 Email: info@tga.gov.au www.tga.gov.au

TGA Health Safety Regulation

Page 1 of 2

Australian Government
Department of Health
Therapeutic Goods Administration

Licence to Manufacture Therapeutic Goods – Part 1

Licence Number:
MI-2016-LI-07350-1

Manufacturing Type	Sterility	Dosage Form	Product Category	Manufacturing Step
Medicine manufacture	Sterile	Injections	Compounded Medicine	Sterile Finished Product Manufacture
Medicine manufacture	Sterile	Injections	Therapeutic Goods for Clinical Trials	Sterile Finished Product Manufacture

This licence is subject to the requirements of the *Therapeutic Goods Act1989*, and its Regulations.

Section 40(4) of the *Therapeutic Goods Act 1989* and Regulation 19, 20, 21 and 22 of the Therapeutic Goods Regulations 1990 impose various statutory conditions on all licences to manufacture therapeutic goods.

In addition to that, the specific conditions mentioned in Part 2 of this licence have been imposed under Section 40(1) or 40(2) of the *Therapeutic Goods Act 1989*.

Originally Granted: **16 May 2017** Date Revised: **23 July 2020**

This Licence is the property of the Therapeutic Goods Administration and must be returned or destroyed upon demand.
This Licence remains valid until otherwise suspended or revoked by the Therapeutic Goods Administration.
The status of an Australian Licence may be viewed at https://www.ebs.tga.gov.au/

PO Box 100 Woden ACT 2606 ABN 40 939 406 804
Phone: 1800 020 653 Fax: 02 6203 1605 Email: info@tga.gov.au www.tga.gov.au

TGA Health Safety Regulation

Page 2 of 2

2. SDD Oral Paste 5°C R&D Stability Summary

Form No: TF2402-01

R&D Stability Report

Product Name: **SDD Oral Paste**	Product Code: **FP002**	Company Name: **The George Institute**
Dosage Form: **Paste**	Batch No: **241117**	Stability Study: **SP-0001-03**
Packaging: **BD oral/enteral 1 ml syringe and tip cap**	Storage Temperature: **5°C ± 3°C**	Humidity: **N/A**

**Time points**		**Initial**	**2 w**	**4 w**	**12 w**	**16 w**	**6 M**	**9 M**	**12M**
**Due date**		**27/11/17**	**11/12/17**	**08/01/18**	**19/02/18**	**19/03/18**	**27/05/18**	**27/08/18**	**27/11/18**
**Test**	**Specifications**								
**Appearance**	Light yellow paste	**complies**	**complies**	**complies**	**complies**	**complies**	**complies**		**complies**
**Viscosity**	TBD	**128,000 cps**	**157,000 cps**	**158,000 cps**	**150,333 cps**	**198,000 cps**	**198,000 cps**		**180,000 cps**
**Nystatin Assay**	250,000 IU/g<br>*Assay precision fiducial limits: 96-106%*<br>*UFL: NLT 90% of stated IU*<br>*LFL: NMT 115 % of stated IU*	**243,621 IU/g**	***Not available (*)***	**242,806 IU/g**	**243,996 IU/g**	**244,270 IU/g**	**249,698 IU/g**		**245,342 IU/g**
**Colistin Sulphate Assay**	20 mg/g<br>*Assay precision fiducial limits: 96-106%*<br>*UFL: NLT 97% of stated mg*<br>*LFL: NMT 110 % of stated mg*	**20.1 mg/g**	***Not available (*)***	**19.5 mg/g**	**19.6 mg/g**	**19.7 mg/g**	**19.9 mg/g**	**Cancelled**	**20.0 mg/g**
**Tobramycin Assay**	20 mg/g<br>*18 mg/g – 24 mg/g*	**20.24 mg/g**	**21.0 mg/g**	**20.4 mg/g**	**20.56 mg/g**	**21.20 mg/g**	**21.65 mg/g**		**20.20 mg/g**
**Microbiological quality**	Complies<br>TAMC: 10^3 CFU/g<br>TYMC: 10^2 CFU/g<br>*S aureus*: Absence/g<br>*P aeruginosa*: Absence/g	**Complies**			**complies**	**complies**			**complies**
**PET**	Complies								**Complies USP**
**Date completed**		**19/12/17**	**28/12/17**	**17/01/18**	**08/03/18**	**10/04/18**	**14/06/18**		**05/04/2019**

Comments: ***The tests with (*) have not been performed.***

Checked By:________________________ (Sign and Date) **Authorised By QA Manager:**__________________________ (Sign and Date)

3. SDD Oral Paste 25°C-60%RH R&D Stability

Form No: TF2402-01

R&D Stability Report

Product Name: **SDD Oral Paste**	Product Code: **FP002**	Company Name: **The George Institute**
Dosage Form: **Paste**	Batch No: **241117**	Stability Study: **SP-0001-03**
Packaging: **BD oral/enteral 1 ml syringe and tip cap**	Storage Temperature: **25°C ± 2°C**	Humidity: **60 %RH ± 5% RH**

Time points		Initial	8 weeks	12 weeks					
Due date		27/11/17	22/01/18	19/02/18					
**Test**	**Specifications**								
**Appearance**	Light yellow paste	complies	complies	complies					
**Viscosity**	TBD	128,000 cps	124,000 cps	123,000 cps					
**Nystatin Assay**	250,000 IU/g<br>*Assay precision fiducial limits: 96-105%*<br>*UFL: NLT 90% of stated IU*<br>*LFL: NMT 115 % of stated IU*	243,621 UI/g	241.968 UI/g	241.161 UI/g					
**Colistin Sulphate Assay**	20 mg/g<br>*Assay precision fiducial limits: 96-105%*<br>*UFL: NLT 97% of stated mg*<br>*LFL: NMT 110 % of stated mg*	20.1 mg/g	19.8 mg/g	19.7 mg/g					
**Tobramycin Assay**	20 mg/g<br>*18 mg/g – 24 mg/g*	20.24 mg/g	20.40 mg/g	20.87 mg/g					
**Microbiological quality**	Complies<br>TAMC: 10^3 CFU/g<br>TYMC: 10^2 CFU/g<br>*S aureus*: Absence/g<br>*P aeruginosa*: Absence/g	Complies		Complies					
**PET**	Complies USP requirements		Complies						
**Date completed**		19/12/17	08/03/18	08/03/18					

Comments: ____________________

Checked By:____________________ (Sign and Date) **Authorised By QA Manager:**____________________ (Sign and Date)

4. SDD Powder for Suspension 5°C R&D Stability

Form No: TF2402-01

VERITA PHARMA

R&D Stability Report

Product Name: **SDD Powder for Suspension**	Product Code: **FP001**	Company Name: **The George Institute**
Dosage Form: **Powder**	Batch No: **Int001071117**	Stability Study: **SP-0002-05**
Packaging: **Amber Plastic bottle with Child Resistant Cap**	Storage Temperature: **5°C ± 3°C**	Humidity: **N/A**

Time points		Initial	2 w	4 w	9 w	12 w	18 w	6 M	9 M	12 M
Due date		15/11/17	29/11/17	13/12/17	17/01/18	07/02/18	21/03/18	15/05/18	15/08/18	15/11/18
Test	Specifications									
Appearance	pale yellow powder	complies	complies	complies	complies	complies	complies	complies	Cancelled	complies
Loss on Drying	TBD	5.63 %	4.98 %	6.47 %	5.19 %	5.72 %	5.14 %	6.52 %		5.75 %
pH *(after reconstitution)*	4.0 – 5.0	4.49	4.71	4.63	4.58	4.45	4.32	4.54		4.53
Nystatin Assay *(after reconstitution)*	200,000 IU/ml *Assay precision fiducial limits: 95-105% UFL: NLT 95% of stated IU LFL: NMT 120 % of stated IU*	206,182 IU/ml	200,288 IU/ml	*Not available (*)*	205.602 IU/ml	203,390 IU/ml	200,970 IU/ml	205,321 IU/ml		202,614 IU/ml
Colistin Sulphate Assay *(after reconstitution)*	10 mg/ml *Assay precision fiducial limits: 95-105% UFL: NLT 97% of stated mg LFL: NMT 110 % of stated mg*	10.2 mg/ml	9.8 mg/ml	*Not available* (*)	9.5 mg/ml	9.9 mg/ml	9.9 mg/ml	10.2 mg/ml		10.1 mg/ml
Tobramycin Assay *(after reconstitution)*	8 mg/ml *7.2 mg/g –9.6 mg/g*	8.73 mg/ml	8.87 mg/ml	8.84mg/ml	8.66 mg/ml	8.85 mg/ml	8.18 mg/ml	8.75 mg/ml		8.09 mg/ml
Microbiological quality *(after reconstitution)*	Complies TAMC: 10^2 CFU/g TYMC: 10^1 CFU/g *E.coli*: Absence/g	complies				complies	complies			complies
PET *(after reconstitution)*	Complies									complies
Date completed		05/12/17	22/12/17	02/01/18	19/01/18	08/03/18	12/04/18	13/06/18		29/01/2019

Comments: ***The tests with (*) have not been performed.***

Checked By:________________________ (Sign and Date) **Authorised By QA Manager:**__________________________ (Sign and Date)

5. SDD Powder for Suspension 25°C-60%RH R&D Stability

Form No: TF2402-01

VERITA PHARMA

R&D Stability Report

Product Name: **SDD Powder for Suspension**	Product Code: **FP001**	Company Name: **The George Institute**
Dosage Form: **Powder**	Batch No: **Int001071117**	Stability Study: **SP-0002-05**
Packaging: **Amber Plastic bottle with Child Resistant Cap**	Storage Temperature: **25°C ± 2°C**	Humidity: **60% RH**

**Time points**		**Initial**	**8 w**	**12 w**					
**Due date**		**15/11/17**	**10/01/18**	**07/02/18**					
**Test**	**Specifications**								
**Appearance**	pale yellow powder	**complies**	**complies**	**complies**					
**Loss on Drying**	TBD	**5.63**	**5.72**	**5.29**					
**pH** *(after reconstitution)*	4.0 – 5.0	**4.49**	**4.64**	**4.68**					
**Nystatin Assay** *(after reconstitution)*	200,000 IU/ml *Assay precision fiducial limits: 95-105%* *UFL: NLT 95% of stated IU* *LFL: NMT 120 % of stated IU*	**206,182 IU/ml**	**203,811 IU/ml**	**199,917 IU/ml**					
**Colistin Sulphate Assay** *(after reconstitution)*	10 mg/ml *Assay precision fiducial limits: 95-105%* *UFL: NLT 97% of stated mg* *LFL: NMT 110 % of stated mg*	**10.2 mg/ml**	**10.0 mg/ml**	**9.8 mg/ml**					
**Tobramycin Assay** *(after reconstitution)*	8 mg/ml *7.2 mg/g –9.6 mg/g*	**8.73 mg/ml**	**8.65 mg/ml**	**8.57 mg/ml**					
**Microbiological quality** *(after reconstitution)*	Complies TAMC: 10^2 CFU/g TYMC: 10^1 CFU/g *E.coli*: Absence/g	**complies**		**complies**					
**PET** *(after reconstitution)*	Complies		**Complies**						
**Date completed**		**05/12/17**	**08/03/18**	**08/03/18**					

Comments: ____________________

Checked By:____________________ (Sign and Date) **Authorised By QA Manager:**____________________ (Sign and Date)

6. SDD Suspension 5°C R&D Stability

Form No: TF2402-01

VERITA PHARMA

R&D Stability Report

Product Name: **SDD Suspension**	Product Code: **N/A**	Company Name: **The George Institute**
Dosage Form: **Suspension**	Batch No: **FP001071117**	Stability Study: **SP-0003-02**
Packaging: **Amber Plastic bottle with Child Resistant Cap**	Storage Temperature: **5°C ± 3°C**	Humidity: **N/A**

Time points		Initial	1 w	2 w	3 w	1 M	1.5 M	2 M	
Due date		15/11/17	22/11/17	29/11/17	06/12/17	15/12/17	01/01/18	15/01/18	
**Test**	**Specifications**								
**Appearance**	After shaking, a yellow liquid	**complies**	**complies**	**complies**	**complies**	**complies**	**complies**	**complies**	
**pH** *(after reconstitution)*	4.0 – 5.0	**4.50**	**4.47**	**4.54**	**4.47**	**4.61**	**4.45**	**4.55**	
**Nystatin Assay** *(after reconstitution)*	200,000 IU/ml *Assay precision fiducial limits: 95-105% UFL: NLT 95% of stated IU LFL: NMT 120 % of stated IU*	**206,182 IU/ml**	**205,268 IU/ml**	**201,465 IU/ml**	**204,480 IU/ml**	**204.835 IU/ml**	***Not available (*)***	**207,986 IU/ml**	
**Colistin Sulphate Assay** *(after reconstitution)*	10 mg/ml *Assay precision fiducial limits: 95-105% UFL: NLT 97% of stated mg LFL: NMT 110 % of stated mg*	**10.2 mg/ml**	**9.8 mg/ml**	**9.9 mg/ml**	**9.8 mg/ml**	**9.8 mg/ml**	***Not available (*)***	**10.1 mg/ml**	
**Tobramycin Assay** *(after reconstitution)*	8 mg/ml *7.2 mg/g –9.6 mg/g*	**8.68 mg/ml**	**8.77 mg/ml**	**8.77 mg/ml**	**8.57 mg/ml**	**8.79 mg/ml**	**8.72 mg/ml**	**8.44 mg/ml**	
**Microbiological quality** *(after reconstitution)*	Complies TAMC: 10^2 CFU/g TYMC: 10^1 CFU/g *E.coli*: Absence/g	**complies**						**complies**	
**PET** *(after reconstitution)*	Complies USP requirements							**complies**	
**Date completed**		**05/12/17**	**14/12/17**	**20/12/17**	**22/12/17**	**16/01/18**	**16/01/18**	**07/03/18**	

Comments: ***The tests with (*) have not been performed.***

Checked By:________________________ (Sign and Date) **Authorised By QA Manager:**________________________ (Sign and Date)

7. SDD Suspension NF 25°C- 60%RH

VERITA PHARMA	Stability Report	Page 1 of 1

**Product Name: SDD Suspension NF**	**Product Code: N/A**	**Company Name: The George Institute**
**Dosage Form: suspension**	**Batch No: 1810005**	**Stability Study: SP-0006-01**
**Packaging: 240 ml amber plastic bottle with adapter and child resistant cap**	**Storage Temperature: 25°C ± 2°C**	**Humidity: 65 % RH ± 5 %RH**

**Time points**		**Initial**	**1 W**	**2 W**	**4 W**
**Due date**			29/01/19	01/02/19	18/02/19
**Test**	**Specifications**				
**Appearance**	After shaking, a yellow liquid	complies	complies	complies	complies
**pH**	4.0 – 5.0	4.58	4.50	4.59	4.72
**Nystatin Assay**	200,000 IU/ml<br>*Assay precision fiducial limits: 95-105%*<br>*UFL: NLT 95% of stated IU*<br>*LFL: NMT 120 % of stated IU*	204,141 IU/ml	199,838 IU/ml	200,830 IU/ml	202,077 IU/ml
**Colistin Sulphate Assay**	10 mg/ml<br>*Assay precision fiducial limits: 95-105%*<br>*UFL: NLT 97% of stated mg*<br>*LFL: NMT 110 % of stated mg*	10.4 mg/ml	10.0 mg/ml	9.7 mg/ml	9.7 mg/ml
**Tobramycin Assay**	8 mg/ml<br>*7.2 mg/g –9.6 mg/g*	7.81 mg/ml	8.53 mg/ml	7.97 mg/ml	8.00 mg/ml
**Microbiological quality**	Complies<br>TAMC: 10^2 CFU/g<br>TYMC: 10^1 CFU/g<br>*E.coli:* Absence/g	complies			complies
**PET**	Complies				complies
**Date completed**		08/11/2019	14/02/2019	20/02/2019	03/04/2019

Comments: ____

Checked By:________________________ (Sign and Date) **Authorised By QA Manager:**_________________________ (Sign and Date)

© Copyright - Verita Pharma
Information contained herein this document is the property of Verita Pharma - unauthorised disclosure to the public is prohibited.

8. SyrSpend SF pH4 Dry – 30% reduction in SDD Powder for Suspension Formulation

VERITA PHARMA

Syrspend SF pH4 Dry — 30% reduction in SDD Powder for Suspension Formulation

The current SDD Powder for Suspension contains 13.65g of Syrspend SF pH4 Dry (Syrspend SF), per bottle. Some sites currently part of the SuDDICU Clinical Trial, are finding some difficulty when reconstituting and using the suspension due to the thickness of the reconstituted product. The proposal is to reduce the quantity of Syrspend SF by 30% to 9.56g per bottle.

Syrspend SF is a starch based, preservative free suspending vehicle. The Syrspend SF contains; Modified Food

Starch (viscosity agent), Sucralose (sweetener) and a citrate/citric acid (buffer). The material allows the Active Pharmaceutical Ingredients (API's) to stay suspended and easy to (re)homogenise and is practically inert for chemical actions.

There are 3 API's in the SDD Suspension; Colistin Sulphate and Tobramycin Sulphate which are soluble in water and therefore dissolved in the suspension, and Nystatin which is suspended in the mixture and rehomogenised after shaking. The inert nature of the Syrspend SF means there should be no chemical interaction and therefore the efficacy of the drug remains the same. The microbiological stability of the product is maintained by the addition of Potassium Sorbate and Citric Acid to the powder blend. Both of these materials are dissolved in the water and the concentration of these materials will not change with the 30% reduction of Syrspend SF.

The reduction of the Syrspend SF quantity according to the supplier's procedure for use, falls within the recommended range of Water / Syrspend SF ratio.

On review with a quality consultant, Cathrine Dahlgren from CNQuality, it was concluded that the Syrspend SF reduction would not influence the activity of the 3 API's or the chemical and microbiological stability.

Based on the information reviewed it was determined that the current stability studies completed on the SDD Powder for Suspension and SDD Suspension (reconstituted) would be valid for the new formulation with a 30% reduction Syrspend SF. We are required to and will complete concurrent stability studies on the new formulation. The protocols have been written and are being approved.

Regards,

24. 09. 2018

Martina Bachmaier

General Manager

VERITA PHARMA PTY LTD ABN 89 604 520 532

UNIT 3/4 ENDEAVOUR ROAD CARINGBAH NSW 2229

PO BOX 2502 TAREN POINT NSW 2229

+612 8536 4130 | INFO@VERITAPHARMA.COM.AU I WWW.VERITAPHARMA.COM.AU

9. PICnIC Labels (Box, SDD oral paste, SDD suspension)

KEEP OUT OF REACH OF CHILDREN

FOR CLINICAL TRIAL USE ONLY

CHIEF INVESTIGATOR: Dr Nazima Pathan

SPONSOR: University of Cambridge & Cambridge University Hospitals NHS Trust
Addenbrookes Hospital, Hills Road, Cambridge
CB2 0QQ
Tel:01223 245151

PROTOCOL: PICnIC (REC 20/WM/0061)

BOX CONTAINS:

1 x Bottle of SDD Gastric Powder for Suspension (Total volume once reconstituted 210ml)

When reconstituted each 10ml contains: 2,000,000 units Nystatin, 100mg Colistin Sulfate, 80mg Tobramycin (as Sulfate)

20 x syringes of 500mg SDD Oral Paste

Each syringe contains: 10mg Colistin Sulfate, 10mg Tobramycin (as Sulfate) and 125,000 units Nystatin

Directions for use and reconstitution: Per the PICnIC protocol

Storage Conditions: Store at 2-8°C Do Not Freeze
In use: May be stored at up to 25°C for up to 5 days from date dispensed below.

FOR HOSPITAL USE:
Patient Trial Number:
Patient Full Name:
Date dispensed: ___/___/___
Use by: ___/___/___

Batch #:
Expiry:

Kit #

ITEM M-XXX-01

Sponsor:
University of Cambridge & Cambridge University Hospitals NHS Trust
Protocol: PICnIC (REC 20/WM/0061)

Keep out of reach of children

SDD ORAL PASTE 500mg

SINGLE USE ONLY

FOR CLINICAL TRIAL USE ONLY

Each syringe contains: 10mg Colistin Sulfate, 10mg Tobramycin (as Sulfate) and 125,000 units Nystatin

Batch: Store at 2-8°C
Expiry: In use storage: <25°C up to 5 days
Patient Trial No.:______________

ITEM M-XXX-01

Keep out of reach of children

SDD GASTRIC POWDER FOR SUSPENSION (200ml)

SHAKE WELL BEFORE USE
FOR CLINICAL TRIAL USE ONLY
Protocol: PICnIC (REC 20/WM/0061)

BATCH:
EXPIRY:

Patient Trial No.: __________
ONCE RECONSTITUTED DISCARD AFTER:
___/___/___

Sponsor:
University of Cambridge & Cambridge University Hospitals NHS Trust

WHEN RECONSTITUTED EACH 10ml CONTAINS:
2,000,000 units Nystatin, 100mg Colistin Sulfate, 80mg Tobramycin (as Sulfate)

Store at 2-8°C
For oral use as directed by PICnIC protocol
Once reconstituted, may be stored at room temperature (up to 25°C) for up to 5 days.

ITEM M-XXX-01
