# Supplementary material for: Use of selective gut decontamination in critically ill children: protocol for the Paediatric Intensive Care and Infection Control (PICnIC) pilot study
Source: BMJ Open. 2022 Mar 11;12(3):e061838. doi: 10.1136/bmjopen-2022-061838 (PMC8919465; doi:10.1136/bmjopen-2022-061838)
Supplement: Supplementary data [file bmjopen-2022-061838supp002.pdf]

We would like to ask your permission to take some samples from your child during their PICU stay to help improve our understanding of infection control

We are part of a study looking at how we can improve infection control procedures within the PICU. To help us understand if these measures work, we need to know what 'normal' looks like in the unit. To do this, we are looking at samples taken from children before, during and after the study.

This is the period (before/during/after) and we will be taking samples from children admitted between (date) and (date)

We would like to take samples from your child at regular timepoints throughout the admission. The samples won't hurt your child and we will make them as comfortable as possible. When we can, we will take them whilst carrying out routine care of your child.

We will take: nasopharyngeal (swab from nose and throat), stool samples or rectal swabs, and where clinically indicated urine and sputum samples and a swab of any wounds your child may have.

There is no direct benefit to your child, this work will help to improve treatment for children in the future. You do not have to agree to these samples and it will not affect your child's medical care. We will destroy any samples we have that are not needed by the doctors/nurses.

All samples are stored and analysed anonymously, it will not be possible to link them to your child. All research like this is reviewed by an independent group, called a Research Ethics Committee, who protect you and your child's interests. PICnIC was reviewed by West Midlands Black Country Health Research Authority who agree it is being conducted in a correct and appropriate matter.

If you would like more information, please speak to your child's bedside nurse, a member of the research team (name number) or the doctor in charge of the study (name number)

We are very grateful for your help - thank you

NHS National Institute for Health Research

Ecological Poster V1.2 06 October 2021

To be printed on local hospital headed paper

Paediatric Intensive Care and Infection Control (PICnIC) Study

Consent Form - Parent or Legal Guardian

Version 3.1, 25 September2020

To be completed by the Researcher:

Hospital name:	
PICnIC Study Number:	
Child's full name:	

To be completed by the Parent or Legal Guardian:

Once you have read and understood each statement – **if you agree, please write your initials in each box**

1.	I confirm that I have read and understand the Participant Information Sheet (version (please insert) dated(please insert)) for the above study. I have had the opportunity to consider the information, ask questions and have had these answered satisfactorily.	☐
2.	I understand that participation is voluntary, and that I am free to withdraw consent at any time, without giving any reason and without my child's medical care or legal rights being affected.	☐
3.	I agree for additional samples from my child to be taken, analysed, and stored as part of this research study.	☐
4.	I understand that relevant sections of my child's medical records and identifiable data collected during the study, held by the NHS, may be looked at by individuals from the Intensive Care National Audit & Research Centre (ICNARC) or regulatory authorities where it is relevant to my participation in this research.	☐
5.	I agree to complete a questionnaire about my views on conducting a research study.	☐
6.	I agree to be contacted for a telephone interview within the next 4 weeks. I understand that my contact details will be securely passed to the PICnIC study team at ICNARC and the University of Liverpool.	☐

Your signature:		Date:	
Your full name (PRINT):			
Researcher signature:		Date:	
Researcher full name (PRINT):			

Please sign and turn over to complete

Brown A, *et al. BMJ Open* 2022; 12:e061838. doi: 10.1136/bmjopen-2022-061838

Paediatric Intensive Care and Infection Control (PICnIC) Study

Parent or Legal Guardian contact information

To be completed by the Parent or Legal Guardian:

If you agree to be contacted for a telephone interview, please provide your details below:

**Telephone number:**	
**Mobile number:**	
**Email address:**	

1 copy for patient and parent/guardian; 1 copy for Investigator Site File; 1 copy to be kept with hospital notes

To be printed on local hospital headed paper

PICnIC

Paediatric Intensive Care and Infection Control (PICnIC) Study

Consent Form – Parent or Legal Guardian

Version 1.1, 10 September 2021

To be completed by the Researcher:

Hospital name:	
PICnIC Study Number:	
Child's full name:	

To be completed by the Parent or Legal Guardian:

Once you have read and understood each statement – **if you agree, please write your initials in each box**

1.	I confirm that I have read and understand the Participant Information Sheet (version (please insert) dated (please insert)) for the above study. I have had the opportunity to consider the information, ask questions and have had these answered satisfactorily.	☐
2.	I understand that participation is voluntary, and that I am free to withdraw consent at any time, without giving any reason and without my legal rights being affected.	☐
3.	I understand that relevant sections of my child's medical records and identifiable data collected during the study, held by the NHS, may be looked at by individuals from the Intensive Care National Audit & Research Centre (ICNARC) or regulatory authorities where it is relevant to my participation in this research.	☐
4.	I agree to be contacted for a telephone interview within the next two months. I understand that my contact details will be securely passed to the PICnIC study team at ICNARC and the University of Liverpool.	☐

Your signature:		Date:	
Your full name (PRINT):			
Researcher signature:		Date:	
Researcher full name (PRINT):			

Please sign and turn over to complete

Brown A, *et al. BMJ Open* 2022; 12:e061838. doi: 10.1136/bmjopen-2022-061838

Paediatric Intensive Care and Infection Control (PICnIC) Study

Parent or Legal Guardian contact information

To be completed by the Parent or Legal Guardian:

If you agree to be contacted for a telephone interview, please provide your details below:

**Telephone number:**	
**Mobile number:**	
**Email address:**	

1 copy for patient and parent/guardian; 1 copy for Investigator Site File; 1 copy to be kept with hospital notes

Brown A, *et al. BMJ Open* 2022; 12:e061838. doi: 10.1136/bmjopen-2022-061838
