# Supplementary material for: Use of selective gut decontamination in critically ill children: protocol for the Paediatric Intensive Care and Infection Control (PICnIC) pilot study
Source: BMJ Open. 2022 Mar 11;12(3):e061838. doi: 10.1136/bmjopen-2022-061838 (PMC8919465; doi:10.1136/bmjopen-2022-061838)
Supplement: Author's manuscript [file bmjopen-2022-061838.draft_revisions.pdf]

BMJ Open is committed to open peer review. As part of this commitment we make the peer review history of every article we publish publicly available.

When an article is published we post the peer reviewers' comments and the authors' responses online. We also post the versions of the paper that were used during peer review. These are the versions that the peer review comments apply to.

The versions of the paper that follow are the versions that were submitted during the peer review process. They are not the versions of record or the final published versions. They should not be cited or distributed as the published version of this manuscript.

BMJ Open is an open access journal and the full, final, typeset and author-corrected version of record of the manuscript is available on our site with no access controls, subscription charges or pay-per-view fees (http://bmjopen.bmj.com).

If you have any questions on BMJ Open's open peer review process please email info.bmjopen@bmj.com

BMJ Open

The use of selective gut decontamination in critically ill children: Paediatric Care and Infection Control (PICnIC): A protocol of a pilot cluster randomised trial

Journal:	*BMJ Open*
Manuscript ID	bmjopen-2022-061838
Article Type:	Protocol
Date Submitted by the Author:	09-Feb-2022
Complete List of Authors:	Brown, Alanna; Intensive Care National Audit and Research Centre<br>Ferrando, Paloma; Intensive Care National Audit and Research Centre<br>Popa, Mariana; University of Liverpool, Institute of Life and Human Sciences<br>de la Fuente, Gema Milla; Intensive Care National Audit and Research Centre<br>Pappachan, John; Southampton Children's Hospital, PICU<br>Cuthbertson, Brian; Sunnybrook Health Sciences Centre, Department of Critical Care<br>Drikite, Laura; Intensive Care National Audit and Research Centre, Clinical Trials Unit<br>Feltbower, Richard; University of Leeds<br>Gouliouris, Theodore; Cambridge University, Department of Medicine<br>Sale, Isobel; PPI representative<br>Shulman, Robert; University College London Hospitals NHS Foundation Trust, Department of Pharmacy<br>Tume, Lyvonne; University of Salford, School of Health and Society<br>Myburgh, John; The George Institute for Global Health<br>Woolfall, Kerry; University of Liverpool<br>Harrison, David; ICNARC<br>Mouncey, Paul; ICNARC, Clinical Trials Unit<br>Rowan, Kathryn; ICNARC<br>Pathan, Nazima; University of Cambridge, Department of Paediatrics; Cambridge University Hospitals NHS Foundation Trust,
Keywords:	Infection control < INFECTIOUS DISEASES, Paediatric intensive & critical care < ANAESTHETICS, MICROBIOLOGY

SCHOLARONE™
Manuscripts

The use of selective gut decontamination in critically ill children: Paediatric Care and Infection Control (PICnIC): A protocol of a pilot cluster randomised trial

AUTHORS

Alanna Brown[1], Paloma Ferrando-Vivas[1], Mariana Popa[2], Gema Milla de la Fuente[1], John Pappachan[3], Brian Cuthbertson[4], Laura Drikite[1], Richard Feltbower[5], Theodore Gouliouris[6], Isobel Sale[7], Robert Shulman[8], Lyvonne N Tume[9], John Myburgh[10], Kerry Woolfall[2], David A Harrison[1], Paul R Mouncey[1], Kathryn Rowan[1], Nazima Pathan[11].

Affiliations

[1] Clinical Trials Unit, Intensive Care National Audit and Research Centre, London, UK

[2] Department of Public Health, University of Liverpool, Liverpool, UK

[3] Paediatric Intensive Care Unit, Southampton Children's Hospital, University of Southampton, Southampton, UK

[4] Department of Critical Care Medicine, Sunnybrook Health Sciences Center, Toronto, Canada.

[5] Division of Epidemiology, University of Leeds, Leeds, UK

[6] Clinical Microbiology and Public Health Laboratory, Addenbrooke's Hospital, Cambridge University Hospital NHS Foundation Trust, Cambridge, UK

[7] PPI representative, UK

[8] Department of Pharmacy, University College London Hospitals NHS Foundation Trust, London, UK

[9] Intensive Care Unit, Alder Hey Children's NHS Foundation Trust Liverpool, UK

[10] George Institute for Global Health, Faculty of Medicine, University of New South Wales, Sydney, NSW, Australia

[11] Department of Paediatrics, University of Cambridge, Addenbrooke's Hospital, Cambridge, UK

Corresponding author

Dr Nazima Pathan

University Lecturer and Consultant in Paediatric Intensive Care

Department of Paediatrics,

University of Cambridge,

Level 8, Addenbrookes Hospital, Hills Road

For peer review only - http://bmjopen.bmj.com/site/about/guidelines.xhtml

Cambridge CB2 0QQ

Tel: 020 7269 9277

Email: np409@medschl.cam.ac.uk

Keywords

Selective digestive tract decontamination, Invasive mechanical ventilation, randomised clinical trial, paediatric critical care

Word count: Abstract (286), Main manuscript (3772/4000)

For peer review only

For peer review only - http://bmjopen.bmj.com/site/about/guidelines.xhtml

ABSTRACT

Introduction

Healthcare-associated infections (HCAI) are a major cause of morbidity and mortality in critically ill children. In critically ill adults, there are data that suggest the use of Selective Decontamination of the Digestive tract (SDD), alongside standard infection control measures reduce mortality and the incidence of HCAIs. SDD-enhanced infection control has not been compared directly with standard infection prevention strategies in the Paediatric Intensive Care Unit (PICU) population. The aim of this pilot study is to determine the feasibility of conducting a multicentre cluster randomised controlled trial (cRCT) in critically ill children comparing SDD with standard infection control.

Methods and analysis

PICnIC is a parallel group pilot cRCT, with integrated mixed-methods study, comparing incorporation of SDD into infection control procedures to standard care. After a one week pre-trial ecology surveillance period, recruitment to the cRCT will run for a period of 18 weeks, comprising: (1) baseline control period (2) pre, mid and post-trial ecology surveillance periods and (3) intervention period. Six PICUs will begin with usual care in Period One, then will be randomised 1:1 by the trial statistician using computer-based randomisation, to either continue to deliver usual care or commence delivery of the intervention (SDD) in Period Two. Outcomes measures include parent and healthcare professionals' views on trial feasibility, adherence to the SDD intervention, estimation of recruitment rate and understanding of potential patient-centred primary and secondary outcome measures for the definitive trial. The planned recruitment for the cRCT is 324 participants.

Ethics and dissemination

The trial received favourable ethical opinion from West Midlands – Black Country Research Ethics Committee (reference: 20/WM/0061) and approval from the Health Research Authority (IRAS number: 239324). Results will be disseminated via publications in peer-reviewed medical journals.

Trial registration number

ISRCTN40310490

For peer review only - http://bmjopen.bmj.com/site/about/guidelines.xhtml

Strengths and limitation of this study

- The study will examine the processes that are important in a future clinical trial of SDD in the PICU setting.
- The study uses a GMP-certified commercial SDD preparation under licence from the George Institute, Australia (Verita Pharma Pty, Australia).
- The study will evaluate the perspective of parents and the views of stakeholders including caregivers and the multi-disciplinary team on the processes needed to undertake a trial of SDD in the PICU.
- The use of SDD-enhanced infection control requires it to be implemented unit-wide, along with the support of local microbiology, pharmacy and infection control teams for delivery and monitoring in sites randomised to implement it.

For peer review only

For peer review only - http://bmjopen.bmj.com/site/about/guidelines.xhtml

INTRODUCTION

In critically ill children, healthcare-associated infections (HCAIs) are a major cause of morbidity and mortality, with a reported prevalence of 7-14%.[1-5] HCAIs can develop either as a direct result of healthcare interventions such as medical or surgical treatment, or from being in contact with a healthcare setting. In the critical care setting, the high use of invasive devices such as endotracheal tubes, vascular and urinary catheters increase the risk of secondary infection by opportunistic organisms. In particular, respiratory HCAIs (ventilator associated pneumonia, VAP) may occur with spread of commensal and other organisms from oro-pharyngeal and upper gastrointestinal compartments into the lungs.[4,5]

Evidence from adult intensive care studies suggests that using Selective Decontamination of the Digestive tract (SDD) alongside standard infection control measures reduces mortality and ventilator-associated pneumonia (VAP).[6,7] It has been shown that the use of SDD influences the microbiological ecology of the unit, thereby reducing incidence of HCAIs in both exposed and non-exposed patients. Despite this, SDD has not been routinely adopted due to concerns that it may promote antimicrobial resistance.[6,8] Recent ecological studies conducted in adult intensive care have found that SDD was associated with a reduction in antibiotic utilisation;[9-13] two large cluster randomised controlled trials (cRCTs) have been recently undertaken to further evaluate the clinical effects of SDD in adult intensive care. One reported no change in the incidence of blood stream infections, but the incidence of ventilator acquired pneumonia and overall antimicrobial use have not yet been reported.[14] Another large-scale multicentre study using the same formulation as the PICNIC study has recently completed enrolment of critically ill adults in Australia, Canada and the UK.[15]

SDD has yet to be compared directly with modern infection control protocols within the paediatric intensive care unit (PICU) population. The only trial data suggest a reduction in incidence of VAP but not mortality, however the study was underpowered and the observed mortality was very low.[16] Therefore, a clinical trial comparing SDD with standard infection control methods is required. Given the paucity of data describing the use of SDD in PICU and to establish the appropriate safety and ecological monitoring protocols, it is first imperative to establish whether a large, multicentre trial is feasible.

The PICnIC pilot study is a feasibility study designed to determine whether it is possible to conduct a cRCT of SDD in critically ill children who are likely to be ventilated for >48 hours, and to explore and test the acceptability of key components of the study to healthcare professionals and families of patients.

For peer review only - http://bmjopen.bmj.com/site/about/guidelines.xhtml

METHODS

Aim

To determine whether it is feasible to conduct a multicentre trial in critically ill children comparing SDD with standard infection control procedures.

Objectives

- To test the ability to randomise PICUs to either control or intervention
- To test the willingness and ability of healthcare professionals to screen and recruit eligible children
- To estimate the recruitment rate of eligible children
- To test adherence to the SDD protocol (including tolerance and application of a standardised SDD paste and suspension in a paediatric population)
- To test the procedures for assessing and collecting selected clinical and ecological outcomes and for adverse event (AE) reporting
- To assess the generalisability of the study
- To explore parent and healthcare professional views on the acceptability of the proposed trial, including recruitment and consent procedures and patient centred outcomes

Design

External pilot, parallel group cRCT with integrated mixed-methods study. After a one-week pre-trial ecology surveillance period, recruitment to the cRCT will run for a period of 18 weeks, comprising: (1) baseline control period ('Period One' - weeks 2-9); (2) mid-trial ecology surveillance period (week 10); and (3) intervention period ('Period Two' - weeks 11-19). On completion of Period Two, an additional one-week post-trial ecology surveillance period will be carried out (Figure 1). Six PICUs with a diverse geographical/demographic population representative of national PICU activity and size, will be randomised 1:1 by the trial statistician using computer-based randomisation, to either continue to deliver usual care or commence delivery of the intervention (SDD) in Period Two (Figure 2).

Screening

Potentially eligible patients presenting to the participating unit will be screened against the inclusion/exclusion criteria by the local clinical team, supported by the site research team. Screening Logs will record the reason patients are eligible but are subsequently not enrolled.

For peer review only - http://bmjopen.bmj.com/site/about/guidelines.xhtml

Eligibility – Ecology Surveillance Periods

Inclusion criteria

- All patients admitted to the PICU, regardless of ventilation status, during any of the three ecological surveillance periods

Exclusion criteria

- None

Eligibility – Period One and Period Two

Inclusion Criteria

- >37 weeks corrected gestational age to <16 years
- Receiving invasive mechanical ventilation, expected to last at least 48 hours
- Expected to remain on invasive mechanical ventilation until the day after tomorrow (from time of screening)

Exclusion Criteria

- Known allergy, sensitivity or interaction to polymyxin E (colistin), tobramycin or nystatin
- Known to be pregnant
- Death perceived as imminent

Microbiology Sampling

Samples taken:

- Nasopharyngeal
- Stool/rectal swabs
- Urine (if clinically indicated)
- Sputum/secretions from the endotracheal tube (If clinically indicated)
- Wound swabs, if present (if clinically indicated)

During the designated ecology surveillance periods, samples will be taken on admission and then taken on a Friday, if the patient has not had samples taken in the previous 48 hours.

During Periods One and Two, samples will be taken on admission and then twice weekly until discharge. For patient stays of less than 7 days, samples should be taken on the day of discharge.

For peer review only - http://bmjopen.bmj.com/site/about/guidelines.xhtml

For peer review only

Trial Intervention

During the Ecology Surveillance periods, Period One and for sites randomised to usual care in Period Two, there is no intervention. Patients will receive all standard infection control measures (per the site's specific policies) but will receive no study specific intervention.

For sites randomised to the intervention in Period Two, it will form part of the standard infection control strategy in the participating PICU. In addition to usual care, an SDD-enhanced infection control regimen will be delivered to all eligible patients using a GMP-certified commercial SDD preparation under licence from The George Institute, Australia (Verita Pharma Pty, Australia). (Within Supplementary File 1, see Supplementary Table 1 for SDD administration details, Supplementary Table 2-3 for composition and characteristics of SDD, Supplementary Table 4-5 for SDD formulation, Supplementary Appendices 1 for GMP licence, Supplementary Appendices 2-8 for SDD stability data and Supplementary Appendices 9 for PICnIC labels). Dosing of the SDD suspension will be calculated according to age (Table 1):

For peer review only

Table 1: SDD suspension dosing

	**0 – 4 years**	**5 – 12 years**	**≥13 years**
**Polymyxin E (Colistin)**	25mg	50mg	100mg
**Tobramycin**	20mg	40mg	80mg
**Nystatin**	0.5 x 106 IU	1 x 106 IU	2 x 106 IU
	**2.5ml**	**5ml**	**10ml**

1. A six-hourly topical, application of a pea-sized (0.5g) SDD paste containing 2% polymyxin E (colistin), 2% tobramycin and 2% nystatin to the buccal mucosa and oropharynx; and

2. A six-hourly administration of SDD suspension administered via the most proximal feeding tube into the stomach containing polymyxin E (colistin), tobramycin and nystatin.

SSD preparations will be distributed by the manufacturer in temperature-controlled (2-8ºC), patient specific kits to the participating hospital pharmacies. Each kit contains a 5-day supply of SDD treatment which includes a bottle of SDD suspension powder for reconstitution and

For peer review only - http://bmjopen.bmj.com/site/about/guidelines.xhtml

20 x1ml syringes containing Oral Paste. When a kit is allocated to a patient and the SDD powder is reconstituted into suspension, the kit can be stored at room temperature (≤27ºC) for up to 5 days. SDD treatment should be started within 6 hours of the patient being identified as eligible and continue for a maximum of 30 days (treatment period).

Treatment will continue until the patient is extubated or no longer mechanically ventilated (in tracheostomised patients). Patients subsequently re-intubated (either during this PICU admission or readmission to PICU from another inpatient area) during the treatment period will restart the intervention. All other usual care will be provided at the discretion of the treating clinical team.

Consent procedures

Children who are eligible for PICnIC will often become so during a period of life-threatening illness. This is a stressful time for parents/guardians during which time there are ethical concerns both about the burden placed of trying to understand the trial and their ability to provide informed consent.[17]

Posters displayed throughout the PICU will explain ecology sampling procedures and invite parents/guardians to have further discussions with research staff. The SDD intervention will be delivered, in additional to standard infection control policies, to all eligible patients in sites randomised to the SDD intervention during Period Two. Research staff will not seek individual consent for this. This approach is in line with guidance from the Ottawa Statement on the ethical design and conduct of cRCT.[18,19] (See supplementary file 2 for poster and consent forms).

Consent will be required for:

- Any additional study-specific samples to be taken, stored, and analysed prior to being taken solely for the study and not as part of routine care.
- Identifiable data collected and processed for participation in the mixed methods aspects of the study (questionnaire and interview).
- Monitoring of medical records.

Consent will not be required for:

- Samples taken as part of routine care (e.g. admission samples).
- Anonymised data collection and processing from routine sources.
- Delivery of SDD. For PICUs randomised to the intervention during Period Two, all children meeting the eligibility criteria will receive SDD as the standard practice.

For peer review only - http://bmjopen.bmj.com/site/about/guidelines.xhtml

Children may be withdrawn from trial specific data collection by the request of parents who decline participation in the research. All data collected up to the point of withdrawal will be retained and included in the study analysis.

If the patient has died, the parents/guardians will be approached for consent for the monitoring of medical records and identifiable data collected, and for the interview aspect of the study once established whether it is appropriate to approach for consent.

Data collection

Detailed guidance for the collection of data will be provided in the trial-specific standard operating procedure. It will include:

- Demographics
- Date/time of commencing mechanical ventilation
- Date/time identified as eligible
- Antibiotic usage throughout admission (route, type, duration, frequency, dose)
- Date/time of final extubation
- Details of any HCAI (confirmed/presumed)
- Date/time of PICU discharge
- Date/time of hospital discharge
- SDD delivery (dose for age, date time first dose, dose per day, change of dose, protocol deviation and reason for deviation)

No identifiable participant data will be required by the ICNARC Clinical Trials Unit (CTU) and all participant data will be stored securely. ICNARC is registered under the Data Protection Act (1998), and all ICNARC CTU staff have undergone data protection and International Conference on Harmonisation (ICH) Good Clinical Practice (GCP) training.

Patients will be followed-up until discharge from a participating PICU. Data entered onto the secure trial database will undergo validation checks for completeness, accuracy, and consistency of data. Queries on incomplete, inconsistent, or non-adherent data will be sent to the relevant PICU team for resolution.

Routine linkage will be made for all patients with the Paediatric Intensive Care Audit Network (PICANet) through the PICANet ID and trial number to obtain:

- Baseline demographics and risk factors, including predicted risk of death

For peer review only - http://bmjopen.bmj.com/site/about/guidelines.xhtml

- Secondary outcomes of critical care and acute hospital mortality, organ support received

Safety monitoring

Adverse event (AE) reporting will follow the Health Research Authority (HRA) guidelines on safety reporting in studies which do not use Investigational Medicinal Products.

AEs will be recorded during Period Two, from enrolment until PICU discharge (including readmissions from other inpatient areas until 30 days from being identified as eligible). For patients receiving SDD intervention, only AEs deemed possibly, probably or definitely related to the trial intervention should be reported to ICNARC CTU, apart from NG tube blockages which should be reported even if not deemed related. For patients receiving usual care, only NG tube blockages will be reported.

The following events have been prespecified as potential AEs:

- NG tube blockage
- Choking on paste
- Allergic reaction to SDD

The following events are exempt for reporting as AEs or SAEs

- Deterioration of condition or death that is not related to the trial intervention
- AEs of other drugs not specified in the protocol.

Any event classified as 'severe', 'life-threatening' or 'fatal' in severity is considered a serious AE (SAE) and must be reported to ICNARC CTU. If the SAE is evaluated by the Chief Investigator or clinical member of the Trial Management Group (TMG) as a related and unexpected SAE, the ICNARC CTU will submit a report to the Research Ethics Committee (REC) within 15 calendar days.

Patient and Public Involvement

Caregivers of children admitted to PICU and a former patient were involved in prioritising the outcomes and designing the study protocol. Their input has continued with PPI representatives on the study oversight panel. A patient representative (former PICU patient) is a co-investigator and is an author of this manuscript.

OUTCOME MEASURES

For peer review only - http://bmjopen.bmj.com/site/about/guidelines.xhtml

As this is a pilot study, outcome measures for the study will be focused on assessing the feasibility of a larger scale definitive study.

The ability to randomise PICUs to either control or intervention will be assessed by the successful random assignment of three PICUs to the intervention without delay to subsequent phases of the trial.

The willingness and ability of healthcare professionals to screen and recruit eligible children will be assessed by the proportion of eligible children recorded on study screening logs successfully recruited to the pilot cRCT and the reported reasons for non-recruitment.

The potential recruitment rate for a future definitive cRCT trial of SDD-enhanced infection control in eligible children will be estimated by combining the proportion of eligible children recruited to the pilot cRCT with the size of the potentially eligible population (estimated from nesting the screening log data from participating PICUs within the national UK PICU data from PICANet).

Adherence to the SDD protocol will be assessed by the proportion of eligible children allocated to the intervention receiving (a) both elements and (b) each individual element of the SDD intervention, the number of days on which these elements were received relative to days eligible for the SDD intervention and the reported reasons for nonadherence.

Procedures for assessing and collecting selected clinical and ecological outcomes and for AE reporting will be assessed by the proportion of children with complete data for these outcomes including, for ecological outcomes, the proportion consenting to additional study specific sample collection.

Generalisability of the study results to all UK PICUs will be assessed by comparing baseline characteristics and outcomes for children recruited to the pilot cRCT with data from all potentially eligible children (receiving invasive mechanical ventilation for at least 3 calendar days) within participating PICUs and within all UK PICUs (from PICANet).

With the aim of understanding potential patient-centred primary and secondary outcome measures for the definitive cRCT, the following potential outcome measures will be reported:

- HCAI (confirmed/presumed) and microbiology results (if positive sample);
- Hospital mortality;
- PICU mortality;

For peer review only - http://bmjopen.bmj.com/site/about/guidelines.xhtml

- Mortality within 30 days post enrolment;
- Length of hospital stay;
- Length of stay in PICU;
- Duration of mechanical ventilation; and
- Organ support received.

STATISTICAL METHODS

Sample size

The PICnIC pilot study is set up to test the feasibility of the protocol to recruit eligible patients. Therefore, there is no primary outcome to be compared between the two groups and, hence, a usual power calculation to determine sample size is not appropriate. Instead, the sample size has been determined to be adequate to estimate critical parameters to be tested to a necessary degree of precision. Based on available data from PICANet, it is anticipated participating sites will see approximately 4.5 eligible children per week, therefore the anticipated recruitment rate is 3 children per PICU per week providing a total of approximately 324 children in 18 weeks, of which 90 would receive the intervention. The sample size of children receiving usual care would be sufficient to estimate a binary outcome present in 20% of the population with a precision of ±5%.

Analysis

An overview of the planned analyses for the PICnIC pilot study is provided below. The full statistical analysis plan will be lodged on the trial website ahead of database lock.

A CONSORT flow diagram will be used to summarise the number and percentage of children screened, recruited and followed-up. This will include the proportion of eligible children successfully recruited and the reported reasons for non-recruitment.

Recruitment to the pilot cRCT will be presented as a rate per site per week over the two recruitment periods, overall, by treatment group and by site. Potential reasons for variation in recruitment rates will be explored. The potential recruitment rate for a future definitive cRCT will be estimated by combining the proportion of eligible children recruited to the pilot cRCT with the size of the potentially eligible population (estimated from PICANet).

Baseline demographic and clinical data will be summarised overall and for each of the two treatment groups in each of the two time periods but not subjected to statistical testing.

For peer review only - http://bmjopen.bmj.com/site/about/guidelines.xhtml

The proportion of eligible children allocated to the intervention that received both elements and each individual element of the SDD intervention will be reported as well as the number of days on which these elements were received relative to days eligible for the SDD intervention. The reported reasons for nonadherence will be detailed.

Data completeness of clinical and ecological outcomes and for AE reporting will be summarised.

Patient characteristics for children recruited to the pilot cRCT will be compared with those for potentially eligible children within participating PICUs and within all UK PICUs. Potential patient-centred clinical outcome measures for the definitive trial will be estimated and reported (proportion or mean and standard deviation, and intracluster correlation). As a pilot cRCT, there will be no statistical testing for any of the summary measures. Comparisons between groups will be used to estimate the potential magnitude of the treatment effect; p values will not be calculated or quoted.[20] To account for cluster randomisation, we will use multilevel logistic or generalised linear regressions in the above analyses.

Mixed methods study

The mixed methods study will involve a questionnaire and interviews with parents / legal guardians of children involved in the pilot cRCT as well as focus groups, interviews and an online survey with healthcare professionals. These will be used to review and explore:

- parent views on
    - the acceptability of a definitive trial that includes the SDD intervention.
    - the acceptability of the recruitment and consent procedures for the definitive trial, including all proposed information materials.
    - important, relevant, patient-centred primary and secondary outcomes for a definitive trial.
- healthcare professionals' views on
    - the acceptability of implementation of the SDD intervention, recruitment and consent procedures.
    - the acceptability of collecting data to assess the selected clinical and ecological data.
    - the acceptability of the SDD intervention and to confirm interest in participation in a definitive trial in the wider PICU community.

Inclusion criteria

For peer review only - http://bmjopen.bmj.com/site/about/guidelines.xhtml

- Parents/legal guardians of children involved in the pilot cRCT, including those who withdraw from data collection
- Healthcare professionals (including doctors, nurses, physios, pharmacists) working in PICUs that participate in the pilot cRCT.

Exclusion criteria

- Parents/legal guardians who do not speak English

Parents/legal representative recruitment and consent

Healthcare professionals will seek consent from parents of recruited children, including those who withdraw from data collection, to complete a questionnaire or register interest in a telephone or online interview. Questionnaires (n=~100) will be completed after pilot trial recruitment discussions and interviews will be conducted by the UoL team within a month until information power[21] is reached (n=~15-25 based on previous studies)

Healthcare professionals' recruitment and consent

Healthcare professionals involved in the pilot cRCT will be invited via email to participate in a virtual focus group (n=2) or interview (≥10 depending on information power). An online survey will be distributed through UK PICU networks.

Data Analysis of Mixed-Methods study

Interviews and focus groups will be transcribed, checked and anonymised as the study progresses. QSR NVivo software will be used to assist in the organisation and indexing of qualitative data. Whilst thematic analysis[22,23] will draw on the Theoretical Framework of acceptability.[24,25] The focus will be modified to fit with the criterion of catalytic validity, whereby findings should be relevant to future research and practice (in particular, the design of, and information to inform decisions on the progression to, a definitive cRCT). Quantitative data from parent questionnaires and the online survey will be analysed using SPSS software, descriptive statistics and exact tests will be used, as appropriate. Data from each method will be analysed separately then synthesised through the use of constant comparative analysis.[26]

ETHICS AND DISSEMINATION

The PICnIC pilot study will be conducted in accordance with the approved Trial Protocol, ICH GCP guidelines, the Data Protection Act (2018), the Mental Capacity Act (2005), as well as the ICNARC CTU's research policies and procedures.

For peer review only - http://bmjopen.bmj.com/site/about/guidelines.xhtml

The study received favourable ethical opinion from West Midlands – Black Country Research Ethics Committee (Ref: 20/WM/0061) and approval from the HRA (IRAS number: 239324).

The final report, including a detailed description of the trial, results and recommendations for future policy and practice and future research, will be submitted to the National Institute of Health Research Health Technology Assessment (NIHR HTA) Programme. Articles will be prepared for publication in peer-reviewed scientific journals, as well as relevant professional journals.

Oversight

The TMG, led by the chief investigator, is responsible for the management of PICnIC. It meets regularly and includes the Investigators and ICNARC CTU trial team. PICnIC is managed by the ICNARC CTU in accordance with the Medical Research Council's Good Research Practice: Principles and Guidelines[27] which is based on the ICH guidelines on GCP[28] principles and the UK Department of Health's Policy Framework for Health and Social Care Research.[29]

A majority independent TSC has been established to monitor trial progress and includes PPI representatives, experienced clinicians and researchers/statisticians, in addition to the chief investigator and head of research at ICNARC. An independent DMEC, comprising experienced clinicians and statisticians, has been established to monitor patient recruitment and retention, adherence and safety.

Cambridge University Hospitals NHS Foundation Trust and The University of Cambridge is the trial sponsor. As the sponsor is an NHS organisation, NHS indemnity will apply for legal liability arising from the design, management and conduct of the research.

Ownership of the data sits with the sponsor with collaboration agreements in place to allow access to necessary partners on the grant. Once a final anonymised dataset is created at the end of the study, requests for access to data will be reviewed and approved by the TMG.

Trial Status

The paper presents protocol v4.1, dated 17 December 2021. At the time of submission, patient recruitment was ongoing. Recruitment commenced in September 2021 with recruitment planned to complete in February 2022. Follow up data collection will continue until the end of March 2022.

For peer review only - http://bmjopen.bmj.com/site/about/guidelines.xhtml

Funding statement

This work was supported by NIHR HTA Programme (reference: 16/152/01). The views expressed are those of the author(s) and not necessarily those of the NIHR or the Department of Health and Social Care nor the Sponsor. The funder and sponsor had no role in the writing of this article of the decision to submit the protocol for publication. Cambridge University Hospitals NHS Foundation Trust and The University of Cambridge is the sponsor.

Authors' contributions

NP is chief investigator. AB is trial manager. PFV, MP, BC, RF, TG, JM, JP, IS, RS, LNT, DAH, PRM, KR, KW are trial co-applicants and members of the Trial Management Group. LD and GMF supported management of the trial. All authors read and approved the final manuscript.

Competing interests statement

NP, JP, BC, RF, TG, RS, LNT, KW, DAH, PRM, KR, AB, PFV, GMF, LD, MP are funded by the UK NIHR HTA research programme

KR is the Director of NIHR Health and Social Care Delivery Research (HSDR) Programme.

NP and LNT is a member of the NIHR HTA research prioritisation panel and LNT is a member of the NIHR HTA funding panel.

JM is Chair of SuDDICU Australia Management Committee, Director of The George Institute for Global Health and Leadership Fellowship of National Health and Medical Research Council, Australia.

For peer review only - http://bmjopen.bmj.com/site/about/guidelines.xhtml

REFERENCES

1. Patrick SW, Kawai AT, Kleinman K, et al. Health care-associated infections among critically ill children in the US, 2007-2012. Pediatrics 2014;134(4):705-12. doi: 10.1542/peds.2014-0613
2. Field-Ridley A, Dharmar M, Steinhorn D, et al. ICU-Acquired Weakness Is Associated With Differences in Clinical Outcomes in Critically Ill Children. Pediatr Crit Care Med 2016;17(1):53-57. doi:10.1097/PCC.0000000000000538
3. Choong K, Al-Harbi S, Siu K, et al. Functional recovery following critical illness in children: the "wee-cover" pilot study. *Pediatr Crit Care Med* 2015;16(4):310-8. doi: 10.1097/pcc.0000000000000362
4. Heyland DK, Cook DJ, Griffith L, et al. The attributable morbidity and mortality of ventilator-associated pneumonia in the critically ill patient. The Canadian Critical Trials Group. *Am J Respir Crit Care Med* 1999;159(4 Pt 1):1249-56. doi: 10.1164/ajrccm.159.4.9807050
5. Bekaert M, Timsit J-F, Vansteelandt S, et al. Attributable Mortality of Ventilator-Associated Pneumonia. *American Journal of Respiratory and Critical Care Medicine* 2011;184(10):1133-39. doi: 10.1164/rccm.201105-0867OC
6. Francis J, Duncan E, Prior M, et al. Selective decontamination of the digestive tract in critically ill patients treated in intensive care units: A mixed-methods feasibility study (the SuDDICU study). *Health technology assessment (Winchester, England)* 2014;18:1-170. doi: 10.3310/hta18250
7. de Smet AMGA, Kluytmans JAJW, Cooper BS, et al. Decontamination of the Digestive Tract and Oropharynx in ICU Patients. *New England Journal of Medicine* 2009;360(1):20-31. doi: 10.1056/NEJMoa0800394
8. Canter RR, Harvey SE, Harrison DA, et al. Observational study of current use of selective decontamination of the digestive tract in UK critical care units. *Br J Anaesth* 2014;113(4):610-7. doi: 10.1093/bja/aeu108
9. Daneman N, Sarwar S, Fowler RA, et al. Effect of selective decontamination on antimicrobial resistance in intensive care units: a systematic review and meta-analysis. *Lancet Infect Dis* 2013;13(4):328-41. doi: 10.1016/s1473-3099(12)70322-5
10. Liberati A, D'Amico R, Pifferi, et al. Antibiotic prophylaxis to reduce respiratory tract infections and mortality in adults receiving intensive care. *Cochrane Database Syst Rev* 2004(1):Cd000022. doi: 10.1002/14651858.CD000022.pub2
11. Oostdijk EAN, Smet AMGAd, Blok HEM, et al. Ecological Effects of Selective Decontamination on Resistant Gram-negative Bacterial Colonization. *American Journal of Respiratory and Critical Care Medicine* 2010;181(5):452-57. doi: 10.1164/rccm.200908-1210OC

For peer review only - http://bmjopen.bmj.com/site/about/guidelines.xhtml

12. Plantinga NL, Bonten MJ. Selective decontamination and antibiotic resistance in ICUs. *Crit Care* 2015;19(1):259. doi: 10.1186/s13054-015-0967-9
13. Price RJ, Cuthbertson BH, collaboration obotS. Selective decontamination of the digestive tract and oropharynx: after 30 years of debate is the definitive answer in sight? *Current Opinion in Critical Care* 2016;22(2):161-66. doi: 10.1097/mcc.0000000000000281
14. Wittekamp BH, Plantinga NL, Cooper BS, et al. Decontamination Strategies and Bloodstream Infections With Antibiotic-Resistant Microorganisms in Ventilated Patients: A Randomized Clinical Trial. *JAMA* 2018;320(20):2087-98. doi: 10.1001/jama.2018.13765
15. Billot L, Cuthbertson BH, et al. Protocol summary and statistical analysis plan for the Selective Decontamination of the Digestive Tract in Intensive Care Unit Patients (SuDDICU) crossover, cluster randomised controlled trial. *Critical Care and Resuscitation* 2021
16. Petros A, Silvestri L, Booth R, et al. Selective decontamination of the digestive tract in critically ill children: systematic review and meta-analysis. Pediatr Crit Care Med 2013;14(1):89-97. doi: 10.1097/PCC.0b013e3182417871
17. Woolfall K, Young B, Frith L, et al. Doing challenging research studies in a patient-centred way: a qualitative study to inform a randomised controlled trial in the paediatric emergency care setting. *BMJ Open* 2014; 4(5). http://europepmc.org/abstract/MED/24833694
18. Weijer C, Grimshaw JM, Eccles MP, et al. The Ottawa Statement on the Ethical Design and Conduct of Cluster Randomized Trials. *PLoS Med* 2012;9(11):e1001346. doi: 10.1371/journal.pmed.1001346
19. Nix HP, Weijer C, Brehaut JC, et al. Informed consent in cluster randomised trials: a guide for the perplexed. *BMJ Open* 2021;11(9):e054213-e13. doi: 10.1136/bmjopen-2021-054213
20. Chan CL, Leyrat C, Eldridge SM. Quality of reporting of pilot and feasibility cluster randomised trials: a systematic review. *BMJ Open* 2017; 7(11). https://doi.org/10.1136/bmjopen-2017-016970
21. Malterud K, Siersma VD, Guassora AD. Sample Size in Qualitative Interview Studies: Guided by Information Power. *Qual Health Res* 2016;26(13):1753-60. doi: 10.1177/1049732315617444
22. Boyatzis R. Transforming Qualitative Information: Thematic Analysis and Code Development. 1998
23. Braun V, Clarke V. Using thematic analysis in psychology. *Qualitative Research in Psychology* 2006;3(2):77-101. doi: 10.1191/1478088706qp063oa

For peer review only - http://bmjopen.bmj.com/site/about/guidelines.xhtml

24. Deja E, Peters MJ, Khan I, et al. Establishing and augmenting views on the acceptability of a paediatric critical care randomised controlled trial (the FEVER trial): a mixed methods study. *BMJ Open* 2021;11(3):e041952-e52.

25. Sekhon M, Cartwright M, Francis JJ. Acceptability of healthcare interventions: an overview of reviews and development of a theoretical framework. *BMC Health Serv Res* 2017;17(1):88. doi: 10.1186/s12913-017-2031-8

26. Glaser BG. The Constant Comparative Method of Qualitative Analysis*. Social Problems 1965;12(4):436-45. doi: 10.2307/798843

27. Medical Research Council Good research practice: principles and guidelines, 2012. Available: https://mrc.ukri.org/publications/browse/good-research-practice-principles-and-guidelines/

28. Academic. *International conference on harmonisation of technical requirements for registration of pharmaceuticals for human use. guideline for good clinical practice*, 1996.

29. Health Research Authority. UK policy framework for health and social care research, 2017. Available: https://www.hra.nhs.uk/planning-and-improving-research/policies-standards-legislation/uk-policy-framework-health-social-care-research/

For peer review only - http://bmjopen.bmj.com/site/about/guidelines.xhtml

FOOTNOTES

Acknowledgments

Our thanks to the research and clinical teams at the participating sites, and the support of the Biomedical Research Centres at the sites: Cambridge University Hospitals NHS Foundation Trust, Birmingham Women and Children's Hospital (Birmingham), Bristol Royal Hospital for Children (Bristol), John Radcliffe Hospital (Oxford), Southampton Children's Hospital (Southampton), St. George's Hospital (London).

.

For peer review only

For peer review only - http://bmjopen.bmj.com/site/about/guidelines.xhtml

Figure legends

Figure 1. Trial Design

Figure 2. Trial Schema of eligibility, site randomisation, study intervention, ecology surveillance, embedded mixed-methods study, follow up.

For peer review only

For peer review only - http://bmjopen.bmj.com/site/about/guidelines.xhtml

Supplementary Files

Supplementary file 1 for protocol paper including:

Supplementary Table 1: SDD suspension dosing

Supplementary Table 2: Composition and Characteristics of SDD paste

Supplementary Table 3: Composition and Characteristics of SDD suspension

Supplementary Table 4: General Investigational drug product information (SDD paste)

Supplementary Table 5: General Investigational drug product information (SDD suspension)

Appendices 1: Verita Pharma most recent GMP Licence

Appendices 2: SDD Oral Paste 5°C R&D stability summary

Appendices 3: SDD Oral Paste 25°C-60%RH R&D stability

Appendices 4: SDD Powder for Suspension 5°C R&D stability

Appendices 5: SDD Powder for Suspension 25°C-60%RH R&D stability

Appendices 6: SDD Suspension 5°C R&D stability

Appendices 7: SDD Suspension NF 25°C-60%RH

Appendices 8: SyrSpend SF pH4 Dry – 30% reductio in SDD Powder for Suspension Formulation

Appendices 9: PICnIC Labels (Boz, SDD oral paste, SDD suspension)

Supplementary file 2

Ecological Poster_v1.2, 06 October 2021

PICnIC Consent Form Parent or Legal Guardian (non bereaved)_v3.1, 25 September 2021

PICnIC Consent Form Parent or Legal Guardian (bereaved)_v1.1, 10 September 2021

For peer review only

For peer review only - http://bmjopen.bmj.com/site/about/guidelines.xhtml

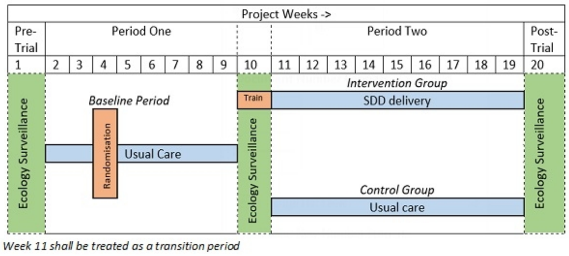

147x209mm (150 x 150 DPI)

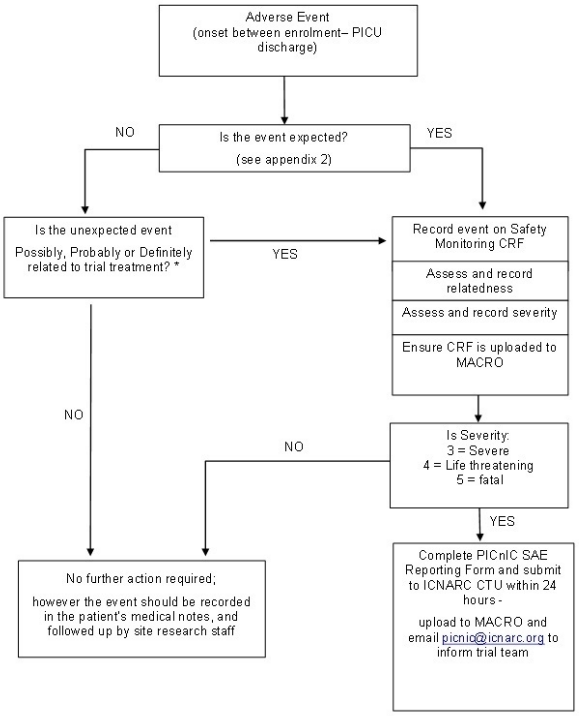

*If there is any uncertainty about whether the AE is associated with trial treatment, then it should be reported. Unexpected events should only be recorded for patients receiving SDD treatment .

147x209mm (150 x 150 DPI)

Supplementary file 1 for protocol paper:

The use of selective gut decontamination in critically ill children: Paediatric Care and Infection Control (PICnIC): A protocol of a pilot cluster randomised trial

How does SDD work?

SDD paste and SDD suspension are both formulations containing three non-absorbable antibiotics and antifungals: colistin (polymyxin E), tobramycin and nystatin. All three are licensed antibacterial and antifungal drugs, and are currently used in critically ill patients when indicated.

In SDD therapy, the drugs are used to eradicate the gastro-intestinal carriage of potentially pathogenic micro-organisms including Staphylococcus aureus, methicillin-resistant Staphylococcus aureus (MRSA), aerobic Gram-negative bacilli and yeasts.[1]

The drugs are non-absorbable, ensuring that their concentrations remain high enough in the mouth and into the colon to selectively eradicate the carriage of pathogenic micro-organisms, whilst not influencing the protective anaerobic flora, thereby decreasing colonisation resistance.[1]

Colistin is a multicomponent antibiotic. It is a mixture of several closely related decapeptides (polymyxin E). The main components are polymyxin E1 and E2. Colistin has an antimicrobial spectrum and mode of action similar to that of polymyxin B, but is slightly less active. It is bactericidal to most Gram-negative bacteria.[1]

Tobramycin sulphate is an aminoglycoside antibiotic with good aqueous solubility. It acts against many strains of Gram-negative bacteria, including pseudomonas aeruginosa.[1]

Colistin and tobramycin act in synergy against proteus and pseudomonas species, and offer the most potent anti-pseudomonal combination, with an effective clearance of pseudomonas from the gut. Both agents absorb endotoxin released by aerobic Gram-negative bacilli. This feature is important because endotoxin can be absorbed into the bloodstream from the gut of seriously ill patients. This contributes to fever, inflammatory activation, shock and organ failure.[1,2]

Emergence of resistance to colistin is rare. Although there are bacteria producing tobramycin-inactivating enzymes, colistin is thought to protect tobramycin from being destroyed by these enzymes. Tobramycin is intrinsically the most active aminoglycoside against pseudomonas, and is minimally inactivated by saliva and faeces.

In critically ill patients, the use of intravenous antimicrobials suppress the patient's commensal species in the gastrointestinal tract. This is associated with overgrowth of extended-spectrum beta-lactamase (ESBL)-producing aerobic Gram-negative bacilli in the gut. The action of enteral colistin and tobramycin against aerobic Gram-negative bacilli prevents the persistence of ESBL producing aerobic Gram-negative bacilli.[3]

Nystatin is a non-absorbable polyene drug with wide antifungal activity, especially against candida species. It significantly reduces fungal carriage and overall fungal infections and is less likely to promote the emergence of resistant candidal strains compared to other antifungal agents.[4] It also has advantages of low cost and absence of side effects.[4]

The most widely used SDD regimen to date uses amphotericin B in combination with polymyxin (colistin) and tobramycin. For PICnIC, nystatin is used in place of amphotericin B

For peer review only

due to difficulties sourcing amphotericin B. Nystatin, like amphotericin B is a non-absorbable polyene with wide antifungal activity.[4]

How is SDD administered?
Every six hours a 'pea-sized' amount (0.5 g) of SDD paste will be topically applied to the buccal mucosa and oropharynx and an SDD suspension is administered to the gastrointestinal tract via the most proximal feeding tube, with dosing according to age (Supplementary Table 1):

Supplementary Table 1: SDD suspension dosing

	0 – 4 years	5 – 12 years	≥13 years
**Polymyxin E (Colistin)**	25mg	50mg	100mg
**Tobramycin**	20mg	40mg	80mg
**Nystatin**	0.5 x 10^6 IU	1 x 10^6 IU	2 x 10^6 IU
	**2.5ml**	**5ml**	**10ml**

Similarity to other compounds
The most widely used SDD regimen to date is a combination of polymyxin E (colistin), tobramycin and amphotericin B applied as an oral paste and suspension to treat both the throat and gut, respectively.

For PICNIC, nystatin will be used in place of amphotericin B due to difficulties sourcing amphotericin B. Nystatin, like amphotericin B is a non-absorbable polyene with wide antifungal activity.[5] Nystatin may prevent the emergence of resistant fungal strains such as candida species and has advantages such as its low cost and absence of side effects.

Chemistry, Manufacturing and controls.
The SDD paste and suspension has been manufactured in accordance with Good Laboratory Practice (GLP) and Good Manufacturing Practice (GMP) for clinical studies (See Appendix 1 for Verita Pharma GMP licence).

Development plan
An unpublished, inception cohort pilot study testing inclusion criteria and examining process measures and outcomes was conducted in an adult population by collaborators at The George Institute and informed the development of the PICnIC protocol. The outcomes of this study will inform the development of a full trial within paediatrics.

Physical, Chemical and pharmaceutical properties and formulation.
Study Drugs.
The active pharmaceutical ingredients of both the SSD paste (Supplementary Table 2) and SDD suspension (Supplementary Table 3) are colistin sulphate, tobramycin sulphate and nystatin.

For peer review only

Supplementary Table 2: Composition and Characteristics of SDD paste.

Name	SDD paste
International nomenclature	The paste is a semi-solid dosage forms containing finely dispersed solids, have a stiff consistency, and is intended for topical application.<br>Active pharmaceutical ingredients:<br>Colistin sulphate<br>Tobramycin (as sulphate)<br>Nystatin
Sponsor name	The George Institute for Global Health
Chemical abstract service number	Colistin : 1264-72-8<br>Tobramycin: 49842-07-1<br>Nystatin: 1400-61-9
Chemical structure	Colistin Sulphate 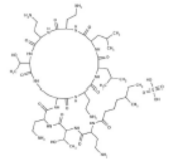<br>Tobramycin 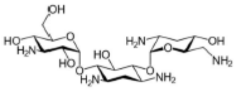<br>Nystatin 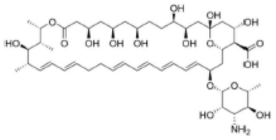
Molecular formula	Colistin : $2(C_{52}H_{98}N_{16}O_{13}).5(H_2SO_4)$<br>Tobramycin: $C_{18}H_{37}N_5O_9$<br>Nystatin: $C_{47}H_{75}NO_{17}$
Molecular weight	Colistin: 2801 g/mol<br>Tobramycin: 467.5 g/mol<br>Nystatin: 926.1 g/mol
Description	SDD paste is a mixture of antimicrobial powders – colistin sulphate, tobramycin sulphate and nystatin- with mineral oil light, petrolatum white and methocel E4M premium in an oral syringe.
Odour	Not applicable
Solubility	Not applicable
Properties	A smooth, yellow paste of uniform consistency

only

Supplementary Table 3: Composition and characteristics of SDD suspension.

Name	SDD suspension
International nomenclature	The suspensions is liquid preparations containing drug substance(s) and consist of solid particles dispersed throughout a liquid phase in which the particles are present in excess of the solubility. Some suspensions are prepared and ready for use, while others are solid mixtures intended for constitution before use with an appropriate vehicle.
Sponsor name	As per SDD paste
Chemical abstract service number	As per SDD paste
Structure	As per SDD paste
Molecular formula	As per SDD paste
Molecular weight	As per SDD paste
Description	SDD powder for suspension is a blend of antimicrobial powders- colistin sulphate, tobramycin sulphate and nystatin with a suspending agent (syrspend pH 4 dry), a preservative (potassium sorbate) and citric acid monohydrate in a bottle. Before use, each bottle is reconstituted with purified/distilled water to the required volume and then shaken.
Odour	Not applicable
Solubility	Part of the powder will be dissolved and part suspended
Properties	SDD suspension; after suspending: an opaque, straw coloured liquid

review only

Manufacture.
The SDD paste and SDD suspension are manufactured through a series of proprietary processing steps and performed in accordance with GLP/GMP under licence at:
Verita Pharma Pty LTD
Unit 3, 4 Endeavour Rd
Taren Point
NSW 2229
Australia

Analysis and characterisation of study drug.
The identity of each container within each batch of raw material destined for study drug is confirmed by full pharmacopoeia analysis by validated high-performance liquid chromatography (HPLC) and microbiology methodology (in accordance with annex 8 of Pharmaceutical Inspection Co-operation Scheme guide to GMP manufacturing).

Homogeneity and potency of each finished product batch of investigational product is confirmed via similar methodology.

Impurities will be assessed by Verita Pharma using HPLC and other analytical methods during on-going stability studies.

Stability
The manufacturer stability testing program is focused on determining allowable shelf-life and ensuring the product maintains compliance with the claims made on the SDD paste and SDD suspension labels when exposed to routine usage and storage conditions.

Stability testing uses HPLC and microbiology analyses to ensure that study drugs remains homogenous, with potency within specifications and exhibits normally expected physical

characteristics, when stored for defined durations, at the ranges of temperature and subjected to reasonably expected temperature excursions.

Investigational products

Formulation.

SDD paste

The clinical products are formulated by combining the ingredients shown in Supplementary Table 4 using a series of proprietary processing steps prior dispensing into 1ml oral syringes. SDD paste is formulated to contain per 0.5g paste: colistin sulphate 10 mg tobramycin 10 mg and nystatin 125,000 IU.

Supplementary Table 4: General Investigational drug product information.

Ingredient	Specification	Purpose	Conc. (per gram)
Colistin sulphate	BP	Active	20mg
Nystatin	Eur. Ph. 8th ed. (BP)	Active	0.25mu
Tobramycin (as sulphate)	USP	Active	20mg
Mineral oil light	USP	Excipient	50mg
Methocel e4m premium	USP	Excipient	177mg
Petrolatum white	USP	Excipient	686mg*

BP = British Pharmacopoeia provides quality standards for UK pharmaceutical substances and medicinal products
Eur.Ph.8th ed.= European Pharmacopoeia 8th Edition is Europe's scientific and legal benchmark for pharmacopoeia standards
USP = United States Pharmacopeia has established standards for manufacturing and supplying drugs worldwide
Active = an active component in a medicines final formulation
Excipient = an excipient is an inactive substance for the purpose of bulking-up drug formulations
Methocel e4m premium = a renewable raw material, are water soluble polymer derided from cellulose
Petrolatum white = semi-solid mixture of hydrocarbons.
* For 'petrolatum white' quantity added to batch is calculated to quantum satis

SDD suspension

The clinical products are formulated by combining the ingredients shown in Supplementary Table 5 using a series of proprietary processing steps and dispensing into defined aliquots within each polyethylene terephthalate (PET) bottle, for storage. When the product is required for the trial, each bottle of antimicrobial powder is reconstituted to the desired volume with purified/distilled water. The formulation includes a suspending agent (SyrSpend sf ph4) to ensure that when the bottle is standing, the active powder (Nystatin) is evenly dispersed after shaking between administrations to the patient, thereby preventing 'caking' on the bottom of the bottle. Due to the difficulties experienced at participating sites in achieving consistent reconstitution and the increased viscosity (thickening) of the suspension after 3 days, the formulation was modified in September 2018 to reduce the amount of SyrSpend by 30%. The removal of 30% suspending agent has no influence on product efficacy, chemical attributes or on the active components.

Supplementary Table 5: General investigational drug product information

Ingredient	Specification	Purpose	Concentration After reconstitution per ml
Colistin sulphate	BP	Active	10mg
Nystatin	Eur. Ph. 8th ed. (BP)	Active	0.20mu
Tobramycin (as sulphate)	USP	Active	8mg
Syrspend sf ph4	USP	Excipient	45.5mg
Citric acid monohydrate	BP	Excipient	2.86mg
Potassium sorbate	USP	Preservative	2.0mg

BP = British Pharmacopoeia provides quality standards for UK pharmaceutical substances and medicinal products
Eur.Ph.8th ed. = European Pharmacopoeia 8th Edition is Europe's scientific and legal benchmark for pharmacopoeia standards
USP = United States Pharmacopeia has established standards for manufacturing and supplying drugs worldwide
Active = an active component in a medicines final formulation
Excipient = an excipient is an inactive substance for the purpose of bulking-up drug formulations
Preservative = a natural or synthetic chemical that prevents decomposition by microbial growth or by undesirable chemical changes
Syrspend sf ph4 = is a ready-to-use, all-in-one suspending and sweetening oral liquid
Citric acid monohydrate = a tricarboxylic acid and maintains stability of active ingredients
Potassium sorbate = is the potassium salt of sorbic acid, commonly used preservative in the conservation of liquid pharmaceutical preparations.

Final dosage form and presentation
SDD paste (0.5g) is supplied in 1ml BD oral syringe and is stored at 2-8°C. During patient use the paste can be stored in a temperature-controlled room of less than 25°C for up to one week. SDD suspension is supplied in antimicrobial powder form in a sealed PET bottle with a syringe filling adaptor under a child resistant screwcap and is stored at 2-8°C. After reconstitution of the powder, the suspension can be stored in a temperature-controlled room of less than 25°C for up to one week.

Container and packaging
Each SDD study drug kit contains, twenty 1ml BD oral syringes SDD paste, a single sealed PET bottle of SDD suspension (powder form) and a syringe filling adaptor and shipped under refrigerated/cold chain 2-8˚C (35°–46°F) conditions to the clinical trial site.

Storage and handling
The SDD study drug kits are to be stored at 2–8°C (35°–46°F) in a secure area. When SDD powder is reconstituted into suspension the drug kit (paste and suspension) can be stored in a temperature controlled room of less than 25°C for up to 1 week. Each pre-filled syringe is to be discarded after use (i.e. single use only). The PET bottle is to be returned to the SDD study drug kit after each dose. Hospitals will need to provide their own 10ml oral/enteral syringe to access and measure each dose from the PET bottle (which contains a syringe filling adaptor).

Shelf life
There is no information in the literature regarding stability testing of drugs containing colistin sulphate, tobramycin sulphate and nystatin as this is a new formulation. In reference literature, data concerning stability testing of drugs containing polymyxin/tobramycin/amphotericin - the most widely used SDD regimen to date – is limited.

review only

Stability determinations/concentrations have therefore been assessed by HPLC and microbiology methods. Test methods have been developed and validated, and real-time ageing tests conducted to define safe storage parameters, including reasonably expected excursions.

The stability program for the SDD paste and SDD suspension is continually ongoing. The expiry date on the label will reflect the most recent shelf-life determination of the product contained within the syringe/bottle.
Current stability testing has demonstrated that:

1. SDD paste is stable at 2-8°C for up to 12 months.
2. SDD suspension is stable at 2-8°C for up to 2 weeks.
3. SDD powder for suspension is stable at 2-8°C for up to 12 months.
4. SDD paste, SDD powder for suspension and SDD suspension can be stored below 25°C for short excursions up to one week. (There is stability evidence up to one month, but this is not appropriate for the suspension because it is ‘in use’ at this stage. Suspension should be used as per the protocol once reconstituted.)

Refer to Appendices 2-8 for current stability results for SDD paste, SDD powder and SDD suspension.

For peer review only

Reference list

1. van Saene HKF, Silvestri L, de la Cal MA, Gullo A, editors. Infection Control in the Intensive Care Unit [Internet]. Milano: Springer Milan; 2012 [cited 2018 Oct 26]. Available from: http://link.springer.com/10.1007/978-88-470-1601-9
2. Pathan N, Burmester M, Adamovic T, Berk M, Ng KW, Betts H, et al. Intestinal injury and endotoxemia in children undergoing surgery for congenital heart disease. Am J Respir Crit Care Med [Internet]. 2011;184(11):1261–9. Available from: http://www.ncbi.nlm.nih.gov/pubmed/21868501
3. van der Spoel HI, Gerritsen RT. SDD for the Prevention and Control of Outbreaks. Selective Digestive Tract Decontamination in Intensive Care Medicine: a Practical Guide to Controlling Infection [Internet]. Milano: Springer Milan; 2008 [cited 2018 Oct 26]. p. 141–54. Available from: http://link.springer.com/10.1007/978-88-470-0653-9_11
4. Normand S, François B, Dardé M-L, Bouteille B, Bonnivard M, Preux P-M, et al. Oral nystatin prophylaxis of Candida spp. colonization in ventilated critically ill patients. Intensive Care Med [Internet]. 2005 Nov 30 [cited 2018 Oct 26];31(11):1508–13. Available from: http://www.ncbi.nlm.nih.gov/pubmed/16195905
5. Ruza F, Alvarado F, Herruzo R, Delgado MA, García S, Dorao P, et al. Prevention of nosocomial infection in a pediatric intensive care unit (PICU) through the use of selective digestive decontamination. Eur J Epidemiol [Internet]. 1998 Oct [cited 2018 Oct 25];14(7):719–27. Available from: http://www.ncbi.nlm.nih.gov/pubmed/9849834

For peer review only

Appendices

1. Verita Pharma most recent GMP Licence-MI-2016-LI-07350, dated 23 July 2020

Australian Government
Department of Health
Therapeutic Goods Administration

Licence to Manufacture Therapeutic Goods – Part 1

Licence Number:
MI-2016-LI-07350-1

Granted to:
Verita Pharma Pty Ltd
ABN: 89 604 520 532

Manufacturing Site Address:
Lot 8, 10 and 11 (SP92206), 2 Endeavour Road
Caringbah NSW 2229

The manufacturer above is hereby authorised under Section 38 of the *Therapeutic Goods Act 1989* to carry out the following steps in the manufacture of therapeutic goods at the manufacturing site address specified above.

Manufacturing Type	Sterility	Dosage Form	Product Category	Manufacturing Step
Medicine manufacture	Non Sterile	Semi Solids	Therapeutic Goods for Clinical Trials	Finished Product Manufacture
Medicine manufacture	Non Sterile	Semi Solids	Registered Therapeutic Good	Finished Product Manufacture
Medicine manufacture	Non Sterile	Powders Group	Therapeutic Goods for Clinical Trials	Finished Product Manufacture
Medicine manufacture	Non Sterile	Powders Group	Registered Therapeutic Good	Finished Product Manufacture
Medicine manufacture	Non Sterile	Oral Liquid, suspension	Therapeutic Goods for Clinical Trials	Finished Product Manufacture
Medicine manufacture	Non Sterile	Oil	Therapeutic Goods for Clinical Trials	Finished Product Manufacture
Medicine manufacture	Non Sterile	Spray	Therapeutic Goods for Clinical Trials	Finished Product Manufacture
Medicine manufacture	Sterile & Non Sterile	All Dosage Forms	Not Applicable	Testing chemical and physical

This Licence is the property of the Therapeutic Goods Administration and must be returned or destroyed upon demand.
This Licence remains valid until otherwise suspended or revoked by the Therapeutic Goods Administration.
The status of an Australian Licence may be viewed at https://www.ebs.tga.gov.au/

PO Box 100 Woden ACT 2606 ABN 40 939 406 804
Phone: 1800 020 653 Fax: 02 6203 1605 Email: info@tga.gov.au www.tga.gov.au

TGA Health Safety Regulation

Page 1 of 2

Australian Government
Department of Health
Therapeutic Goods Administration

Licence to Manufacture Therapeutic Goods – Part 1

Licence Number:
MI-2016-LI-07350-1

Manufacturing Type	Sterility	Dosage Form	Product Category	Manufacturing Step
Medicine manufacture	Sterile	Injections	Compounded Medicine	Sterile Finished Product Manufacture
Medicine manufacture	Sterile	Injections	Therapeutic Goods for Clinical Trials	Sterile Finished Product Manufacture

This licence is subject to the requirements of the *Therapeutic Goods Act1989*, and its Regulations.

Section 40(4) of the *Therapeutic Goods Act 1989* and Regulation 19, 20, 21 and 22 of the Therapeutic Goods Regulations 1990 impose various statutory conditions on all licences to manufacture therapeutic goods.

In addition to that, the specific conditions mentioned in Part 2 of this licence have been imposed under Section 40(1) or 40(2) of the *Therapeutic Goods Act 1989*.

Originally Granted: **16 May 2017** Date Revised: **23 July 2020**

This Licence is the property of the Therapeutic Goods Administration and must be returned or destroyed upon demand.
This Licence remains valid until otherwise suspended or revoked by the Therapeutic Goods Administration.
The status of an Australian Licence may be viewed at https://www.ebs.tga.gov.au/

PO Box 100 Woden ACT 2606 ABN 40 939 406 804
Phone: 1800 020 653 Fax: 02 6203 1605 Email: info@tga.gov.au www.tga.gov.au

TGA Health Safety Regulation

Page 2 of 2

2. SDD Oral Paste 5°C R&D Stability Summary

Form No: TF2402-01 VERITA PHARMA

R&D Stability Report

Product Name: **SDD Oral Paste**	Product Code: **FP002**	Company Name: **The George Institute**
Dosage Form: **Paste**	Batch No: **241117**	Stability Study: **SP-0001-03**
Packaging: **BD oral/enteral 1 ml syringe and tip cap**	Storage Temperature: **5°C ± 3°C**	Humidity: **N/A**

Time points		Initial	2 w	4 w	12 w	16 w	6 M	9 M	12M
**Due date**		27/11/17	11/12/17	08/01/18	19/02/18	19/03/18	27/05/18	27/08/18	27/11/18
**Test**	**Specifications**								
**Appearance**	Light yellow paste	complies	complies	complies	complies	complies	complies		complies
**Viscosity**	TBD	128,000 cps	157,000 cps	158,000 cps	150,333 cps	198,000 cps	198,000 cps		180,000 cps
**Nystatin Assay**	250,000 IU/g *Assay precision fiducial limits: 96-106%* *UFL: NLT 90% of stated IU* *LFL: NMT 115 % of stated IU*	243,621 IU/g	*Not available (*)*	242,806 IU/g	243,996 IU/g	244,270 IU/g	249,698 IU/g		245,342 IU/g
**Colistin Sulphate Assay**	20 mg/g *Assay precision fiducial limits: 96-106%* *UFL: NLT 97% of stated mg* *LFL: NMT 110 % of stated mg*	20.1 mg/g	*Not available (*)*	19.5 mg/g	19.6 mg/g	19.7 mg/g	19.9 mg/g	Cancelled	20.0 mg/g
**Tobramycin Assay**	20 mg/g *16 mg/g – 24 mg/g*	20.24 mg/g	21.0 mg/g	20.4 mg/g	20.56 mg/g	21.20 mg/g	21.65 mg/g		20.20 mg/g
**Microbiological quality**	Complies TAMC: 10^2 CFU/g TYMC: 10^1 CFU/g *S aureus: Absence/g* *P aeruginosa: Absence/g*	Complies			complies	complies			complies
**PET**	Complies								Complies USP
**Date completed**		19/12/17	28/12/17	17/01/18	08/03/18	10/04/18	14/06/18		05/04/2019

Comments: *The tests with (*) have not been performed.*

Checked By:________________________ (Sign and Date) **Authorised By QA Manager:**__________________________ (Sign and Date)

ew only

3. SDD Oral Paste 25°C-60%RH R&D Stability

Form No: TF2402-01

R&D Stability Report

Product Name: **SDD Oral Paste**	Product Code: **FP002**	Company Name: **The George Institute**
Dosage Form: **Paste**	Batch No: **241117**	Stability Study: **SP-0001-03**
Packaging: **BD oral/enteral 1 ml syringe and tip cap**	Storage Temperature: **25°C ± 2°C**	Humidity: **60 %RH ± 5% RH**

Time points		Initial	8 weeks	12 weeks					
**Due date**		**27/11/17**	**22/01/18**	**19/02/18**					
**Test**	**Specifications**								
**Appearance**	Light yellow paste	**complies**	**complies**	**complies**					
**Viscosity**	TBD	**128,000 cps**	**124,000 cps**	**123,000 cps**					
**Nystatin Assay**	250,000 IU/g<br>*Assay precision fiducial limits: 96-105%*<br>*UFL: NLT 90% of stated IU*<br>*LFL: NMT 115 % of stated IU*	**243,621 UI/g**	**241.968 UI/g**	**241.161 UI/g**					
**Colistin Sulphate Assay**	20 mg/g<br>*Assay precision fiducial limits: 96-105%*<br>*UFL: NLT 97% of stated mg*<br>*LFL: NMT 110 % of stated mg*	**20.1 mg/g**	**19.8 mg/g**	**19.7 mg/g**					
**Tobramycin Assay**	20 mg/g<br>*16 mg/g – 24 mg/g*	**20.24 mg/g**	**20.40 mg/g**	**20.87 mg/g**					
**Microbiological quality**	Complies<br>TAMC: 10^3 CFU/g<br>TYMC: 10^1 CFU/g<br>*S aureus*: Absence/g<br>*P aeruginosa*: Absence/g	**Complies**		**Complies**					
**PET**	Complies USP requirements		**Complies**						
**Date completed**		**19/12/17**	**08/03/18**	**08/03/18**					

Comments: ______________________________

Checked By:______________________ (Sign and Date) **Authorised By QA Manager:**________________________ (Sign and Date)

only

4. SDD Powder for Suspension 5°C R&D Stability

Form No: TF2402-01

VERITA PHARMA

R&D Stability Report

Product Name: **SDD Powder for Suspension**	Product Code: **FP001**	Company Name: **The George Institute**
Dosage Form: **Powder**	Batch No: **Int001071117**	Stability Study: **SP-0002-05**
Packaging: **Amber Plastic bottle with Child Resistant Cap**	Storage Temperature: **5°C ± 3°C**	Humidity: **N/A**

Time points		Initial	2 w	4 w	9 w	12 w	18 w	6 M	9 M	12 M
Due date		15/11/17	29/11/17	13/12/17	17/01/18	07/02/18	21/03/18	15/05/18	15/08/18	15/11/18
Test	Specifications									
Appearance	pale yellow powder	complies	complies	complies	complies	complies	complies	complies	Cancelled	complies
Loss on Drying	TBD	5.63 %	4.98 %	6.47 %	5.19 %	5.72 %	5.14 %	6.52 %		5.75 %
pH *(after reconstitution)*	4.0 – 5.0	4.49	4.71	4.63	4.58	4.45	4.32	4.54		4.53
Nystatin Assay *(after reconstitution)*	200,000 IU/ml *Assay precision fiducial limits: 95-105% UFL: NLT 95% of stated IU LFL: NMT 120 % of stated IU*	206,182 IU/ml	200,288 IU/ml	*Not available (*)*	205.602 IU/ml	203,390 IU/ml	200,970 IU/ml	205,321 IU/ml		202,614 IU/ml
Colistin Sulphate Assay *(after reconstitution)*	10 mg/ml *Assay precision fiducial limits: 95-105% UFL: NLT 97% of stated mg LFL: NMT 110 % of stated mg*	10.2 mg/ml	9.8 mg/ml	*Not available* (*)	9.5 mg/ml	9.9 mg/ml	9.9 mg/ml	10.2 mg/ml		10.1 mg/ml
Tobramycin Assay *(after reconstitution)*	8 mg/ml *7.2 mg/g –9.6 mg/g*	8.73 mg/ml	8.87 mg/ml	8.84mg/ml	8.66 mg/ml	8.85 mg/ml	8.18 mg/ml	8.75 mg/ml		8.09 mg/ml
Microbiological quality *(after reconstitution)*	Complies TAMC: 10^2 CFU/g TYMC: 10^1 CFU/g *E.coli*: Absence/g	complies				complies	complies			complies
PET *(after reconstitution)*	Complies									complies
Date completed		05/12/17	22/12/17	02/01/18	19/01/18	08/03/18	12/04/18	13/06/18		29/01/2019

Comments: ***The tests with (*) have not been performed.***

Checked By:________________________ (Sign and Date) **Authorised By QA Manager:**__________________________ (Sign and Date)

5. SDD Powder for Suspension 25°C-60%RH R&D Stability

Form No: TF2402-01

VERITA PHARMA

R&D Stability Report

Product Name: **SDD Powder for Suspension**	Product Code: **FP001**	Company Name: **The George Institute**
Dosage Form: **Powder**	Batch No: **Int001071117**	Stability Study: **SP-0002-05**
Packaging: **Amber Plastic bottle with Child Resistant Cap**	Storage Temperature: **25°C ± 2°C**	Humidity: **60% RH**

Time points		Initial	8 w	12 w					
Due date		15/11/17	10/01/18	07/02/18					
Test	Specifications								
Appearance	pale yellow powder	complies	complies	complies					
Loss on Drying	TBD	5.63	5.72	5.29					
pH *(after reconstitution)*	4.0 – 5.0	4.49	4.64	4.68					
Nystatin Assay *(after reconstitution)*	200,000 IU/ml *Assay precision fiducial limits: 95-105% UFL: NLT 95% of stated IU LFL: NMT 120 % of stated IU*	206,182 IU/ml	203,811 IU/ml	199,917 IU/ml					
Colistin Sulphate Assay *(after reconstitution)*	10 mg/ml *Assay precision fiducial limits: 95-105% UFL: NLT 97% of stated mg LFL: NMT 110 % of stated mg*	10.2 mg/ml	10.0 mg/ml	9.8 mg/ml					
Tobramycin Assay *(after reconstitution)*	8 mg/ml *7.2 mg/g –9.6 mg/g*	8.73 mg/ml	8.65 mg/ml	8.57 mg/ml					
Microbiological quality *(after reconstitution)*	Complies TAMC: 10^2 CFU/g TYMC: 10^1 CFU/g *E.coli*: Absence/g	complies		complies					
PET *(after reconstitution)*	Complies		Complies						
Date completed		05/12/17	08/03/18	08/03/18					

Comments: ______________________________

Checked By:______________________ (Sign and Date) **Authorised By QA Manager:**______________________ (Sign and Date)

6. SDD Suspension 5°C R&D Stability

Form No: TF2402-01

VERITA PHARMA

R&D Stability Report

Product Name: **SDD Suspension**	Product Code: **N/A**	Company Name: **The George Institute**
Dosage Form: **Suspension**	Batch No: **FP001071117**	Stability Study: **SP-0003-02**
Packaging: **Amber Plastic bottle with Child Resistant Cap**	Storage Temperature: **5°C ± 3°C**	Humidity: **N/A**

Time points		Initial	1 w	2 w	3 w	1 M	1.5 M	2 M	
Due date		15/11/17	22/11/17	29/11/17	06/12/17	15/12/17	01/01/18	15/01/18	
Test	Specifications								
Appearance	After shaking, a yellow liquid	complies	complies	complies	complies	complies	complies	complies	
pH *(after reconstitution)*	4.0 – 5.0	4.50	4.47	4.54	4.47	4.61	4.45	4.55	
Nystatin Assay *(after reconstitution)*	200,000 IU/ml *Assay precision fiducial limits: 95-105%* *UFL: NLT 95% of stated IU* *LFL: NMT 120 % of stated IU*	206,182 IU/ml	205,268 IU/ml	201,465 IU/ml	204,480 IU/ml	204.835 IU/ml	*Not available (*)*	207,986 IU/ml	
Colistin Sulphate Assay *(after reconstitution)*	10 mg/ml *Assay precision fiducial limits: 95-105%* *UFL: NLT 97% of stated mg* *LFL: NMT 110 % of stated mg*	10.2 mg/ml	9.8 mg/ml	9.9 mg/ml	9.8 mg/ml	9.8 mg/ml	*Not available (*)*	10.1 mg/ml	
Tobramycin Assay *(after reconstitution)*	8 mg/ml *7.2 mg/g –9.6 mg/g*	8.68 mg/ml	8.77 mg/ml	8.77 mg/ml	8.57 mg/ml	8.79 mg/ml	8.72 mg/ml	8.44 mg/ml	
Microbiological quality *(after reconstitution)*	Complies TAMC: 10^2 CFU/g TYMC: 10^1 CFU/g *E.coli*: Absence/g	complies						complies	
PET *(after reconstitution)*	Complies USP requirements							complies	
Date completed		05/12/17	14/12/17	20/12/17	22/12/17	16/01/18	16/01/18	07/03/18	

Comments: ***The tests with (*) have not been performed.***

Checked By:________________________ (Sign and Date) **Authorised By QA Manager:**__________________________ (Sign and Date)

ew only

For peer review only - http://bmjopen.bmj.com/site/about/guidelines.xhtml

7. SDD Suspension NF 25°C- 60%RH

VERITA PHARMA	**Stability Report**	**Page 1 of 1**

**Product Name: SDD Suspension NF**	**Product Code: N/A**	**Company Name: The George Institute**
**Dosage Form: suspension**	**Batch No: 1810005**	**Stability Study: SP-0006-01**
**Packaging: 240 ml amber plastic bottle with adapter and child resistant cap**	**Storage Temperature: 25°C ± 2°C**	**Humidity: 65 % RH ± 5 %RH**

**Time points**		**Initial**	**1 W**	**2 W**	**4 W**
**Due date**			29/01/19	01/02/19	18/02/19
**Test**	**Specifications**				
**Appearance**	After shaking, a yellow liquid	complies	complies	complies	complies
**pH**	4.0 – 5.0	4.58	4.50	4.59	4.72
**Nystatin Assay**	200,000 IU/ml<br>*Assay precision fiducial limits: 95-105%*<br>*UFL: NLT 95% of stated IU*<br>*LFL: NMT 120 % of stated IU*	204,141 IU/ml	199,838 IU/ml	200,830 IU/ml	202,077 IU/ml
**Colistin Sulphate Assay**	10 mg/ml<br>*Assay precision fiducial limits: 95-105%*<br>*UFL: NLT 97% of stated mg*<br>*LFL: NMT 110 % of stated mg*	10.4 mg/ml	10.0 mg/ml	9.7 mg/ml	9.7 mg/ml
**Tobramycin Assay**	8 mg/ml<br>*7.2 mg/g –9.6 mg/g*	7.81 mg/ml	8.53 mg/ml	7.97 mg/ml	8.00 mg/ml
**Microbiological quality**	Complies<br>TAMC: 10^2 CFU/g<br>TYMC: 10^1 CFU/g<br>*E.coli*: Absence/g	complies			complies
**PET**	Complies				complies
**Date completed**		08/11/2019	14/02/2019	20/02/2019	03/04/2019

Comments: ____

Checked By:______________________ (Sign and Date) **Authorised By QA Manager:**______________________ (Sign and Date)

© Copyright - Verita Pharma
Information contained herein this document is the property of Verita Pharma - unauthorised disclosure to the public is prohibited.

8. SyrSpend SF pH4 Dry – 30% reduction in SDD Powder for Suspension Formulation

VERITA PHARMA

Syrspend SF pH4 Dry — 30% reduction in SDD Powder for Suspension Formulation

The current SDD Powder for Suspension contains 13.65g of Syrspend SF pH4 Dry (Syrspend SF), per bottle. Some sites currently part of the SuDDICU Clinical Trial, are finding some difficulty when reconstituting and using the suspension due to the thickness of the reconstituted product. The proposal is to reduce the quantity of Syrspend SF by 30% to 9.56g per bottle.

Syrspend SF is a starch based, preservative free suspending vehicle. The Syrspend SF contains; Modified Food

Starch (viscosity agent), Sucralose (sweetener) and a citrate/citric acid (buffer). The material allows the Active Pharmaceutical Ingredients (API's) to stay suspended and easy to (re)homogenise and is practically inert for chemical actions.

There are 3 API's in the SDD Suspension; Colistin Sulphate and Tobramycin Sulphate which are soluble in water and therefore dissolved in the suspension, and Nystatin which is suspended in the mixture and rehomogenised after shaking. The inert nature of the Syrspend SF means there should be no chemical interaction and therefore the efficacy of the drug remains the same. The microbiological stability of the product is maintained by the addition of Potassium Sorbate and Citric Acid to the powder blend. Both of these materials are dissolved in the water and the concentration of these materials will not change with the 30% reduction of Syrspend SF.

The reduction of the Syrspend SF quantity according to the supplier's procedure for use, falls within the recommended range of Water / Syrspend SF ratio.

On review with a quality consultant, Cathrine Dahlgren from CNQuality, it was concluded that the Syrspend SF reduction would not influence the activity of the 3 API's or the chemical and microbiological stability.

Based on the information reviewed it was determined that the current stability studies completed on the SDD Powder for Suspension and SDD Suspension (reconstituted) would be valid for the new formulation with a 30% reduction Syrspend SF. We are required to and will complete concurrent stability studies on the new formulation. The protocols have been written and are being approved.

Regards,

24.09.2018

Martina Bachmaier

General Manager

VERITA PHARMA PTY LTD ABN 89 604 520 532

UNIT 3/4 ENDEAVOUR ROAD CARINGBAH NSW 2229

PO BOX 2502 TAREN POINT NSW 2229

*612 8536 4130 | INFO@VERITAPHARMA.COM.AU I WWW.VERITAPHARMA.COM.AU

9. PICnIC Labels (Box, SDD oral paste, SDD suspension)

KEEP OUT OF REACH OF CHILDREN

FOR CLINICAL TRIAL USE ONLY

CHIEF INVESTIGATOR: Dr Nazima Pathan

SPONSOR: University of Cambridge & Cambridge University Hospitals NHS Trust
Addenbrookes Hospital, Hills Road, Cambridge
CB2 0QQ
Tel:01223 245151

PROTOCOL: PICnIC (REC 20/WM/0061)

BOX CONTAINS:

1 x Bottle of SDD Gastric Powder for Suspension (Total volume once reconstituted 210ml)

When reconstituted each 10ml contains: 2,000,000 units Nystatin, 100mg Colistin Sulfate, 80mg Tobramycin (as Sulfate)

20 x syringes of 500mg SDD Oral Paste

Each syringe contains: 10mg Colistin Sulfate, 10mg Tobramycin (as Sulfate) and 125,000 units Nystatin

Directions for use and reconstitution: Per the PICnIC protocol

Storage Conditions: Store at 2-8°C Do Not Freeze
In use: May be stored at up to 25°C for up to 5 days from date dispensed below.

FOR HOSPITAL USE:

Patient Trial Number:
Patient Full Name:
Date dispensed: ___ / ___ / ___
Use by: ____ / ____ / ____

Batch #:
Expiry:

Kit #

ITEM M-XXX-01

Sponsor: University of Cambridge & Cambridge University Hospitals NHS Trust
Protocol: PICnIC (REC 20/WM/0061)

Keep out of reach of children

SDD ORAL PASTE 500mg

SINGLE USE ONLY

FOR CLINICAL TRIAL USE ONLY

Each syringe contains: 10mg Colistin Sulfate, 10mg Tobramycin (as Sulfate) and 125,000 units Nystatin

Batch: **Store at 2-8°C**
Expiry: **In use storage: <25°C up to 5 days**
Patient Trial No.:_______________

ITEM M-XXX-01

Keep out of reach of children

SDD GASTRIC POWDER FOR SUSPENSION (200ml)

SHAKE WELL BEFORE USE
FOR CLINICAL TRIAL USE ONLY
Protocol: PICnIC (REC 20/WM/0061)

BATCH:
EXPIRY:

Patient Trial No.: ___________
ONCE RECONSTITUTED DISCARD AFTER:
___ / ___ / ____

Sponsor:
University of Cambridge & Cambridge University Hospitals NHS Trust

WHEN RECONSTITUTED EACH 10ml CONTAINS:
2,000,000 units Nystatin, 100mg Colistin Sulfate, 80mg Tobramycin (as Sulfate)

Store at 2-8°C
For oral use as directed by PICnIC protocol
Once reconstituted, may be stored at room temperature (up to 25°C) for up to 5 days.

ITEM M-XXX-01

We would like to ask your permission to take some samples from your child during their PICU stay to help improve our understanding of infection control

We are part of a study looking at how we can improve infection control procedures within the PICU. To help us understand if these measures work, we need to know what 'normal' looks like in the unit. To do this, we are looking at samples taken from children before, during and after the study.

This is the period (before/during/after) and we will be taking samples from children admitted between (date) and (date)

We would like to take samples from your child at regular timepoints throughout the admission. The samples won't hurt your child and we will make them as comfortable as possible. When we can, we will take them whilst carrying out routine care of your child.

We will take: nasopharyngeal (swab from nose and throat), stool samples or rectal swabs, and where clinically indicated urine and sputum samples and a swab of any wounds your child may have.

There is no direct benefit to your child, this work will help to improve treatment for children in the future. You do not have to agree to these samples and it will not affect your child's medical care. We will destroy any samples we have that are not needed by the doctors/nurses.

All samples are stored and analysed anonymously, it will not be possible to link them to your child. All research like this is reviewed by an independent group, called a Research Ethics Committee, who protect you and your child's interests. PICnIC was reviewed by West Midlands Black Country Health Research Authority who agree it is being conducted in a correct and appropriate matter.

If you would like more information, please speak to your child's bedside nurse, a member of the research team (name number) or the doctor in charge of the study (name number)

We are very grateful for your help - thank you

NHS National Institute for Health Research

For peer review only - http://bmjopen.bmj.com/site/about/guidelines.xhtml

To be printed on local hospital headed paper

Paediatric Intensive Care and Infection Control (PICnIC) Study

Consent Form - Parent or Legal Guardian

Version 3.1, 25 September2020

To be completed by the Researcher:

Hospital name:	
PICnIC Study Number:	
Child's full name:	

To be completed by the Parent or Legal Guardian:

Once you have read and understood each statement – **if you agree, please write your initials in each box**

1.	I confirm that I have read and understand the Participant Information Sheet (version (please insert) dated(please insert)) for the above study. I have had the opportunity to consider the information, ask questions and have had these answered satisfactorily.	☐
2.	I understand that participation is voluntary, and that I am free to withdraw consent at any time, without giving any reason and without my child's medical care or legal rights being affected.	☐
3.	I agree for additional samples from my child to be taken, analysed, and stored as part of this research study.	☐
4.	I understand that relevant sections of my child's medical records and identifiable data collected during the study, held by the NHS, may be looked at by individuals from the Intensive Care National Audit & Research Centre (ICNARC) or regulatory authorities where it is relevant to my participation in this research.	☐
5.	I agree to complete a questionnaire about my views on conducting a research study.	☐
6.	I agree to be contacted for a telephone interview within the next 4 weeks. I understand that my contact details will be securely passed to the PICnIC study team at ICNARC and the University of Liverpool.	☐

Your signature:		Date:	
Your full name (PRINT):			
Researcher signature:		Date:	
Researcher full name (PRINT):			

Please sign and turn over to complete

For peer review only - http://bmjopen.bmj.com/site/about/guidelines.xhtml

Paediatric Intensive Care and Infection Control (PICnIC) Study

Parent or Legal Guardian contact information

To be completed by the Parent or Legal Guardian:

If you agree to be contacted for a telephone interview, please provide your details below:

**Telephone number:**	
**Mobile number:**	
**Email address:**	

1 copy for patient and parent/guardian; 1 copy for Investigator Site File; 1 copy to be kept with hospital notes

For peer review only

For peer review only - http://bmjopen.bmj.com/site/about/guidelines.xhtml

To be printed on local hospital headed paper

Paediatric Intensive Care and Infection Control (PICnIC) Study

Consent Form – Parent or Legal Guardian
Version 1.1, 10 September 2021

To be completed by the Researcher:

Hospital name:	
PICnIC Study Number:	
Child's full name:	

To be completed by the Parent or Legal Guardian:

Once you have read and understood each statement – **if you agree, please write your initials in each box**

1.	I confirm that I have read and understand the Participant Information Sheet (version (please insert) dated (please insert)) for the above study. I have had the opportunity to consider the information, ask questions and have had these answered satisfactorily.	☐
2.	I understand that participation is voluntary, and that I am free to withdraw consent at any time, without giving any reason and without my legal rights being affected.	☐
3.	I understand that relevant sections of my child's medical records and identifiable data collected during the study, held by the NHS, may be looked at by individuals from the Intensive Care National Audit & Research Centre (ICNARC) or regulatory authorities where it is relevant to my participation in this research.	☐
4.	I agree to be contacted for a telephone interview within the next two months. I understand that my contact details will be securely passed to the PICnIC study team at ICNARC and the University of Liverpool.	☐

Your signature:		Date:	
Your full name (PRINT):			
Researcher signature:		Date:	
Researcher full name (PRINT):			

Please sign and turn over to complete

For peer review only

For peer review only - http://bmjopen.bmj.com/site/about/guidelines.xhtml

Paediatric Intensive Care and Infection Control (PICnIC) Study

Parent or Legal Guardian contact information

To be completed by the Parent or Legal Guardian:

If you agree to be contacted for a telephone interview, please provide your details below:

**Telephone number:**	
**Mobile number:**	
**Email address:**	

1 copy for patient and parent/guardian; 1 copy for Investigator Site File; 1 copy to be kept with hospital notes

For peer review only

For peer review only - http://bmjopen.bmj.com/site/about/guidelines.xhtml

STANDARD PROTOCOL ITEMS: RECOMMENDATIONS FOR INTERVENTIONAL TRIALS

SPIRIT 2013 Checklist: Recommended items to address in a clinical trial protocol and related documents*

Section/item	Item No	Description	Pg No.
**Administrative information**			
Title	1	Descriptive title identifying the study design, population, interventions, and, if applicable, trial acronym	1
Trial registration	2a	Trial identifier and registry name. If not yet registered, name of intended registry	3
	2b	All items from the World Health Organization Trial Registration Data Set	throughout
Protocol version	3	Date and version identifier	16
Funding	4	Sources and types of financial, material, and other support	20
Roles and responsibilities	5a	Names, affiliations, and roles of protocol contributors	1 & 19
	5b	Name and contact information for the trial sponsor	16 &20
	5c	Role of study sponsor and funders, if any, in study design; collection, management, analysis, and interpretation of data; writing of the report; and the decision to submit the report for publication, including whether they will have ultimate authority over any of these activities	20
	5d	Composition, roles, and responsibilities of the coordinating centre, steering committee, endpoint adjudication committee, data management team, and other individuals or groups overseeing the trial, if applicable (see Item 21a for data monitoring committee)	15
**Introduction**			
Background and rationale	6a	Description of research question and justification for undertaking the trial, including summary of relevant studies (published and unpublished) examining benefits and harms for each intervention	5
	6b	Explanation for choice of comparators	5
Objectives	7	Specific objectives or hypotheses	6

For peer review only - http://bmjopen.bmj.com/site/about/guidelines.xhtml

Trial design	8	Description of trial design including type of trial (eg, parallel group, crossover, factorial, single group), allocation ratio, and framework (eg, superiority, equivalence, noninferiority, exploratory)	6

Methods: Participants, interventions, and outcomes

Study setting	9	Description of study settings (eg, community clinic, academic hospital) and list of countries where data will be collected. Reference to where list of study sites can be obtained	6
Eligibility criteria	10	Inclusion and exclusion criteria for participants. If applicable, eligibility criteria for study centres and individuals who will perform the interventions (eg, surgeons, psychotherapists)	6&7
Interventions	11a	Interventions for each group with sufficient detail to allow replication, including how and when they will be administered	7
	11b	Criteria for discontinuing or modifying allocated interventions for a given trial participant (eg, drug dose change in response to harms, participant request, or improving/worsening disease)	8
	11c	Strategies to improve adherence to intervention protocols, and any procedures for monitoring adherence (eg, drug tablet return, laboratory tests)	10
	11d	Relevant concomitant care and interventions that are permitted or prohibited during the trial	7&8
Outcomes	12	Primary, secondary, and other outcomes, including the specific measurement variable (eg, systolic blood pressure), analysis metric (eg, change from baseline, final value, time to event), method of aggregation (eg, median, proportion), and time point for each outcome. Explanation of the clinical relevance of chosen efficacy and harm outcomes is strongly recommended	11
Participant timeline	13	Time schedule of enrolment, interventions (including any run-ins and washouts), assessments, and visits for participants. A schematic diagram is highly recommended (see Figure)	6
Sample size	14	Estimated number of participants needed to achieve study objectives and how it was determined, including clinical and statistical assumptions supporting any sample size calculations	3
Recruitment	15	Strategies for achieving adequate participant enrolment to reach target sample size	6

Methods: Assignment of interventions (for controlled trials)

Allocation:

For peer review only - http://bmjopen.bmj.com/site/about/guidelines.xhtml

Sequence generation	16a	Method of generating the allocation sequence (eg, computer-generated random numbers), and list of any factors for stratification. To reduce predictability of a random sequence, details of any planned restriction (eg, blocking) should be provided in a separate document that is unavailable to those who enrol participants or assign interventions	1&6
Allocation concealment mechanism	16b	Mechanism of implementing the allocation sequence (eg, central telephone; sequentially numbered, opaque, sealed envelopes), describing any steps to conceal the sequence until interventions are assigned	NA
Implementation	16c	Who will generate the allocation sequence, who will enrol participants, and who will assign participants to interventions	6
Blinding (masking)	17a	Who will be blinded after assignment to interventions (eg, trial participants, care providers, outcome assessors, data analysts), and how	NA
	17b	If blinded, circumstances under which unblinding is permissible, and procedure for revealing a participant's allocated intervention during the trial	NA
**Methods: Data collection, management, and analysis**			
Data collection methods	18a	Plans for assessment and collection of outcome, baseline, and other trial data, including any related processes to promote data quality (eg, duplicate measurements, training of assessors) and a description of study instruments (eg, questionnaires, laboratory tests) along with their reliability and validity, if known. Reference to where data collection forms can be found, if not in the protocol	9,11, 12, 14
	18b	Plans to promote participant retention and complete follow-up, including list of any outcome data to be collected for participants who discontinue or deviate from intervention protocols	10
Data management	19	Plans for data entry, coding, security, and storage, including any related processes to promote data quality (eg, double data entry; range checks for data values). Reference to where details of data management procedures can be found, if not in the protocol	10
Statistical methods	20a	Statistical methods for analysing primary and secondary outcomes. Reference to where other details of the statistical analysis plan can be found, if not in the protocol	12
	20b	Methods for any additional analyses (eg, subgroup and adjusted analyses)	14

For peer review only - http://bmjopen.bmj.com/site/about/guidelines.xhtml

	20c	Definition of analysis population relating to protocol non-adherence (eg, as randomised analysis), and any statistical methods to handle missing data (eg, multiple imputation)	13
**Methods: Monitoring**			
Data monitoring	21a	Composition of data monitoring committee (DMC); summary of its role and reporting structure; statement of whether it is independent from the sponsor and competing interests; and reference to where further details about its charter can be found, if not in the protocol. Alternatively, an explanation of why a DMC is not needed	16
	21b	Description of any interim analyses and stopping guidelines, including who will have access to these interim results and make the final decision to terminate the trial	NA
Harms	22	Plans for collecting, assessing, reporting, and managing solicited and spontaneously reported adverse events and other unintended effects of trial interventions or trial conduct	10&11
Auditing	23	Frequency and procedures for auditing trial conduct, if any, and whether the process will be independent from investigators and the sponsor	16
**Ethics and dissemination**			
Research ethics approval	24	Plans for seeking research ethics committee/institutional review board (REC/IRB) approval	15
Protocol amendments	25	Plans for communicating important protocol modifications (eg, changes to eligibility criteria, outcomes, analyses) to relevant parties (eg, investigators, REC/IRBs, trial participants, trial registries, journals, regulators)	Not provided
Consent or assent	26a	Who will obtain informed consent or assent from potential trial participants or authorised surrogates, and how (see Item 32)	9
	26b	Additional consent provisions for collection and use of participant data and biological specimens in ancillary studies, if applicable	9
Confidentiality	27	How personal information about potential and enrolled participants will be collected, shared, and maintained in order to protect confidentiality before, during, and after the trial	9 &10
Declaration of interests	28	Financial and other competing interests for principal investigators for the overall trial and each study site	20
Access to data	29	Statement of who will have access to the final trial dataset, and disclosure of contractual agreements that limit such access for investigators	16

review only

Ancillary and post-trial care	30	Provisions, if any, for ancillary and post-trial care, and for compensation to those who suffer harm from trial participation	NA
Dissemination policy	31a	Plans for investigators and sponsor to communicate trial results to participants, healthcare professionals, the public, and other relevant groups (eg, via publication, reporting in results databases, or other data sharing arrangements), including any publication restrictions	15
	31b	Authorship eligibility guidelines and any intended use of professional writers	Not provided
	31c	Plans, if any, for granting public access to the full protocol, participant-level dataset, and statistical code	Full protocol is
**Appendices**			
Informed consent materials	32	Model consent form and other related documentation given to participants and authorised surrogates	Supplementary
Biological specimens	33	Plans for collection, laboratory evaluation, and storage of biological specimens for genetic or molecular analysis in the current trial and for future use in ancillary studies, if applicable	7

*It is strongly recommended that this checklist be read in conjunction with the SPIRIT 2013 Explanation & Elaboration for important clarification on the items. Amendments to the protocol should be tracked and dated. The SPIRIT checklist is copyrighted by the SPIRIT Group under the Creative Commons "Attribution-NonCommercial-NoDerivs 3.0 Unported" license.

For peer review only

BMJ Open

Use of selective gut decontamination in critically ill children: protocol for the Paediatric Intensive Care and Infection Control (PICnIC) pilot study

Journal:	*BMJ Open*
Manuscript ID	bmjopen-2022-061838.R1
Article Type:	Protocol
Date Submitted by the Author:	15-Feb-2022
Complete List of Authors:	Brown, Alanna; Intensive Care National Audit and Research Centre<br>Ferrando, Paloma; Intensive Care National Audit and Research Centre<br>Popa, Mariana; University of Liverpool, Institute of Life and Human Sciences<br>de la Fuente, Gema Milla; Intensive Care National Audit and Research Centre<br>Pappachan, John; Southampton Children's Hospital, PICU<br>Cuthbertson, Brian; Sunnybrook Health Sciences Centre, Department of Critical Care<br>Drikite, Laura; Intensive Care National Audit and Research Centre, Clinical Trials Unit<br>Feltbower, Richard; University of Leeds<br>Gouliouris, Theodore; Cambridge University, Department of Medicine<br>Sale, Isobel; PPI representative<br>Shulman, Robert; University College London Hospitals NHS Foundation Trust, Department of Pharmacy<br>Tume, Lyvonne; University of Salford, School of Health and Society<br>Myburgh, John; The George Institute for Global Health<br>Woolfall, Kerry; University of Liverpool<br>Harrison, David; ICNARC<br>Mouncey, Paul; ICNARC, Clinical Trials Unit<br>Rowan, Kathryn; ICNARC<br>Pathan, Nazima; University of Cambridge, Department of Paediatrics; Cambridge University Hospitals NHS Foundation Trust,
**Primary Subject Heading**:	Paediatrics
Secondary Subject Heading:	Intensive care
Keywords:	Infection control < INFECTIOUS DISEASES, Paediatric intensive & critical care < ANAESTHETICS, MICROBIOLOGY

SCHOLARONE™ Manuscripts

Use of selective gut decontamination in critically ill children: protocol for the Paediatric Intensive Care and Infection Control (PICnIC) pilot study

AUTHORS

Alanna Brown[1], Paloma Ferrando-Vivas[1], Mariana Popa[2], Gema Milla de la Fuente[1], John Pappachan[3], Brian Cuthbertson[4], Laura Drikite[1], Richard Feltbower[5], Theodore Gouliouris[6], Isobel Sale[7], Robert Shulman[8], Lyvonne N Tume[9], John Myburgh[10], Kerry Woolfall[2], David A Harrison[1], Paul R Mouncey[1], Kathryn Rowan[1], Nazima Pathan[11].

Affiliations

[1] Clinical Trials Unit, Intensive Care National Audit and Research Centre, London, UK
[2] Department of Public Health, University of Liverpool, Liverpool, UK
[3] Paediatric Intensive Care Unit, Southampton Children's Hospital, University of Southampton, Southampton, UK
[4] Department of Critical Care Medicine, Sunnybrook Health Sciences Center, Toronto, Canada.
[5] Division of Epidemiology, University of Leeds, Leeds, UK
[6] Clinical Microbiology and Public Health Laboratory, Addenbrooke's Hospital, Cambridge University Hospital NHS Foundation Trust, Cambridge, UK
[7] PPI representative, UK
[8] Department of Pharmacy, University College London Hospitals NHS Foundation Trust, London, UK
[9] Intensive Care Unit, Alder Hey Children's NHS Foundation Trust Liverpool, UK
[10] George Institute for Global Health, Faculty of Medicine, University of New South Wales, Sydney, NSW, Australia
[11] Department of Paediatrics, University of Cambridge, Addenbrooke's Hospital, Cambridge, UK

Corresponding author

Dr Nazima Pathan

University Lecturer and Consultant in Paediatric Intensive Care
Department of Paediatrics,
University of Cambridge,

For peer review only - http://bmjopen.bmj.com/site/about/guidelines.xhtml

Level 8, Addenbrookes Hospital, Hills Road
Cambridge CB2 0QQ
Tel: 020 7269 9277
Email: np409@cam.ac.uk

Keywords

Selective digestive tract decontamination, Invasive mechanical ventilation, randomised clinical trial, paediatric critical care

Word count: Abstract (320), Main manuscript (3814/4000)

For peer review only

For peer review only - http://bmjopen.bmj.com/site/about/guidelines.xhtml

ABSTRACT

Introduction

Healthcare-associated infections (HCAI) are a major cause of morbidity and mortality in critically ill children. In critically ill adults, there are data that suggest the use of Selective Decontamination of the Digestive tract (SDD), alongside standard infection control measures reduce mortality and the incidence of HCAIs. SDD-enhanced infection control has not been compared directly with standard infection prevention strategies in the Paediatric Intensive Care Unit (PICU) population. The aim of this pilot study is to determine the feasibility of conducting a multicentre cluster randomised controlled trial (cRCT) in critically ill children comparing SDD with standard infection control.

Methods and analysis

PICnIC is a parallel group pilot cRCT, with integrated mixed-methods study, comparing incorporation of SDD into infection control procedures to standard care. After a one-week pre-trial ecology surveillance period, recruitment to the cRCT will run for a period of 18 weeks, comprising: (1) baseline control period (2) pre, mid and post-trial ecology surveillance periods and (3) intervention period. Six PICUs (in England, UK) will begin with usual care in Period One, then will be randomised 1:1 by the trial statistician using computer-based randomisation, to either continue to deliver usual care or commence delivery of the intervention (SDD) in Period Two. Outcomes measures include parent and healthcare professionals' views on trial feasibility, adherence to the SDD intervention, estimation of recruitment rate and understanding of potential patient-centred primary and secondary outcome measures for the definitive trial. The planned recruitment for the cRCT is 324 participants.

Ethics and dissemination

The trial received favourable ethical opinion from West Midlands – Black Country Research Ethics Committee (reference: 20/WM/0061) and approval from the Health Research Authority (IRAS number: 239324). Informed consent is not required for SDD intervention or anonymised data collection but is sought for investigations as part of the study, any identifiable data collected and monitoring of medical records. Results will be disseminated via publications in peer-reviewed medical journals.

Trial registration

ISRCTN40310490.

For peer review only - http://bmjopen.bmj.com/site/about/guidelines.xhtml
For peer review only

Strengths and limitation of this study

- The study will examine the processes that are important in a future clinical trial of SDD in the PICU setting.
- The study uses a GMP-certified commercial SDD preparation under licence from the George Institute, Australia (Verita Pharma Pty, Australia).
- The study will evaluate the perspective of parents and the views of stakeholders including caregivers and the multi-disciplinary team on the processes needed to undertake a trial of SDD in the PICU.
- The use of SDD-enhanced infection control requires it to be implemented unit-wide, along with the support of local microbiology, pharmacy and infection control teams for delivery and monitoring in sites randomised to implement it.
- This is a pilot study, so is not designed to assess effectiveness of the intervention; the study outcomes will inform the feasibility of a future trial based on clinical outcomes.

INTRODUCTION

In critically ill children, healthcare-associated infections (HCAIs) are a major cause of morbidity and mortality, with a reported prevalence of 7-14%.[1-5] HCAIs can develop either as a direct result of healthcare interventions such as medical or surgical treatment, or from being in contact with a healthcare setting. In the critical care setting, the high use of invasive devices such as endotracheal tubes, vascular and urinary catheters increase the risk of secondary infection by opportunistic organisms. In particular, respiratory HCAIs (ventilator associated pneumonia, VAP) may occur with spread of commensal and other organisms from oro-pharyngeal and upper gastrointestinal compartments into the lungs.[4,5]

Evidence from adult intensive care studies suggests that using Selective Decontamination of the Digestive tract (SDD) alongside standard infection control measures reduces mortality and ventilator-associated pneumonia (VAP).[6,7] It has been shown that the use of SDD influences the microbiological ecology of the unit, thereby reducing incidence of HCAIs in both exposed and non-exposed patients. Despite this, SDD has not been routinely adopted due to concerns that it may promote antimicrobial resistance.[6,8] Recent ecological studies conducted in adult intensive care have found that SDD was associated with a reduction in antibiotic utilisation;[9-13] two large cluster randomised controlled trials (cRCTs) have been recently undertaken to further evaluate the clinical effects of SDD in adult intensive care.

For peer review only - http://bmjopen.bmj.com/site/about/guidelines.xhtml

One reported no change in the incidence of blood stream infections, but the incidence of ventilator acquired pneumonia and overall antimicrobial use have not yet been reported.[14] Another large-scale multicentre study using the same formulation as the PICNIC study has recently completed enrolment of critically ill adults in Australia, Canada and the UK.[15]

SDD has yet to be compared directly with modern infection control protocols within the paediatric intensive care unit (PICU) population. The only trial data suggest a reduction in incidence of VAP but not mortality, however the study was underpowered and the observed mortality was very low.[16] Therefore, a clinical trial comparing SDD with standard infection control methods is required. Given the paucity of data describing the use of SDD in PICU and to establish the appropriate safety and ecological monitoring protocols, it is first imperative to establish whether a large, multicentre trial is feasible.

The PICnIC pilot study is a feasibility study designed to determine whether it is possible to conduct a cRCT of SDD in critically ill children who are likely to be ventilated for >48 hours, and to explore and test the acceptability of key components of the study to healthcare professionals and families of patients.

METHODS AND ANALYSIS

Aim

To determine whether it is feasible to conduct a multicentre trial in critically ill children comparing SDD with standard infection control procedures.

Objectives

- To test the ability to randomise PICUs to either control or intervention
- To test the willingness and ability of healthcare professionals to screen and recruit eligible children
- To estimate the recruitment rate of eligible children
- To test adherence to the SDD protocol (including tolerance and application of a standardised SDD paste and suspension in a paediatric population)
- To test the procedures for assessing and collecting selected clinical and ecological outcomes and for adverse event (AE) reporting
- To assess the generalisability of the study
- To explore parent and healthcare professional views on the acceptability of the proposed trial, including recruitment and consent procedures and patient centred outcomes

For peer review only - http://bmjopen.bmj.com/site/about/guidelines.xhtml

Study Setting - Six PICUs based in England, UK with a diverse geographical/demographic population representative of national (UK) PICU activity and size.

Design

External pilot, parallel group cRCT with integrated mixed-methods study. After a one-week pre-trial ecology surveillance period, recruitment to the cRCT will run for a period of 18 weeks, comprising: (1) baseline control period ('Period One' - weeks 2-9); (2) mid-trial ecology surveillance period (week 10); and (3) intervention period ('Period Two' - weeks 11-19). On completion of Period Two, an additional one-week post-trial ecology surveillance period will be carried out (Figure 1). Sites will be randomised 1:1 by the trial statistician using computer-based randomisation, to either continue to deliver usual care or commence delivery of the intervention (SDD) in Period Two (Figure 2).

For peer review only

Screening

Potentially eligible patients presenting to the participating unit will be screened against the inclusion/exclusion criteria by the local clinical team, supported by the site research team. Screening Logs will record the reason patients are eligible but are subsequently not enrolled.

Eligibility – Ecology Surveillance Periods

Inclusion criteria

- All patients admitted to the PICU, regardless of ventilation status, during any of the three ecological surveillance periods

Exclusion criteria

- None

Eligibility – Period One and Period Two

Inclusion Criteria

- >37 weeks corrected gestational age to <16 years
- Receiving invasive mechanical ventilation, expected to last at least 48 hours
- Expected to remain on invasive mechanical ventilation until the day after tomorrow (from time of screening)

Exclusion Criteria

For peer review only - http://bmjopen.bmj.com/site/about/guidelines.xhtml

- Known allergy, sensitivity or interaction to polymyxin E (colistin), tobramycin or nystatin
- Known to be pregnant
- Death perceived as imminent

Microbiology Sampling

Samples taken:

- Nasopharyngeal
- Stool/rectal swabs
- Urine (if clinically indicated)
- Sputum/secretions from the endotracheal tube (If clinically indicated)
- Wound swabs, if present (if clinically indicated)

During the designated ecology surveillance periods, samples will be taken on admission and then taken on a Friday, if the patient has not had samples taken in the previous 48 hours.

During Periods One and Two, samples will be taken on admission and then twice weekly until discharge. For patient stays of less than 7 days, samples should be taken on the day of discharge.

Trial Intervention

During the Ecology Surveillance periods, Period One and for sites randomised to usual care in Period Two, there is no intervention. Patients will receive all standard infection control measures (per the site's specific policies) but will receive no study specific intervention.

For sites randomised to the intervention in Period Two, it will form part of the standard infection control strategy in the participating PICU. In addition to usual care, an SDD-enhanced infection control regimen will be delivered to all eligible patients using a GMP-certified commercial SDD preparation under licence from The George Institute, Australia (Verita Pharma Pty, Australia). (Within Supplementary File 1, see Supplementary Table 1 for SDD administration details, Supplementary Table 2-3 for composition and characteristics of SDD, Supplementary Table 4-5 for SDD formulation, Supplementary Appendices 1 for GMP licence, Supplementary Appendices 2-8 for SDD stability data and Supplementary Appendices 9 for PICnIC labels). Dosing of the SDD suspension will be calculated according to age (Table 1):

Table 1: SDD suspension dosing

For peer review only - http://bmjopen.bmj.com/site/about/guidelines.xhtml

	0 – 4 years	5 – 12 years	≥13 years
**Polymyxin E (Colistin)**	25mg	50mg	100mg
**Tobramycin**	20mg	40mg	80mg
**Nystatin**	0.5 x 106 IU	1 x 106 IU	2 x 106 IU
	**2.5ml**	**5ml**	**10ml**

1. A six-hourly topical, application of a pea-sized (0.5g) SDD paste containing 2% polymyxin E (colistin), 2% tobramycin and 2% nystatin to the buccal mucosa and oropharynx; and

2. A six-hourly administration of SDD suspension administered via the most proximal feeding tube into the stomach containing polymyxin E (colistin), tobramycin and nystatin.

SSD preparations will be distributed by the manufacturer in temperature-controlled (2-8ºC), patient specific kits to the participating hospital pharmacies. Each kit contains a 5-day supply of SDD treatment which includes a bottle of SDD suspension powder for reconstitution and 20 x1ml syringes containing Oral Paste. When a kit is allocated to a patient and the SDD powder is reconstituted into suspension, the kit can be stored at room temperature (≤27ºC) for up to 5 days. SDD treatment should be started within 6 hours of the patient being identified as eligible and continue for a maximum of 30 days (treatment period).

Treatment will continue until the patient is extubated or no longer mechanically ventilated (in tracheostomised patients). Patients subsequently re-intubated (either during this PICU admission or readmission to PICU from another inpatient area) during the treatment period will restart the intervention. All other usual care will be provided at the discretion of the treating clinical team.

Consent procedures

Children who are eligible for PICnIC will often become so during a period of life-threatening illness. This is a stressful time for parents/guardians during which time there are ethical concerns both about the burden placed of trying to understand the trial and their ability to provide informed consent.[17]

For peer review only - http://bmjopen.bmj.com/site/about/guidelines.xhtml

Posters displayed throughout the PICU will explain ecology sampling procedures and invite parents/guardians to have further discussions with research staff. The SDD intervention will be delivered, in additional to standard infection control policies, to all eligible patients in sites randomised to the SDD intervention during Period Two. Research staff will not seek individual consent for this. This approach is in line with guidance from the Ottawa Statement on the ethical design and conduct of cRCT.[18,19] (See supplementary file 2 for poster and consent forms).

Consent will be required for:

- Any additional study-specific samples to be taken, stored, and analysed prior to being taken solely for the study and not as part of routine care.
- Identifiable data collected and processed for participation in the mixed methods aspects of the study (questionnaire and interview).
- Monitoring of medical records.

Consent will not be required for:

- Samples taken as part of routine care (e.g. admission samples).
- Anonymised data collection and processing from routine sources.
- Delivery of SDD. For PICUs randomised to the intervention during Period Two, all children meeting the eligibility criteria will receive SDD as the standard practice.

Children may be withdrawn from trial specific data collection by the request of parents who decline participation in the research. All data collected up to the point of withdrawal will be retained and included in the study analysis.

If the patient has died, the parents/guardians will be approached for consent for the monitoring of medical records and identifiable data collected, and for the interview aspect of the study once established whether it is appropriate to approach for consent.

Data collection

Detailed guidance for the collection of data will be provided in the trial-specific standard operating procedure. It will include:

- Demographics
- Date/time of commencing mechanical ventilation
- Date/time identified as eligible
- Antibiotic usage throughout admission (route, type, duration, frequency, dose)
- Date/time of final extubation

For peer review only - http://bmjopen.bmj.com/site/about/guidelines.xhtml

- Details of any HCAI (confirmed/presumed)
- Date/time of PICU discharge
- Date/time of hospital discharge
- SDD delivery (dose for age, date time first dose, dose per day, change of dose, protocol deviation and reason for deviation)

No identifiable participant data will be required by the ICNARC Clinical Trials Unit (CTU) and all participant data will be stored securely. ICNARC is registered under the Data Protection Act (1998), and all ICNARC CTU staff have undergone data protection and International Conference on Harmonisation (ICH) Good Clinical Practice (GCP) training.

Patients will be followed-up until discharge from a participating PICU. Data entered onto the secure trial database will undergo validation checks for completeness, accuracy, and consistency of data. Queries on incomplete, inconsistent, or non-adherent data will be sent to the relevant PICU team for resolution.

Routine linkage will be made for all patients with the Paediatric Intensive Care Audit Network (PICANet) through the PICANet ID and trial number to obtain:

- Baseline demographics and risk factors, including predicted risk of death
- Secondary outcomes of critical care and acute hospital mortality, organ support received

Safety monitoring

Adverse event (AE) reporting will follow the Health Research Authority (HRA) guidelines on safety reporting in studies which do not use Investigational Medicinal Products.

AEs will be recorded during Period Two, from enrolment until PICU discharge (including readmissions from other inpatient areas until 30 days from being identified as eligible). For patients receiving SDD intervention, only AEs deemed possibly, probably or definitely related to the trial intervention should be reported to ICNARC CTU, apart from NG tube blockages which should be reported even if not deemed related. For patients receiving usual care, only NG tube blockages will be reported.

The following events have been prespecified as potential AEs:

- NG tube blockage
- Choking on paste
- Allergic reaction to SDD

For peer review only - http://bmjopen.bmj.com/site/about/guidelines.xhtml

The following events are exempt for reporting as AEs or SAEs

- Deterioration of condition or death that is not related to the trial intervention
- AEs of other drugs not specified in the protocol.

Any event classified as 'severe', 'life-threatening' or 'fatal' in severity is considered a serious AE (SAE) and must be reported to ICNARC CTU. If the SAE is evaluated by the Chief Investigator or clinical member of the Trial Management Group (TMG) as a related and unexpected SAE, the ICNARC CTU will submit a report to the Research Ethics Committee (REC) within 15 calendar days.

Patient and Public Involvement

Caregivers of children admitted to PICU and a former patient were involved in prioritising the outcomes and designing the study protocol. Their input has continued with PPI representatives on the study oversight panel. A patient representative (former PICU patient) is a co-investigator and is an author of this manuscript.

Outcome measures

As this is a pilot study, outcome measures for the study will be focused on assessing the feasibility of a larger scale definitive study.

The ability to randomise PICUs to either control or intervention will be assessed by the successful random assignment of three PICUs to the intervention without delay to subsequent phases of the trial.

The willingness and ability of healthcare professionals to screen and recruit eligible children will be assessed by the proportion of eligible children recorded on study screening logs successfully recruited to the pilot cRCT and the reported reasons for non-recruitment.

The potential recruitment rate for a future definitive cRCT trial of SDD-enhanced infection control in eligible children will be estimated by combining the proportion of eligible children recruited to the pilot cRCT with the size of the potentially eligible population (estimated from nesting the screening log data from participating PICUs within the national UK PICU data from PICANet).

For peer review only - http://bmjopen.bmj.com/site/about/guidelines.xhtml

Adherence to the SDD protocol will be assessed by the proportion of eligible children allocated to the intervention receiving (a) both elements and (b) each individual element of the SDD intervention, the number of days on which these elements were received relative to days eligible for the SDD intervention and the reported reasons for nonadherence.

Procedures for assessing and collecting selected clinical and ecological outcomes and for AE reporting will be assessed by the proportion of children with complete data for these outcomes including, for ecological outcomes, the proportion consenting to additional study specific sample collection.

Generalisability of the study results to all UK PICUs will be assessed by comparing baseline characteristics and outcomes for children recruited to the pilot cRCT with data from all potentially eligible children (receiving invasive mechanical ventilation for at least 3 calendar days) within participating PICUs and within all UK PICUs (from PICANet).

With the aim of understanding potential patient-centred primary and secondary outcome measures for the definitive cRCT, the following potential outcome measures will be reported:

- HCAI (confirmed/presumed) and microbiology results (if positive sample);
- Hospital mortality;
- PICU mortality;
- Mortality within 30 days post enrolment;
- Length of hospital stay;
- Length of stay in PICU;
- Duration of mechanical ventilation; and
- Organ support received.

Statistical methods

Sample size

The PICnIC pilot study is set up to test the feasibility of the protocol to recruit eligible patients. Therefore, there is no primary outcome to be compared between the two groups and, hence, a usual power calculation to determine sample size is not appropriate. Instead, the sample size has been determined to be adequate to estimate critical parameters to be tested to a necessary degree of precision. Based on available data from PICANet, it is anticipated participating sites will see approximately 4.5 eligible children per week, therefore the anticipated recruitment rate is 3 children per PICU per week providing a total of

For peer review only - http://bmjopen.bmj.com/site/about/guidelines.xhtml

approximately 324 children in 18 weeks, of which 90 would receive the intervention. The sample size of children receiving usual care would be sufficient to estimate a binary outcome present in 20% of the population with a precision of ±5%.

Analysis

An overview of the planned analyses for the PICnIC pilot study is provided below. The full statistical analysis plan will be lodged on the trial website ahead of database lock.

A CONSORT flow diagram will be used to summarise the number and percentage of children screened, recruited and followed-up. This will include the proportion of eligible children successfully recruited and the reported reasons for non-recruitment.

Recruitment to the pilot cRCT will be presented as a rate per site per week over the two recruitment periods, overall, by treatment group and by site. Potential reasons for variation in recruitment rates will be explored. The potential recruitment rate for a future definitive cRCT will be estimated by combining the proportion of eligible children recruited to the pilot cRCT with the size of the potentially eligible population (estimated from PICANet).

Baseline demographic and clinical data will be summarised overall and for each of the two treatment groups in each of the two time periods but not subjected to statistical testing.
The proportion of eligible children allocated to the intervention that received both elements and each individual element of the SDD intervention will be reported as well as the number of days on which these elements were received relative to days eligible for the SDD intervention. The reported reasons for nonadherence will be detailed.

Data completeness of clinical and ecological outcomes and for AE reporting will be summarised.

Patient characteristics for children recruited to the pilot cRCT will be compared with those for potentially eligible children within participating PICUs and within all UK PICUs.
Potential patient-centred clinical outcome measures for the definitive trial will be estimated and reported (proportion or mean and standard deviation, and intracluster correlation). As a pilot cRCT, there will be no statistical testing for any of the summary measures.
Comparisons between groups will be used to estimate the potential magnitude of the treatment effect; p values will not be calculated or quoted.[20] To account for cluster randomisation, we will use multilevel logistic or generalised linear regressions in the above analyses.

For peer review only - http://bmjopen.bmj.com/site/about/guidelines.xhtml

Mixed methods study

The mixed methods study will involve a questionnaire and interviews with parents / legal guardians of children involved in the pilot cRCT as well as focus groups, interviews and an online survey with healthcare professionals. These will be used to review and explore:

- parent views on
  - the acceptability of a definitive trial that includes the SDD intervention.
  - the acceptability of the recruitment and consent procedures for the definitive trial, including all proposed information materials.
  - important, relevant, patient-centred primary and secondary outcomes for a definitive trial.
- healthcare professionals' views on
  - the acceptability of implementation of the SDD intervention, recruitment and consent procedures.
  - the acceptability of collecting data to assess the selected clinical and ecological data.
  - the acceptability of the SDD intervention and to confirm interest in participation in a definitive trial in the wider PICU community.

Inclusion criteria

- Parents/legal guardians of children involved in the pilot cRCT, including those who withdraw from data collection
- Healthcare professionals (including doctors, nurses, physios, pharmacists) working in PICUs that participate in the pilot cRCT.

Exclusion criteria

- Parents/legal guardians who do not speak English

Parents/legal representative recruitment and consent

Healthcare professionals will seek consent from parents of recruited children, including those who withdraw from data collection, to complete a questionnaire or register interest in a telephone or online interview. Questionnaires (n=~100) will be completed after pilot trial recruitment discussions and interviews will be conducted by the UoL team within a month until information power[21] is reached (n=~15-25 based on previous studies)

Healthcare professionals' recruitment and consent

For peer review only - http://bmjopen.bmj.com/site/about/guidelines.xhtml

Healthcare professionals involved in the pilot cRCT will be invited via email to participate in a virtual focus group (n=2) or interview (≥10 depending on information power). An online survey will be distributed through UK PICU networks.

Data Analysis of Mixed-Methods study

Interviews and focus groups will be transcribed, checked and anonymised as the study progresses. QSR NVivo software will be used to assist in the organisation and indexing of qualitative data. Whilst thematic analysis[22,23] will draw on the Theoretical Framework of acceptability.[24,25] The focus will be modified to fit with the criterion of catalytic validity, whereby findings should be relevant to future research and practice (in particular, the design of, and information to inform decisions on the progression to, a definitive cRCT). Quantitative data from parent questionnaires and the online survey will be analysed using SPSS software, descriptive statistics and exact tests will be used, as appropriate. Data from each method will be analysed separately then synthesised through the use of constant comparative analysis.[26]

ETHICS AND DISSEMINATION

The PICnIC pilot study will be conducted in accordance with the approved Trial Protocol, ICH GCP guidelines, the Data Protection Act (2018), the Mental Capacity Act (2005), as well as the ICNARC CTU's research policies and procedures.

The study received favourable ethical opinion from West Midlands – Black Country Research Ethics Committee (Ref: 20/WM/0061) and approval from the HRA (IRAS number: 239324).

Informed consent is not required for SDD intervention or anonymised data collection but is sought for investigations as part of the study, any identifiable data collected and monitoring of medical records.

The final report, including a detailed description of the trial, results and recommendations for future policy and practice and future research, will be submitted to the National Institute of Health Research Health Technology Assessment (NIHR HTA) Programme. Articles will be prepared for publication in peer-reviewed scientific journals, as well as relevant professional journals.

Oversight

For peer review only - http://bmjopen.bmj.com/site/about/guidelines.xhtml

The TMG, led by the chief investigator, is responsible for the management of PICnIC. It meets regularly and includes the Investigators and ICNARC CTU trial team. PICnIC is managed by the ICNARC CTU in accordance with the Medical Research Council's Good Research Practice: Principles and Guidelines[27] which is based on the ICH guidelines on GCP[28] principles and the UK Department of Health's Policy Framework for Health and Social Care Research.[29]

A majority independent TSC has been established to monitor trial progress and includes PPI representatives, experienced clinicians and researchers/statisticians, in addition to the chief investigator and head of research at ICNARC. An independent DMEC, comprising experienced clinicians and statisticians, has been established to monitor patient recruitment and retention, adherence and safety.

Cambridge University Hospitals NHS Foundation Trust and The University of Cambridge is the trial sponsor. As the sponsor is an NHS organisation, NHS indemnity will apply for legal liability arising from the design, management and conduct of the research.

Ownership of the data sits with the sponsor with collaboration agreements in place to allow access to necessary partners on the grant. Once a final anonymised dataset is created at the end of the study, requests for access to data will be reviewed and approved by the TMG.

Trial Status

The paper presents protocol v4.1, dated 17 December 2021. At the time of submission, patient recruitment was ongoing. Recruitment commenced in September 2021 with recruitment planned to complete in February 2022. Follow up data collection will continue until the end of March 2022.

Contributors

NP is chief investigator. AB is trial manager. PFV, MP, BC, RF, TG, JM, JP, IS, RS, LNT, DAH, PRM, KR, KW are trial co-applicants and members of the Trial Management Group. LD and GMF supported management of the trial. All authors read and approved the final manuscript.

Competing interests

NP, JP, BC, RF, TG, RS, LNT, KW, DAH, PRM, KR, AB, PFV, GMF, LD, MP are funded by the UK NIHR HTA research programme
KR is the Director of NIHR Health and Social Care Delivery Research (HSDR) Programme.
NP and LNT is a member of the NIHR HTA research prioritisation panel and LNT is a member of the NIHR HTA funding panel.

For peer review only - http://bmjopen.bmj.com/site/about/guidelines.xhtml

JM is Chair of SuDDICU Australia Management Committee, Director of The George Institute for Global Health and Leadership Fellowship of National Health and Medical Research Council, Australia.

Funding

This work was supported by NIHR HTA Programme (reference: 16/152/01). The views expressed are those of the author(s) and not necessarily those of the NIHR or the Department of Health and Social Care nor the Sponsor. The funder and sponsor had no role in the writing of this article of the decision to submit the protocol for publication. Cambridge University Hospitals NHS Foundation Trust and The University of Cambridge is the sponsor.

Acknowledgments

Our thanks to the research and clinical teams at the participating sites, and the support of the Biomedical Research Centres at the sites: Cambridge University Hospitals NHS Foundation Trust, Birmingham Women and Children's Hospital (Birmingham), Bristol Royal Hospital for Children (Bristol), John Radcliffe Hospital (Oxford), Southampton Children's Hospital (Southampton), St. George's Hospital (London).

REFERENCES

1. Patrick SW, Kawai AT, Kleinman K, et al. Health care-associated infections among critically ill children in the US, 2007-2012. Pediatrics 2014;134(4):705-12. doi: 10.1542/peds.2014-0613
2. Field-Ridley A, Dharmar M, Steinhorn D, et al. ICU-Acquired Weakness Is Associated With Differences in Clinical Outcomes in Critically Ill Children. Pediatr Crit Care Med 2016;17(1):53-57. doi:10.1097/PCC.0000000000000538
3. Choong K, Al-Harbi S, Siu K, et al. Functional recovery following critical illness in children: the "wee-cover" pilot study. *Pediatr Crit Care Med* 2015;16(4):310-8. doi: 10.1097/pcc.0000000000000362
4. Heyland DK, Cook DJ, Griffith L, et al. The attributable morbidity and mortality of ventilator-associated pneumonia in the critically ill patient. The Canadian Critical Trials Group. *Am J Respir Crit Care Med* 1999;159(4 Pt 1):1249-56. doi: 10.1164/ajrccm.159.4.9807050
5. Bekaert M, Timsit J-F, Vansteelandt S, et al. Attributable Mortality of Ventilator-Associated Pneumonia. *American Journal of Respiratory and Critical Care Medicine* 2011;184(10):1133-39. doi: 10.1164/rccm.201105-0867OC
6. Francis J, Duncan E, Prior M, et al. Selective decontamination of the digestive tract in critically ill patients treated in intensive care units: A mixed-methods feasibility study

For peer review only - http://bmjopen.bmj.com/site/about/guidelines.xhtml

(the SuDDICU study). *Health technology assessment (Winchester, England)* 2014;18:1-170. doi: 10.3310/hta18250

7. de Smet AMGA, Kluytmans JAJW, Cooper BS, et al. Decontamination of the Digestive Tract and Oropharynx in ICU Patients. *New England Journal of Medicine* 2009;360(1):20-31. doi: 10.1056/NEJMoa0800394
8. Canter RR, Harvey SE, Harrison DA, et al. Observational study of current use of selective decontamination of the digestive tract in UK critical care units. *Br J Anaesth* 2014;113(4):610-7. doi: 10.1093/bja/aeu108
9. Daneman N, Sarwar S, Fowler RA, et al. Effect of selective decontamination on antimicrobial resistance in intensive care units: a systematic review and meta-analysis. *Lancet Infect Dis* 2013;13(4):328-41. doi: 10.1016/s1473-3099(12)70322-5
10. Liberati A, D'Amico R, Pifferi, et al. Antibiotic prophylaxis to reduce respiratory tract infections and mortality in adults receiving intensive care. *Cochrane Database Syst Rev* 2004(1):Cd000022. doi: 10.1002/14651858.CD000022.pub2
11. Oostdijk EAN, Smet AMGAd, Blok HEM, et al. Ecological Effects of Selective Decontamination on Resistant Gram-negative Bacterial Colonization. *American Journal of Respiratory and Critical Care Medicine* 2010;181(5):452-57. doi: 10.1164/rccm.200908-1210OC
12. Plantinga NL, Bonten MJ. Selective decontamination and antibiotic resistance in ICUs. *Crit Care* 2015;19(1):259. doi: 10.1186/s13054-015-0967-9
13. Price RJ, Cuthbertson BH, collaboration obotS. Selective decontamination of the digestive tract and oropharynx: after 30 years of debate is the definitive answer in sight? *Current Opinion in Critical Care* 2016;22(2):161-66. doi: 10.1097/mcc.0000000000000281
14. Wittekamp BH, Plantinga NL, Cooper BS, et al. Decontamination Strategies and Bloodstream Infections With Antibiotic-Resistant Microorganisms in Ventilated Patients: A Randomized Clinical Trial. *JAMA* 2018;320(20):2087-98. doi: 10.1001/jama.2018.13765
15. Billot L, Cuthbertson BH, et al. Protocol summary and statistical analysis plan for the Selective Decontamination of the Digestive Tract in Intensive Care Unit Patients (SuDDICU) crossover, cluster randomised controlled trial. *Critical Care and Resuscitation* 2021
16. Petros A, Silvestri L, Booth R, et al. Selective decontamination of the digestive tract in critically ill children: systematic review and meta-analysis. Pediatr Crit Care Med 2013;14(1):89-97. doi: 10.1097/PCC.0b013e3182417871
17. Woolfall K, Young B, Frith L, et al. Doing challenging research studies in a patient-centred way: a qualitative study to inform a randomised controlled trial in the paediatric

For peer review only - http://bmjopen.bmj.com/site/about/guidelines.xhtml

emergency care setting. *BMJ Open* 2014; 4(5). http://europepmc.org/abstract/MED/24833694

18. Weijer C, Grimshaw JM, Eccles MP, et al. The Ottawa Statement on the Ethical Design and Conduct of Cluster Randomized Trials. *PLoS Med* 2012;9(11):e1001346. doi: 10.1371/journal.pmed.1001346
19. Nix HP, Weijer C, Brehaut JC, et al. Informed consent in cluster randomised trials: a guide for the perplexed. *BMJ Open* 2021;11(9):e054213-e13. doi: 10.1136/bmjopen-2021-054213
20. Chan CL, Leyrat C, Eldridge SM. Quality of reporting of pilot and feasibility cluster randomised trials: a systematic review. *BMJ Open* 2017; 7(11). https://doi.org/10.1136/bmjopen-2017-016970
21. Malterud K, Siersma VD, Guassora AD. Sample Size in Qualitative Interview Studies: Guided by Information Power. *Qual Health Res* 2016;26(13):1753-60. doi: 10.1177/1049732315617444
22. Boyatzis R. Transforming Qualitative Information: Thematic Analysis and Code Development. 1998
23. Braun V, Clarke V. Using thematic analysis in psychology. *Qualitative Research in Psychology* 2006;3(2):77-101. doi: 10.1191/1478088706qp063oa
24. Deja E, Peters MJ, Khan I, et al. Establishing and augmenting views on the acceptability of a paediatric critical care randomised controlled trial (the FEVER trial): a mixed methods study. *BMJ Open* 2021;11(3):e041952-e52.
25. Sekhon M, Cartwright M, Francis JJ. Acceptability of healthcare interventions: an overview of reviews and development of a theoretical framework. *BMC Health Serv Res* 2017;17(1):88. doi: 10.1186/s12913-017-2031-8
26. Glaser BG. The Constant Comparative Method of Qualitative Analysis*. Social Problems 1965;12(4):436-45. doi: 10.2307/798843
27. Medical Research Council Good research practice: principles and guidelines, 2012. Available: https://mrc.ukri.org/publications/browse/good-research-practice-principles-and-guidelines/
28. Academic. *International conference on harmonisation of technical requirements for registration of pharmaceuticals for human use. guideline for good clinical practice*, 1996.
29. Health Research Authority. UK policy framework for health and social care research, 2017. Available: https://www.hra.nhs.uk/planning-and-improving-research/policies-standards-legislation/uk-policy-framework-health-social-care-research/

For peer review only - http://bmjopen.bmj.com/site/about/guidelines.xhtml

Figure legends

Figure 1. Trial Design

Figure 2. Trial Schema of eligibility, site randomisation, study intervention, ecology surveillance, embedded mixed-methods study, follow up.

For peer review only

For peer review only - http://bmjopen.bmj.com/site/about/guidelines.xhtml

Supplementary Files

Supplementary file 1 for protocol paper including:

Supplementary Table 1: SDD suspension dosing

Supplementary Table 2: Composition and Characteristics of SDD paste

Supplementary Table 3: Composition and Characteristics of SDD suspension

Supplementary Table 4: General Investigational drug product information (SDD paste)

Supplementary Table 5: General Investigational drug product information (SDD suspension)

Appendices 1: Verita Pharma most recent GMP Licence

Appendices 2: SDD Oral Paste 5°C R&D stability summary

Appendices 3: SDD Oral Paste 25°C-60%RH R&D stability

Appendices 4: SDD Powder for Suspension 5°C R&D stability

Appendices 5: SDD Powder for Suspension 25°C-60%RH R&D stability

Appendices 6: SDD Suspension 5°C R&D stability

Appendices 7: SDD Suspension NF 25°C-60%RH

Appendices 8: SyrSpend SF pH4 Dry – 30% reductio in SDD Powder for Suspension Formulation

Appendices 9: PICnIC Labels (Boz, SDD oral paste, SDD suspension)

Supplementary file 2

Ecological Poster_v1.2, 06 October 2021

PICnIC Consent Form Parent or Legal Guardian (non bereaved)_v3.1, 25 September 2021

PICnIC Consent Form Parent or Legal Guardian (bereaved)_v1.1, 10 September 2021

For peer review only - http://bmjopen.bmj.com/site/about/guidelines.xhtml

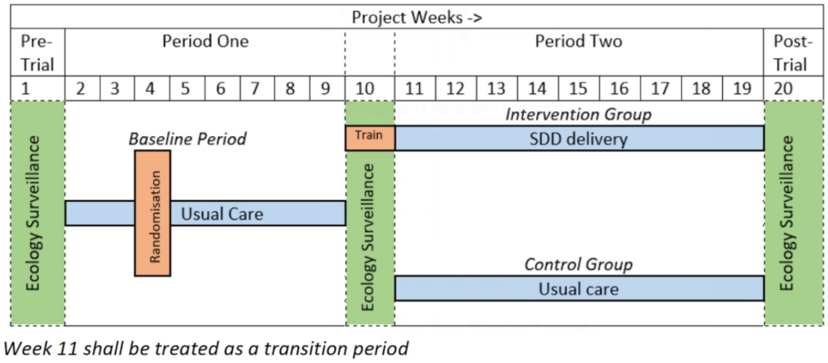

Figure 1. Trial Design

89x39mm (600 x 600 DPI)

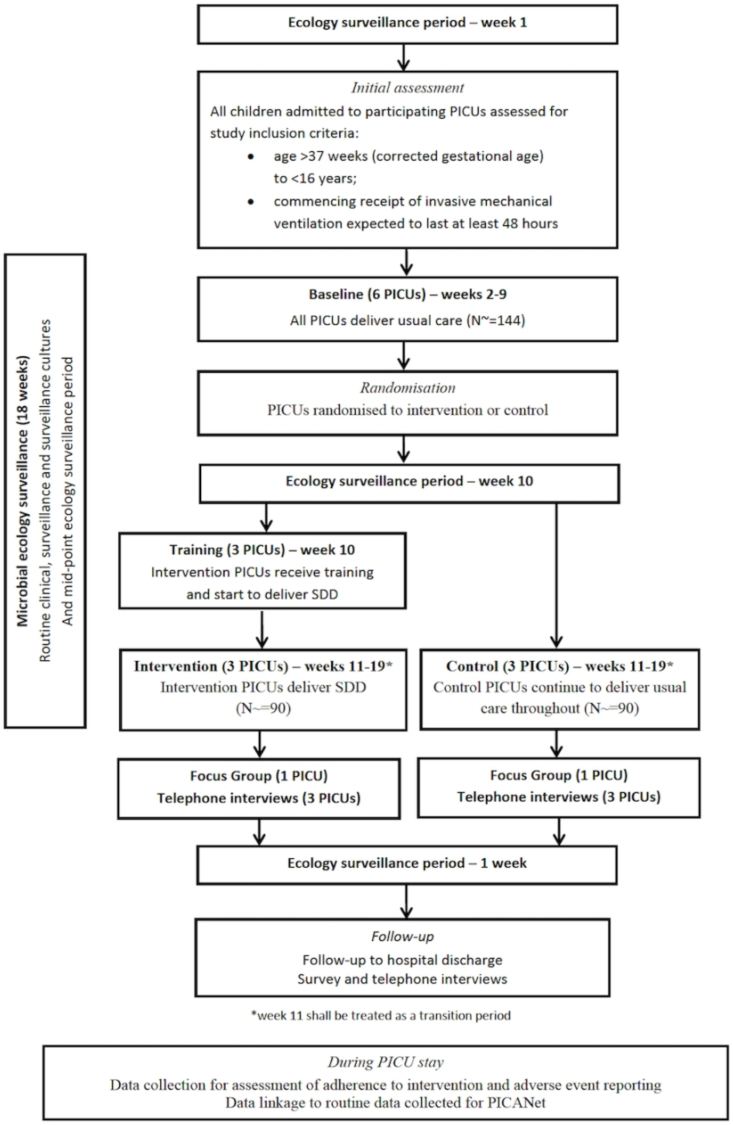

Figure 2. Trial Schema of eligibility, site randomisation, study intervention, ecology surveillance, embedded mixed-methods study, follow up.

90x128mm (300 x 300 DPI)

Supplementary file 1 for protocol paper:

The use of selective gut decontamination in critically ill children: Paediatric Care and Infection Control (PICnIC): A protocol of a pilot cluster randomised trial

How does SDD work?

SDD paste and SDD suspension are both formulations containing three non-absorbable antibiotics and antifungals: colistin (polymyxin E), tobramycin and nystatin. All three are licensed antibacterial and antifungal drugs, and are currently used in critically ill patients when indicated.

In SDD therapy, the drugs are used to eradicate the gastro-intestinal carriage of potentially pathogenic micro-organisms including Staphylococcus aureus, methicillin-resistant Staphylococcus aureus (MRSA), aerobic Gram-negative bacilli and yeasts.[1]

The drugs are non-absorbable, ensuring that their concentrations remain high enough in the mouth and into the colon to selectively eradicate the carriage of pathogenic micro-organisms, whilst not influencing the protective anaerobic flora, thereby decreasing colonisation resistance.[1]

Colistin is a multicomponent antibiotic. It is a mixture of several closely related decapeptides (polymyxin E). The main components are polymyxin E1 and E2. Colistin has an antimicrobial spectrum and mode of action similar to that of polymyxin B, but is slightly less active. It is bactericidal to most Gram-negative bacteria.[1]

Tobramycin sulphate is an aminoglycoside antibiotic with good aqueous solubility. It acts against many strains of Gram-negative bacteria, including pseudomonas aeruginosa.[1]

Colistin and tobramycin act in synergy against proteus and pseudomonas species, and offer the most potent anti-pseudomonal combination, with an effective clearance of pseudomonas from the gut. Both agents absorb endotoxin released by aerobic Gram-negative bacilli. This feature is important because endotoxin can be absorbed into the bloodstream from the gut of seriously ill patients. This contributes to fever, inflammatory activation, shock and organ failure.[1,2]

Emergence of resistance to colistin is rare. Although there are bacteria producing tobramycin-inactivating enzymes, colistin is thought to protect tobramycin from being destroyed by these enzymes. Tobramycin is intrinsically the most active aminoglycoside against pseudomonas, and is minimally inactivated by saliva and faeces.

In critically ill patients, the use of intravenous antimicrobials suppress the patient's commensal species in the gastrointestinal tract. This is associated with overgrowth of extended-spectrum beta-lactamase (ESBL)-producing aerobic Gram-negative bacilli in the gut. The action of enteral colistin and tobramycin against aerobic Gram-negative bacilli prevents the persistence of ESBL producing aerobic Gram-negative bacilli.[3]

Nystatin is a non-absorbable polyene drug with wide antifungal activity, especially against candida species. It significantly reduces fungal carriage and overall fungal infections and is less likely to promote the emergence of resistant candidal strains compared to other antifungal agents.[4] It also has advantages of low cost and absence of side effects.[4]

The most widely used SDD regimen to date uses amphotericin B in combination with polymyxin (colistin) and tobramycin. For PICnIC, nystatin is used in place of amphotericin B

due to difficulties sourcing amphotericin B. Nystatin, like amphotericin B is a non-absorbable polyene with wide antifungal activity.[4]

How is SDD administered?

Every six hours a 'pea-sized' amount (0.5 g) of SDD paste will be topically applied to the buccal mucosa and oropharynx and an SDD suspension is administered to the gastrointestinal tract via the most proximal feeding tube, with dosing according to age (Supplementary Table 1):

Supplementary Table 1: SDD suspension dosing

	0 – 4 years	5 – 12 years	≥13 years
**Polymyxin E (Colistin)**	25mg	50mg	100mg
**Tobramycin**	20mg	40mg	80mg
**Nystatin**	0.5 x 10^6 IU	1 x 10^6 IU	2 x 10^6 IU
	**2.5ml**	**5ml**	**10ml**

Similarity to other compounds

The most widely used SDD regimen to date is a combination of polymyxin E (colistin), tobramycin and amphotericin B applied as an oral paste and suspension to treat both the throat and gut, respectively.

For PICNIC, nystatin will be used in place of amphotericin B due to difficulties sourcing amphotericin B. Nystatin, like amphotericin B is a non-absorbable polyene with wide antifungal activity.[5] Nystatin may prevent the emergence of resistant fungal strains such as candida species and has advantages such as its low cost and absence of side effects.

Chemistry, Manufacturing and controls.

The SDD paste and suspension has been manufactured in accordance with Good Laboratory Practice (GLP) and Good Manufacturing Practice (GMP) for clinical studies (See Appendix 1 for Verita Pharma GMP licence).

Development plan

An unpublished, inception cohort pilot study testing inclusion criteria and examining process measures and outcomes was conducted in an adult population by collaborators at The George Institute and informed the development of the PICnIC protocol. The outcomes of this study will inform the development of a full trial within paediatrics.

Physical, Chemical and pharmaceutical properties and formulation.

Study Drugs.

The active pharmaceutical ingredients of both the SSD paste (Supplementary Table 2) and SDD suspension (Supplementary Table 3) are colistin sulphate, tobramycin sulphate and nystatin.

For peer review only

Supplementary Table 2: Composition and Characteristics of SDD paste.

Name	SDD paste
International nomenclature	The paste is a semi-solid dosage forms containing finely dispersed solids, have a stiff consistency, and is intended for topical application.<br>Active pharmaceutical ingredients:<br>Colistin sulphate<br>Tobramycin (as sulphate)<br>Nystatin
Sponsor name	The George Institute for Global Health
Chemical abstract service number	Colistin : 1264-72-8<br>Tobramycin: 49842-07-1<br>Nystatin: 1400-61-9
Chemical structure	Colistin Sulphate 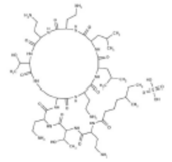<br>Tobramycin 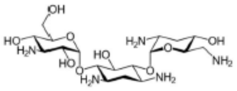 Nystatin 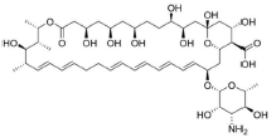
Molecular formula	Colistin : $2(C_{52}H_{98}N_{16}O_{13}).5(H_2SO_4)$<br>Tobramycin: $C_{18}H_{37}N_5O_9$<br>Nystatin: $C_{47}H_{75}NO_{17}$
Molecular weight	Colistin: 2801 g/mol<br>Tobramycin: 467.5 g/mol<br>Nystatin: 926.1 g/mol
Description	SDD paste is a mixture of antimicrobial powders – colistin sulphate, tobramycin sulphate and nystatin- with mineral oil light, petrolatum white and methocel E4M premium in an oral syringe.
Odour	Not applicable
Solubility	Not applicable
Properties	A smooth, yellow paste of uniform consistency

Supplementary Table 3: Composition and characteristics of SDD suspension.

Name	SDD suspension
International nomenclature	The suspensions is liquid preparations containing drug substance(s) and consist of solid particles dispersed throughout a liquid phase in which the particles are present in excess of the solubility. Some suspensions are prepared and ready for use, while others are solid mixtures intended for constitution before use with an appropriate vehicle.
Sponsor name	As per SDD paste
Chemical abstract service number	As per SDD paste
Structure	As per SDD paste
Molecular formula	As per SDD paste
Molecular weight	As per SDD paste
Description	SDD powder for suspension is a blend of antimicrobial powders- colistin sulphate, tobramycin sulphate and nystatin with a suspending agent (syrspend pH 4 dry), a preservative (potassium sorbate) and citric acid monohydrate in a bottle. Before use, each bottle is reconstituted with purified/distilled water to the required volume and then shaken.
Odour	Not applicable
Solubility	Part of the powder will be dissolved and part suspended
Properties	SDD suspension; after suspending: an opaque, straw coloured liquid

Manufacture.
The SDD paste and SDD suspension are manufactured through a series of proprietary processing steps and performed in accordance with GLP/GMP under licence at:
Verita Pharma Pty LTD
Unit 3, 4 Endeavour Rd
Taren Point
NSW 2229
Australia

Analysis and characterisation of study drug.
The identity of each container within each batch of raw material destined for study drug is confirmed by full pharmacopoeia analysis by validated high-performance liquid chromatography (HPLC) and microbiology methodology (in accordance with annex 8 of Pharmaceutical Inspection Co-operation Scheme guide to GMP manufacturing).

Homogeneity and potency of each finished product batch of investigational product is confirmed via similar methodology.

Impurities will be assessed by Verita Pharma using HPLC and other analytical methods during on-going stability studies.

Stability
The manufacturer stability testing program is focused on determining allowable shelf-life and ensuring the product maintains compliance with the claims made on the SDD paste and SDD suspension labels when exposed to routine usage and storage conditions.

Stability testing uses HPLC and microbiology analyses to ensure that study drugs remains homogenous, with potency within specifications and exhibits normally expected physical

review only

characteristics, when stored for defined durations, at the ranges of temperature and subjected to reasonably expected temperature excursions.

Investigational products

Formulation.

SDD paste

The clinical products are formulated by combining the ingredients shown in Supplementary Table 4 using a series of proprietary processing steps prior dispensing into 1ml oral syringes. SDD paste is formulated to contain per 0.5g paste: colistin sulphate 10 mg tobramycin 10 mg and nystatin 125,000 IU.

Supplementary Table 4: General Investigational drug product information.

Ingredient	Specification	Purpose	Conc. (per gram)
Colistin sulphate	BP	Active	20mg
Nystatin	Eur. Ph. 8th ed. (BP)	Active	0.25mu
Tobramycin (as sulphate)	USP	Active	20mg
Mineral oil light	USP	Excipient	50mg
Methocel e4m premium	USP	Excipient	177mg
Petrolatum white	USP	Excipient	686mg*

BP = British Pharmacopoeia provides quality standards for UK pharmaceutical substances and medicinal products
Eur.Ph.8th ed.= European Pharmacopoeia 8th Edition is Europe's scientific and legal benchmark for pharmacopoeia standards
USP = United States Pharmacopeia has established standards for manufacturing and supplying drugs worldwide
Active = an active component in a medicines final formulation
Excipient = an excipient is an inactive substance for the purpose of bulking-up drug formulations
Methocel e4m premium = a renewable raw material, are water soluble polymer derided from cellulose
Petrolatum white = semi-solid mixture of hydrocarbons.
* For 'petrolatum white' quantity added to batch is calculated to quantum satis

SDD suspension

The clinical products are formulated by combining the ingredients shown in Supplementary Table 5 using a series of proprietary processing steps and dispensing into defined aliquots within each polyethylene terephthalate (PET) bottle, for storage. When the product is required for the trial, each bottle of antimicrobial powder is reconstituted to the desired volume with purified/distilled water. The formulation includes a suspending agent (SyrSpend sf ph4) to ensure that when the bottle is standing, the active powder (Nystatin) is evenly dispersed after shaking between administrations to the patient, thereby preventing 'caking' on the bottom of the bottle. Due to the difficulties experienced at participating sites in achieving consistent reconstitution and the increased viscosity (thickening) of the suspension after 3 days, the formulation was modified in September 2018 to reduce the amount of SyrSpend by 30%. The removal of 30% suspending agent has no influence on product efficacy, chemical attributes or on the active components.

Supplementary Table 5: General investigational drug product information

Ingredient	Specification	Purpose	Concentration After reconstitution per ml
Colistin sulphate	BP	Active	10mg
Nystatin	Eur. Ph. 8th ed. (BP)	Active	0.20mu
Tobramycin (as sulphate)	USP	Active	8mg
Syrspend sf ph4	USP	Excipient	45.5mg
Citric acid monohydrate	BP	Excipient	2.86mg
Potassium sorbate	USP	Preservative	2.0mg

BP = British Pharmacopoeia provides quality standards for UK pharmaceutical substances and medicinal products
Eur.Ph.8th ed. = European Pharmacopoeia 8th Edition is Europe's scientific and legal benchmark for pharmacopoeia standards
USP = United States Pharmacopeia has established standards for manufacturing and supplying drugs worldwide
Active = an active component in a medicines final formulation
Excipient = an excipient is an inactive substance for the purpose of bulking-up drug formulations
Preservative = a natural or synthetic chemical that prevents decomposition by microbial growth or by undesirable chemical changes
Syrspend sf ph4 = is a ready-to-use, all-in-one suspending and sweetening oral liquid
Citric acid monohydrate = a tricarboxylic acid and maintains stability of active ingredients
Potassium sorbate = is the potassium salt of sorbic acid, commonly used preservative in the conservation of liquid pharmaceutical preparations.

Final dosage form and presentation
SDD paste (0.5g) is supplied in 1ml BD oral syringe and is stored at 2-8°C. During patient use the paste can be stored in a temperature-controlled room of less than 25°C for up to one week. SDD suspension is supplied in antimicrobial powder form in a sealed PET bottle with a syringe filling adaptor under a child resistant screwcap and is stored at 2-8°C. After reconstitution of the powder, the suspension can be stored in a temperature-controlled room of less than 25°C for up to one week.

Container and packaging
Each SDD study drug kit contains, twenty 1ml BD oral syringes SDD paste, a single sealed PET bottle of SDD suspension (powder form) and a syringe filling adaptor and shipped under refrigerated/cold chain 2-8˚C (35°–46°F) conditions to the clinical trial site.

Storage and handling
The SDD study drug kits are to be stored at 2–8°C (35°–46°F) in a secure area. When SDD powder is reconstituted into suspension the drug kit (paste and suspension) can be stored in a temperature controlled room of less than 25°C for up to 1 week. Each pre-filled syringe is to be discarded after use (i.e. single use only). The PET bottle is to be returned to the SDD study drug kit after each dose. Hospitals will need to provide their own 10ml oral/enteral syringe to access and measure each dose from the PET bottle (which contains a syringe filling adaptor).

Shelf life
There is no information in the literature regarding stability testing of drugs containing colistin sulphate, tobramycin sulphate and nystatin as this is a new formulation. In reference literature, data concerning stability testing of drugs containing polymyxin/tobramycin/amphotericin - the most widely used SDD regimen to date – is limited.

review only

Stability determinations/concentrations have therefore been assessed by HPLC and microbiology methods. Test methods have been developed and validated, and real-time ageing tests conducted to define safe storage parameters, including reasonably expected excursions.

The stability program for the SDD paste and SDD suspension is continually ongoing. The expiry date on the label will reflect the most recent shelf-life determination of the product contained within the syringe/bottle.
Current stability testing has demonstrated that:

1. SDD paste is stable at 2-8°C for up to 12 months.
2. SDD suspension is stable at 2-8°C for up to 2 weeks.
3. SDD powder for suspension is stable at 2-8°C for up to 12 months.
4. SDD paste, SDD powder for suspension and SDD suspension can be stored below 25°C for short excursions up to one week. (There is stability evidence up to one month, but this is not appropriate for the suspension because it is 'in use' at this stage. Suspension should be used as per the protocol once reconstituted.)

Refer to Appendices 2-8 for current stability results for SDD paste, SDD powder and SDD suspension.

For peer review only

Reference list

1. van Saene HKF, Silvestri L, de la Cal MA, Gullo A, editors. Infection Control in the Intensive Care Unit [Internet]. Milano: Springer Milan; 2012 [cited 2018 Oct 26]. Available from: http://link.springer.com/10.1007/978-88-470-1601-9
2. Pathan N, Burmester M, Adamovic T, Berk M, Ng KW, Betts H, et al. Intestinal injury and endotoxemia in children undergoing surgery for congenital heart disease. Am J Respir Crit Care Med [Internet]. 2011;184(11):1261–9. Available from: http://www.ncbi.nlm.nih.gov/pubmed/21868501
3. van der Spoel HI, Gerritsen RT. SDD for the Prevention and Control of Outbreaks. Selective Digestive Tract Decontamination in Intensive Care Medicine: a Practical Guide to Controlling Infection [Internet]. Milano: Springer Milan; 2008 [cited 2018 Oct 26]. p. 141–54. Available from: http://link.springer.com/10.1007/978-88-470-0653-9_11
4. Normand S, François B, Dardé M-L, Bouteille B, Bonnivard M, Preux P-M, et al. Oral nystatin prophylaxis of Candida spp. colonization in ventilated critically ill patients. Intensive Care Med [Internet]. 2005 Nov 30 [cited 2018 Oct 26];31(11):1508–13. Available from: http://www.ncbi.nlm.nih.gov/pubmed/16195905
5. Ruza F, Alvarado F, Herruzo R, Delgado MA, García S, Dorao P, et al. Prevention of nosocomial infection in a pediatric intensive care unit (PICU) through the use of selective digestive decontamination. Eur J Epidemiol [Internet]. 1998 Oct [cited 2018 Oct 25];14(7):719–27. Available from: http://www.ncbi.nlm.nih.gov/pubmed/9849834

For peer review only

Appendices

1. Verita Pharma most recent GMP Licence-MI-2016-LI-07350, dated 23 July 2020

Australian Government
Department of Health
Therapeutic Goods Administration

Licence to Manufacture Therapeutic Goods – Part 1

Licence Number:
MI-2016-LI-07350-1

Granted to:
Verita Pharma Pty Ltd
ABN: 89 604 520 532

Manufacturing Site Address:
Lot 8, 10 and 11 (SP92206), 2 Endeavour Road
Caringbah NSW 2229

The manufacturer above is hereby authorised under Section 38 of the *Therapeutic Goods Act 1989* to carry out the following steps in the manufacture of therapeutic goods at the manufacturing site address specified above.

Manufacturing Type	Sterility	Dosage Form	Product Category	Manufacturing Step
Medicine manufacture	Non Sterile	Semi Solids	Therapeutic Goods for Clinical Trials	Finished Product Manufacture
Medicine manufacture	Non Sterile	Semi Solids	Registered Therapeutic Good	Finished Product Manufacture
Medicine manufacture	Non Sterile	Powders Group	Therapeutic Goods for Clinical Trials	Finished Product Manufacture
Medicine manufacture	Non Sterile	Powders Group	Registered Therapeutic Good	Finished Product Manufacture
Medicine manufacture	Non Sterile	Oral Liquid, suspension	Therapeutic Goods for Clinical Trials	Finished Product Manufacture
Medicine manufacture	Non Sterile	Oil	Therapeutic Goods for Clinical Trials	Finished Product Manufacture
Medicine manufacture	Non Sterile	Spray	Therapeutic Goods for Clinical Trials	Finished Product Manufacture
Medicine manufacture	Sterile & Non Sterile	All Dosage Forms	Not Applicable	Testing chemical and physical

This Licence is the property of the Therapeutic Goods Administration and must be returned or destroyed upon demand.
This Licence remains valid until otherwise suspended or revoked by the Therapeutic Goods Administration.
The status of an Australian Licence may be viewed at https://www.ebs.tga.gov.au/

PO Box 100 Woden ACT 2606 ABN 40 939 406 804
Phone: 1800 020 653 Fax: 02 6203 1605 Email: info@tga.gov.au www.tga.gov.au

TGA Health Safety Regulation

Page 1 of 2

Australian Government
Department of Health
Therapeutic Goods Administration

Licence to Manufacture Therapeutic Goods – Part 1

Licence Number:
MI-2016-LI-07350-1

Manufacturing Type	Sterility	Dosage Form	Product Category	Manufacturing Step
Medicine manufacture	Sterile	Injections	Compounded Medicine	Sterile Finished Product Manufacture
Medicine manufacture	Sterile	Injections	Therapeutic Goods for Clinical Trials	Sterile Finished Product Manufacture

This licence is subject to the requirements of the *Therapeutic Goods Act1989*, and its Regulations.

Section 40(4) of the *Therapeutic Goods Act 1989* and Regulation 19, 20, 21 and 22 of the Therapeutic Goods Regulations 1990 impose various statutory conditions on all licences to manufacture therapeutic goods.

In addition to that, the specific conditions mentioned in Part 2 of this licence have been imposed under Section 40(1) or 40(2) of the *Therapeutic Goods Act 1989*.

Originally Granted: **16 May 2017** Date Revised: **23 July 2020**

This Licence is the property of the Therapeutic Goods Administration and must be returned or destroyed upon demand.
This Licence remains valid until otherwise suspended or revoked by the Therapeutic Goods Administration.
The status of an Australian Licence may be viewed at https://www.ebs.tga.gov.au/

PO Box 100 Woden ACT 2606 ABN 40 939 406 804
Phone: 1800 020 653 Fax: 02 6203 1605 Email: info@tga.gov.au www.tga.gov.au

TGA Health Safety Regulation

Page 2 of 2

2. SDD Oral Paste 5°C R&D Stability Summary

Form No: TF2402-01

VERITA PHARMA

R&D Stability Report

Product Name: **SDD Oral Paste**	Product Code: **FP002**	Company Name: **The George Institute**
Dosage Form: **Paste**	Batch No: **241117**	Stability Study: **SP-0001-03**
Packaging: **BD oral/enteral 1 ml syringe and tip cap**	Storage Temperature: **5°C ± 3°C**	Humidity: **N/A**

Time points		Initial	2 w	4 w	12 w	16 w	6 M	9 M	12M
Due date		27/11/17	11/12/17	08/01/18	19/02/18	19/03/18	27/05/18	27/08/18	27/11/18
Test	Specifications								
Appearance	Light yellow paste	complies	complies	complies	complies	complies	complies		complies
Viscosity	TBD	128,000 cps	157,000 cps	158,000 cps	150,333 cps	198,000 cps	198,000 cps		180,000 cps
Nystatin Assay	250,000 IU/g<br>*Assay precision fiducial limits: 96-106%*<br>*UFL: NLT 90% of stated IU*<br>*LFL: NMT 115 % of stated IU*	243,621 IU/g	*Not available (*)*	242,806 IU/g	243,996 IU/g	244,270 IU/g	249,698 IU/g		245,342 IU/g
Colistin Sulphate Assay	20 mg/g<br>*Assay precision fiducial limits: 96-106%*<br>*UFL: NLT 97% of stated mg*<br>*LFL: NMT 110 % of stated mg*	20.1 mg/g	*Not available (*)*	19.5 mg/g	19.6 mg/g	19.7 mg/g	19.9 mg/g	Cancelled	20.0 mg/g
Tobramycin Assay	20 mg/g<br>*18 mg/g – 24 mg/g*	20.24 mg/g	21.0 mg/g	20.4 mg/g	20.56 mg/g	21.20 mg/g	21.65 mg/g		20.20 mg/g
Microbiological quality	Complies<br>TAMC: 10^2 CFU/g<br>TYMC: 10^1 CFU/g<br>*S aureus: Absence/g*<br>*P aeruginosa: Absence/g*	Complies			complies	complies			complies
PET	Complies								Complies USP
Date completed		19/12/17	28/12/17	17/01/18	08/03/18	10/04/18	14/06/18		05/04/2019

Comments: *The tests with (*) have not been performed.*

Checked By:______________________ (Sign and Date) **Authorised By QA Manager:**________________________ (Sign and Date)

ew only

3. SDD Oral Paste 25°C-60%RH R&D Stability

Form No: TF2402-01

VERITA PHARMA

R&D Stability Report

Product Name: **SDD Oral Paste**	Product Code: **FP002**	Company Name: **The George Institute**
Dosage Form: **Paste**	Batch No: **241117**	Stability Study: **SP-0001-03**
Packaging: **BD oral/enteral 1 ml syringe and tip cap**	Storage Temperature: **25°C ± 2°C**	Humidity: **60 %RH ± 5% RH**

Time points		Initial	8 weeks	12 weeks					
Due date		27/11/17	22/01/18	19/02/18					
Test	Specifications								
Appearance	Light yellow paste	complies	complies	complies					
Viscosity	TBD	128,000 cps	124,000 cps	123,000 cps					
Nystatin Assay	250,000 IU/g<br>*Assay precision fiducial limits: 96-105%*<br>*UFL: NLT 90% of stated IU*<br>*LFL: NMT 115 % of stated IU*	243,621 UI/g	241.968 UI/g	241.161 UI/g					
Colistin Sulphate Assay	20 mg/g<br>*Assay precision fiducial limits: 96-105%*<br>*UFL: NLT 97% of stated mg*<br>*LFL: NMT 110 % of stated mg*	20.1 mg/g	19.8 mg/g	19.7 mg/g					
Tobramycin Assay	20 mg/g<br>*16 mg/g – 24 mg/g*	20.24 mg/g	20.40 mg/g	20.87 mg/g					
Microbiological quality	Complies<br>TAMC: 10^{3} CFU/g<br>TYMC: 10^{1} CFU/g<br>*S aureus*: Absence/g<br>*P aeruginosa*: Absence/g	Complies		Complies					
PET	Complies USP requirements		Complies						
Date completed		19/12/17	08/03/18	08/03/18					

Comments: ______________________________

Checked By:______________________ (Sign and Date) **Authorised By QA Manager:**________________________ (Sign and Date)

only

4. SDD Powder for Suspension 5°C R&D Stability

Form No: TF2402-01

VERITA PHARMA

R&D Stability Report

Product Name: **SDD Powder for Suspension**	Product Code: **FP001**	Company Name: **The George Institute**
Dosage Form: **Powder**	Batch No: **Int001071117**	Stability Study: **SP-0002-05**
Packaging: **Amber Plastic bottle with Child Resistant Cap**	Storage Temperature: **5°C ± 3°C**	Humidity: **N/A**

Time points		Initial	2 w	4 w	9 w	12 w	18 w	6 M	9 M	12 M
Due date		15/11/17	29/11/17	13/12/17	17/01/18	07/02/18	21/03/18	15/05/18	15/08/18	15/11/18
**Test**	**Specifications**									
**Appearance**	pale yellow powder	complies	complies	complies	complies	complies	complies	complies		complies
**Loss on Drying**	TBD	5.63 %	4.98 %	6.47 %	5.19 %	5.72 %	5.14 %	6.52 %		5.75 %
**pH** *(after reconstitution)*	4.0 – 5.0	4.49	4.71	4.63	4.58	4.45	4.32	4.54		4.53
**Nystatin Assay** *(after reconstitution)*	200,000 IU/ml *Assay precision fiducial limits: 95-105%* *UFL: NLT 95% of stated IU* *LFL: NMT 120 % of stated IU*	206,182 IU/ml	200,288 IU/ml	*Not available (*)*	205.602 IU/ml	203,390 IU/ml	200,970 IU/ml	205,321 IU/ml		202,614 IU/ml
**Colistin Sulphate Assay** *(after reconstitution)*	10 mg/ml *Assay precision fiducial limits: 95-105%* *UFL: NLT 97% of stated mg* *LFL: NMT 110 % of stated mg*	10.2 mg/ml	9.8 mg/ml	*Not available (*)*	9.5 mg/ml	9.9 mg/ml	9.9 mg/ml	10.2 mg/ml	Cancelled	10.1 mg/ml
**Tobramycin Assay** *(after reconstitution)*	8 mg/ml *7.2 mg/g –9.6 mg/g*	8.73 mg/ml	8.87 mg/ml	8.84mg/ml	8.66 mg/ml	8.85 mg/ml	8.18 mg/ml	8.75 mg/ml		8.09 mg/ml
**Microbiological quality** *(after reconstitution)*	Complies TAMC: 10^2 CFU/g TYMC: 10^1 CFU/g *E.coli:* Absence/g	complies				complies	complies			complies
**PET** *(after reconstitution)*	Complies									complies
**Date completed**		05/12/17	22/12/17	02/01/18	19/01/18	08/03/18	12/04/18	13/06/18		29/01/2019

Comments: ***The tests with (*) have not been performed.***

Checked By:________________________ (Sign and Date) **Authorised By QA Manager:**__________________________ (Sign and Date)

5. SDD Powder for Suspension 25°C-60%RH R&D Stability

Form No: TF2402-01

VERITA PHARMA

R&D Stability Report

Product Name: **SDD Powder for Suspension**	Product Code: **FP001**	Company Name: **The George Institute**
Dosage Form: **Powder**	Batch No: **Int001071117**	Stability Study: **SP-0002-05**
Packaging: **Amber Plastic bottle with Child Resistant Cap**	Storage Temperature: **25°C ± 2°C**	Humidity: **60% RH**

**Time points**		**Initial**	**8 w**	**12 w**					
**Due date**		**15/11/17**	**10/01/18**	**07/02/18**					
**Test**	**Specifications**								
**Appearance**	pale yellow powder	**complies**	**complies**	**complies**					
**Loss on Drying**	TBD	**5.63**	**5.72**	**5.29**					
**pH** *(after reconstitution)*	4.0 – 5.0	**4.49**	**4.64**	**4.68**					
**Nystatin Assay** *(after reconstitution)*	200,000 IU/ml<br>*Assay precision fiducial limits: 95-105%*<br>*UFL: NLT 95% of stated IU*<br>*LFL: NMT 120 % of stated IU*	**206,182 IU/ml**	**203,811 IU/ml**	**199,917 IU/ml**					
**Colistin Sulphate Assay** *(after reconstitution)*	10 mg/ml<br>*Assay precision fiducial limits: 95-105%*<br>*UFL: NLT 97% of stated mg*<br>*LFL: NMT 110 % of stated mg*	**10.2 mg/ml**	**10.0 mg/ml**	**9.8 mg/ml**					
**Tobramycin Assay** *(after reconstitution)*	8 mg/ml<br>*7.2 mg/g –9.6 mg/g*	**8.73 mg/ml**	**8.65 mg/ml**	**8.57 mg/ml**					
**Microbiological quality** *(after reconstitution)*	Complies<br>TAMC: 10^2 CFU/g<br>TYMC: 10^1 CFU/g<br>*E.coli*: Absence/g	**complies**		**complies**					
**PET** *(after reconstitution)*	Complies		**Complies**						
**Date completed**		**05/12/17**	**08/03/18**	**08/03/18**					

Comments: ____

Checked By:______________________ (Sign and Date) **Authorised By QA Manager:**______________________ (Sign and Date)

6. SDD Suspension 5°C R&D Stability

Form No: TF2402-01

VERITA PHARMA

R&D Stability Report

Product Name: **SDD Suspension**	Product Code: **N/A**	Company Name: **The George Institute**
Dosage Form: **Suspension**	Batch No: **FP001071117**	Stability Study: **SP-0003-02**
Packaging: **Amber Plastic bottle with Child Resistant Cap**	Storage Temperature: **5°C ± 3°C**	Humidity: **N/A**

**Time points**		**Initial**	**1 w**	**2 w**	**3 w**	**1 M**	**1.5 M**	**2 M**	
**Due date**		**15/11/17**	**22/11/17**	**29/11/17**	**06/12/17**	**15/12/17**	**01/01/18**	**15/01/18**	
**Test**	**Specifications**								
**Appearance**	After shaking, a yellow liquid	**complies**	**complies**	**complies**	**complies**	**complies**	**complies**	**complies**	
**pH** *(after reconstitution)*	4.0 – 5.0	**4.50**	**4.47**	**4.54**	**4.47**	**4.61**	**4.45**	**4.55**	
**Nystatin Assay** *(after reconstitution)*	200,000 IU/ml *Assay precision fiducial limits: 95-105%* *UFL: NLT 95% of stated IU* *LFL: NMT 120 % of stated IU*	**206,182 IU/ml**	**205,268 IU/ml**	**201,465 IU/ml**	**204,480 IU/ml**	**204.835 IU/ml**	***Not available (*)***	**207,986 IU/ml**	
**Colistin Sulphate Assay** *(after reconstitution)*	10 mg/ml *Assay precision fiducial limits: 95-105%* *UFL: NLT 97% of stated mg* *LFL: NMT 110 % of stated mg*	**10.2 mg/ml**	**9.8 mg/ml**	**9.9 mg/ml**	**9.8 mg/ml**	**9.8 mg/ml**	***Not available (*)***	**10.1 mg/ml**	
**Tobramycin Assay** *(after reconstitution)*	8 mg/ml *7.2 mg/g –9.6 mg/g*	**8.68 mg/ml**	**8.77 mg/ml**	**8.77 mg/ml**	**8.57 mg/ml**	**8.79 mg/ml**	**8.72 mg/ml**	**8.44 mg/ml**	
**Microbiological quality** *(after reconstitution)*	Complies TAMC: 10^2 CFU/g TYMC: 10^1 CFU/g *E.coli*: Absence/g	**complies**						**complies**	
**PET** *(after reconstitution)*	Complies USP requirements							**complies**	
**Date completed**		**05/12/17**	**14/12/17**	**20/12/17**	**22/12/17**	**16/01/18**	**16/01/18**	**07/03/18**	

Comments: ***The tests with (*) have not been performed.***

Checked By:________________________ (Sign and Date) **Authorised By QA Manager:**__________________________ (Sign and Date)

ew only

7. SDD Suspension NF 25°C- 60%RH

VERITA PHARMA	Stability Report	Page 1 of 1

**Product Name:** **SDD Suspension NF**	**Product Code: N/A**	**Company Name: The George Institute**
**Dosage Form: suspension**	**Batch No: 1810005**	**Stability Study: SP-0006-01**
**Packaging: 240 ml amber plastic bottle with adapter and child resistant cap**	**Storage Temperature: 25°C ± 2°C**	**Humidity: 65 % RH ± 5 %RH**

**Time points**		**Initial**	**1 W**	**2 W**	**4 W**
**Due date**			29/01/19	01/02/19	18/02/19
**Test**	**Specifications**				
**Appearance**	After shaking, a yellow liquid	complies	complies	complies	complies
**pH**	4.0 – 5.0	4.58	4.50	4.59	4.72
**Nystatin Assay**	200,000 IU/ml<br>*Assay precision fiducial limits: 95-105%*<br>*UFL: NLT 95% of stated IU*<br>*LFL: NMT 120 % of stated IU*	204,141 IU/ml	199,838 IU/ml	200,830 IU/ml	202,077 IU/ml
**Colistin Sulphate Assay**	10 mg/ml<br>*Assay precision fiducial limits: 95-105%*<br>*UFL: NLT 97% of stated mg*<br>*LFL: NMT 110 % of stated mg*	10.4 mg/ml	10.0 mg/ml	9.7 mg/ml	9.7 mg/ml
**Tobramycin Assay**	8 mg/ml<br>*7.2 mg/g –9.6 mg/g*	7.81 mg/ml	8.53 mg/ml	7.97 mg/ml	8.00 mg/ml
**Microbiological quality**	Complies<br>TAMC: 10^2 CFU/g<br>TYMC: 10^1 CFU/g<br>*E.coli*: Absence/g	complies			complies
**PET**	Complies				complies
**Date completed**		08/11/2019	14/02/2019	20/02/2019	03/04/2019

Comments: ____

Checked By:______________________ (Sign and Date) **Authorised By QA Manager:**_______________________ (Sign and Date)

© Copyright - Verita Pharma
Information contained herein this document is the property of Verita Pharma - unauthorised disclosure to the public is prohibited.

8. SyrSpend SF pH4 Dry – 30% reduction in SDD Powder for Suspension Formulation

VERITA PHARMA

Syrspend SF pH4 Dry — 30% reduction in SDD Powder for Suspension Formulation

The current SDD Powder for Suspension contains 13.65g of Syrspend SF pH4 Dry (Syrspend SF), per bottle. Some sites currently part of the SuDDICU Clinical Trial, are finding some difficulty when reconstituting and using the suspension due to the thickness of the reconstituted product. The proposal is to reduce the quantity of Syrspend SF by 30% to 9.56g per bottle.

Syrspend SF is a starch based, preservative free suspending vehicle. The Syrspend SF contains; Modified Food

Starch (viscosity agent), Sucralose (sweetener) and a citrate/citric acid (buffer). The material allows the Active Pharmaceutical Ingredients (API's) to stay suspended and easy to (re)homogenise and is practically inert for chemical actions.

There are 3 API's in the SDD Suspension; Colistin Sulphate and Tobramycin Sulphate which are soluble in water and therefore dissolved in the suspension, and Nystatin which is suspended in the mixture and rehomogenised after shaking. The inert nature of the Syrspend SF means there should be no chemical interaction and therefore the efficacy of the drug remains the same. The microbiological stability of the product is maintained by the addition of Potassium Sorbate and Citric Acid to the powder blend. Both of these materials are dissolved in the water and the concentration of these materials will not change with the 30% reduction of Syrspend SF.

The reduction of the Syrspend SF quantity according to the supplier's procedure for use, falls within the recommended range of Water / Syrspend SF ratio.

On review with a quality consultant, Cathrine Dahlgren from CNQuality, it was concluded that the Syrspend SF reduction would not influence the activity of the 3 API's or the chemical and microbiological stability.

Based on the information reviewed it was determined that the current stability studies completed on the SDD Powder for Suspension and SDD Suspension (reconstituted) would be valid for the new formulation with a 30% reduction Syrspend SF. We are required to and will complete concurrent stability studies on the new formulation. The protocols have been written and are being approved.

Regards,

24 . 09 . 2018

Martina Bachmaier

General Manager

VERITA PHARMA PTY LTD ABN 89 604 520 532

UNIT 3/4 ENDEAVOUR ROAD CARINGBAH NSW 2229

PO BOX 2502 TAREN POINT NSW 2229

*612 8536 4130 | INFO@VERITAPHARMA.COM.AU I WWW.VERITAPHARMA.COM.AU

9. PICnIC Labels (Box, SDD oral paste, SDD suspension)

KEEP OUT OF REACH OF CHILDREN

FOR CLINICAL TRIAL USE ONLY

CHIEF INVESTIGATOR: Dr Nazima Pathan

SPONSOR: University of Cambridge & Cambridge University Hospitals NHS Trust
Addenbrookes Hospital, Hills Road, Cambridge
CB2 0QQ
Tel:01223 245151

PROTOCOL: PICnIC (REC 20/WM/0061)

BOX CONTAINS:

1 x Bottle of SDD Gastric Powder for Suspension (Total volume once reconstituted 210ml)

When reconstituted each 10ml contains: 2,000,000 units Nystatin, 100mg Colistin Sulfate, 80mg Tobramycin (as Sulfate)

20 x syringes of 500mg SDD Oral Paste

Each syringe contains: 10mg Colistin Sulfate, 10mg Tobramycin (as Sulfate) and 125,000 units Nystatin

Directions for use and reconstitution: Per the PICnIC protocol

Storage Conditions: Store at 2-8°C Do Not Freeze
In use: May be stored at up to 25°C for up to 5 days from date dispensed below.

FOR HOSPITAL USE:
Patient Trial Number:
Patient Full Name:
Date dispensed: ___/___/___
Use by: ____/____/____

Batch #:
Expiry:

Kit #

ITEM M-XXX-01

Sponsor:
University of Cambridge & Cambridge University Hospitals NHS Trust
Protocol: PICnIC (REC 20/WM/0061)

Keep out of reach of children

SDD ORAL PASTE 500mg

SINGLE USE ONLY

FOR CLINICAL TRIAL USE ONLY

Each syringe contains: 10mg Colistin Sulfate, 10mg Tobramycin (as Sulfate) and 125,000 units Nystatin

Batch: **Store at 2-8°C**
Expiry: **In use storage: <25°C up to 5 days**
Patient Trial No.:_______________

ITEM M-XXX-01

Keep out of reach of children

SDD GASTRIC POWDER FOR SUSPENSION (200ml)

SHAKE WELL BEFORE USE
FOR CLINICAL TRIAL USE ONLY
Protocol: PICnIC (REC 20/WM/0061)

BATCH:
EXPIRY:

Patient Trial No.: __________
ONCE RECONSTITUTED DISCARD AFTER:
___/___/____

Sponsor:
University of Cambridge & Cambridge University Hospitals NHS Trust

WHEN RECONSTITUTED EACH 10ml CONTAINS:
2,000,000 units Nystatin, 100mg Colistin Sulfate, 80mg Tobramycin (as Sulfate)

Store at 2-8°C
For oral use as directed by PICnIC protocol
Once reconstituted, may be stored at room temperature (up to 25°C) for up to 5 days.

ITEM M-XXX-01

We would like to ask your permission to take some samples from your child during their PICU stay to help improve our understanding of infection control

We are part of a study looking at how we can improve infection control procedures within the PICU. To help us understand if these measures work, we need to know what 'normal' looks like in the unit. To do this, we are looking at samples taken from children before, during and after the study.

This is the period (before/during/after) and we will be taking samples from children admitted between (date) and (date)

We would like to take samples from your child at regular timepoints throughout the admission. The samples won't hurt your child and we will make them as comfortable as possible. When we can, we will take them whilst carrying out routine care of your child.

We will take: nasopharyngeal (swab from nose and throat), stool samples or rectal swabs, and where clinically indicated urine and sputum samples and a swab of any wounds your child may have.

There is no direct benefit to your child, this work will help to improve treatment for children in the future. You do not have to agree to these samples and it will not affect your child's medical care. We will destroy any samples we have that are not needed by the doctors/nurses.

All samples are stored and analysed anonymously, it will not be possible to link them to your child. All research like this is reviewed by an independent group, called a Research Ethics Committee, who protect you and your child's interests. PICnIC was reviewed by West Midlands Black Country Health Research Authority who agree it is being conducted in a correct and appropriate matter.

If you would like more information, please speak to your child's bedside nurse, a member of the research team (name number) or the doctor in charge of the study (name number)

We are very grateful for your help - thank you

NHS
National Institute for Health Research

For peer review only - http://bmjopen.bmj.com/site/about/guidelines.xhtml

To be printed on local hospital headed paper

Paediatric Intensive Care and Infection Control (PICnIC) Study

Consent Form - Parent or Legal Guardian

Version 3.1, 25 September2020

To be completed by the Researcher:

Hospital name:	
PICnIC Study Number:	
Child's full name:	

To be completed by the Parent or Legal Guardian:

Once you have read and understood each statement – **if you agree, please write your initials in each box**

1. I confirm that I have read and understand the Participant Information Sheet (version (please insert) dated(please insert)) for the above study. I have had the opportunity to consider the information, ask questions and have had these answered satisfactorily. ☐
2. I understand that participation is voluntary, and that I am free to withdraw consent at any time, without giving any reason and without my child's medical care or legal rights being affected. ☐
3. I agree for additional samples from my child to be taken, analysed, and stored as part of this research study. ☐
4. I understand that relevant sections of my child's medical records and identifiable data collected during the study, held by the NHS, may be looked at by individuals from the Intensive Care National Audit & Research Centre (ICNARC) or regulatory authorities where it is relevant to my participation in this research. ☐
5. I agree to complete a questionnaire about my views on conducting a research study. ☐
6. I agree to be contacted for a telephone interview within the next 4 weeks. I understand that my contact details will be securely passed to the PICnIC study team at ICNARC and the University of Liverpool. ☐

Your signature:		Date:	
Your full name (PRINT):			
Researcher signature:		Date:	
Researcher full name (PRINT):			

Please sign and turn over to complete

For peer review only - http://bmjopen.bmj.com/site/about/guidelines.xhtml

Paediatric Intensive Care and Infection Control (PICnIC) Study

Parent or Legal Guardian contact information

To be completed by the Parent or Legal Guardian:

If you agree to be contacted for a telephone interview, please provide your details below:

**Telephone number:**	
**Mobile number:**	
**Email address:**	

1 copy for patient and parent/guardian; 1 copy for Investigator Site File; 1 copy to be kept with hospital notes

For peer review only

For peer review only - http://bmjopen.bmj.com/site/about/guidelines.xhtml

To be printed on local hospital headed paper

Paediatric Intensive Care and Infection Control (PICnIC) Study

Consent Form – Parent or Legal Guardian

Version 1.1, 10 September 2021

To be completed by the Researcher:

Hospital name:	
PICnIC Study Number:	
Child's full name:	

To be completed by the Parent or Legal Guardian:

Once you have read and understood each statement – **if you agree, please write your initials in each box**

1.	I confirm that I have read and understand the Participant Information Sheet (version (please insert) dated (please insert)) for the above study. I have had the opportunity to consider the information, ask questions and have had these answered satisfactorily.	☐
2.	I understand that participation is voluntary, and that I am free to withdraw consent at any time, without giving any reason and without my legal rights being affected.	☐
3.	I understand that relevant sections of my child's medical records and identifiable data collected during the study, held by the NHS, may be looked at by individuals from the Intensive Care National Audit & Research Centre (ICNARC) or regulatory authorities where it is relevant to my participation in this research.	☐
4.	I agree to be contacted for a telephone interview within the next two months. I understand that my contact details will be securely passed to the PICnIC study team at ICNARC and the University of Liverpool.	☐

Your signature:		Date:	
Your full name (PRINT):			
Researcher signature:		Date:	
Researcher full name (PRINT):			

Please sign and turn over to complete

For peer review only - http://bmjopen.bmj.com/site/about/guidelines.xhtml

Paediatric Intensive Care and Infection Control (PICnIC) Study

Parent or Legal Guardian contact information

To be completed by the Parent or Legal Guardian:

If you agree to be contacted for a telephone interview, please provide your details below:

**Telephone number:**	
**Mobile number:**	
**Email address:**	

1 copy for patient and parent/guardian; 1 copy for Investigator Site File; 1 copy to be kept with hospital notes

For peer review only

For peer review only - http://bmjopen.bmj.com/site/about/guidelines.xhtml

SPIRIT

STANDARD PROTOCOL ITEMS: RECOMMENDATIONS FOR INTERVENTIONAL TRIALS

SPIRIT 2013 Checklist: Recommended items to address in a clinical trial protocol and related documents*

Section/item	Item No	Description	Pg No.
**Administrative information**			
Title	1	Descriptive title identifying the study design, population, interventions, and, if applicable, trial acronym	1
Trial registration	2a	Trial identifier and registry name. If not yet registered, name of intended registry	3
	2b	All items from the World Health Organization Trial Registration Data Set	throughout
Protocol version	3	Date and version identifier	16
Funding	4	Sources and types of financial, material, and other support	20
Roles and responsibilities	5a	Names, affiliations, and roles of protocol contributors	1 & 19
	5b	Name and contact information for the trial sponsor	16 &20
	5c	Role of study sponsor and funders, if any, in study design; collection, management, analysis, and interpretation of data; writing of the report; and the decision to submit the report for publication, including whether they will have ultimate authority over any of these activities	20
	5d	Composition, roles, and responsibilities of the coordinating centre, steering committee, endpoint adjudication committee, data management team, and other individuals or groups overseeing the trial, if applicable (see Item 21a for data monitoring committee)	15
**Introduction**			
Background and rationale	6a	Description of research question and justification for undertaking the trial, including summary of relevant studies (published and unpublished) examining benefits and harms for each intervention	5
	6b	Explanation for choice of comparators	5
Objectives	7	Specific objectives or hypotheses	6

For peer review only

Trial design	8	Description of trial design including type of trial (eg, parallel group, crossover, factorial, single group), allocation ratio, and framework (eg, superiority, equivalence, noninferiority, exploratory)	6

Methods: Participants, interventions, and outcomes

Study setting	9	Description of study settings (eg, community clinic, academic hospital) and list of countries where data will be collected. Reference to where list of study sites can be obtained	6
Eligibility criteria	10	Inclusion and exclusion criteria for participants. If applicable, eligibility criteria for study centres and individuals who will perform the interventions (eg, surgeons, psychotherapists)	6&7
Interventions	11a	Interventions for each group with sufficient detail to allow replication, including how and when they will be administered	7
	11b	Criteria for discontinuing or modifying allocated interventions for a given trial participant (eg, drug dose change in response to harms, participant request, or improving/worsening disease)	8
	11c	Strategies to improve adherence to intervention protocols, and any procedures for monitoring adherence (eg, drug tablet return, laboratory tests)	10
	11d	Relevant concomitant care and interventions that are permitted or prohibited during the trial	7&8
Outcomes	12	Primary, secondary, and other outcomes, including the specific measurement variable (eg, systolic blood pressure), analysis metric (eg, change from baseline, final value, time to event), method of aggregation (eg, median, proportion), and time point for each outcome. Explanation of the clinical relevance of chosen efficacy and harm outcomes is strongly recommended	11
Participant timeline	13	Time schedule of enrolment, interventions (including any run-ins and washouts), assessments, and visits for participants. A schematic diagram is highly recommended (see Figure)	6
Sample size	14	Estimated number of participants needed to achieve study objectives and how it was determined, including clinical and statistical assumptions supporting any sample size calculations	3
Recruitment	15	Strategies for achieving adequate participant enrolment to reach target sample size	6

Methods: Assignment of interventions (for controlled trials)

Allocation:

For peer review only - http://bmjopen.bmj.com/site/about/guidelines.xhtml

Sequence generation	16a	Method of generating the allocation sequence (eg, computer-generated random numbers), and list of any factors for stratification. To reduce predictability of a random sequence, details of any planned restriction (eg, blocking) should be provided in a separate document that is unavailable to those who enrol participants or assign interventions	1&6
Allocation concealment mechanism	16b	Mechanism of implementing the allocation sequence (eg, central telephone; sequentially numbered, opaque, sealed envelopes), describing any steps to conceal the sequence until interventions are assigned	NA
Implementation	16c	Who will generate the allocation sequence, who will enrol participants, and who will assign participants to interventions	6
Blinding (masking)	17a	Who will be blinded after assignment to interventions (eg, trial participants, care providers, outcome assessors, data analysts), and how	NA
	17b	If blinded, circumstances under which unblinding is permissible, and procedure for revealing a participant's allocated intervention during the trial	NA
**Methods: Data collection, management, and analysis**			
Data collection methods	18a	Plans for assessment and collection of outcome, baseline, and other trial data, including any related processes to promote data quality (eg, duplicate measurements, training of assessors) and a description of study instruments (eg, questionnaires, laboratory tests) along with their reliability and validity, if known. Reference to where data collection forms can be found, if not in the protocol	9,11, 12, 14
	18b	Plans to promote participant retention and complete follow-up, including list of any outcome data to be collected for participants who discontinue or deviate from intervention protocols	10
Data management	19	Plans for data entry, coding, security, and storage, including any related processes to promote data quality (eg, double data entry; range checks for data values). Reference to where details of data management procedures can be found, if not in the protocol	10
Statistical methods	20a	Statistical methods for analysing primary and secondary outcomes. Reference to where other details of the statistical analysis plan can be found, if not in the protocol	12
	20b	Methods for any additional analyses (eg, subgroup and adjusted analyses)	14

	20c	Definition of analysis population relating to protocol non-adherence (eg, as randomised analysis), and any statistical methods to handle missing data (eg, multiple imputation)	13
**Methods: Monitoring**			
Data monitoring	21a	Composition of data monitoring committee (DMC); summary of its role and reporting structure; statement of whether it is independent from the sponsor and competing interests; and reference to where further details about its charter can be found, if not in the protocol. Alternatively, an explanation of why a DMC is not needed	16
	21b	Description of any interim analyses and stopping guidelines, including who will have access to these interim results and make the final decision to terminate the trial	NA
Harms	22	Plans for collecting, assessing, reporting, and managing solicited and spontaneously reported adverse events and other unintended effects of trial interventions or trial conduct	10&11
Auditing	23	Frequency and procedures for auditing trial conduct, if any, and whether the process will be independent from investigators and the sponsor	16
**Ethics and dissemination**			
Research ethics approval	24	Plans for seeking research ethics committee/institutional review board (REC/IRB) approval	15
Protocol amendments	25	Plans for communicating important protocol modifications (eg, changes to eligibility criteria, outcomes, analyses) to relevant parties (eg, investigators, REC/IRBs, trial participants, trial registries, journals, regulators)	Not provided
Consent or assent	26a	Who will obtain informed consent or assent from potential trial participants or authorised surrogates, and how (see Item 32)	9
	26b	Additional consent provisions for collection and use of participant data and biological specimens in ancillary studies, if applicable	9
Confidentiality	27	How personal information about potential and enrolled participants will be collected, shared, and maintained in order to protect confidentiality before, during, and after the trial	9 &10
Declaration of interests	28	Financial and other competing interests for principal investigators for the overall trial and each study site	20
Access to data	29	Statement of who will have access to the final trial dataset, and disclosure of contractual agreements that limit such access for investigators	16

review only

Ancillary and post-trial care	30	Provisions, if any, for ancillary and post-trial care, and for compensation to those who suffer harm from trial participation	NA
Dissemination policy	31a	Plans for investigators and sponsor to communicate trial results to participants, healthcare professionals, the public, and other relevant groups (eg, via publication, reporting in results databases, or other data sharing arrangements), including any publication restrictions	15
	31b	Authorship eligibility guidelines and any intended use of professional writers	Not provided
	31c	Plans, if any, for granting public access to the full protocol, participant-level dataset, and statistical code	Full protocol is
**Appendices**			
Informed consent materials	32	Model consent form and other related documentation given to participants and authorised surrogates	Supplementary
Biological specimens	33	Plans for collection, laboratory evaluation, and storage of biological specimens for genetic or molecular analysis in the current trial and for future use in ancillary studies, if applicable	7

*It is strongly recommended that this checklist be read in conjunction with the SPIRIT 2013 Explanation & Elaboration for important clarification on the items. Amendments to the protocol should be tracked and dated. The SPIRIT checklist is copyrighted by the SPIRIT Group under the Creative Commons "Attribution-NonCommercial-NoDerivs 3.0 Unported" license.

For peer review only

For peer review only - http://bmjopen.bmj.com/site/about/guidelines.xhtml
