# Supplementary material for: Use of selective gut decontamination in critically ill children: protocol for the Paediatric Intensive Care and Infection Control (PICnIC) pilot study
Source: BMJ Open. 2022 Mar 11;12(3):e061838. doi: 10.1136/bmjopen-2022-061838 (PMC8919465; doi:10.1136/bmjopen-2022-061838)
Supplement: Reviewer comments [file bmjopen-2022-061838.reviewer_comments.pdf]

PEER REVIEW HISTORY

BMJ Open publishes all reviews undertaken for accepted manuscripts. Reviewers are asked to complete a checklist review form (**http://bmjopen.bmj.com/site/about/resources/checklist.pdf**) and are provided with free text boxes to elaborate on their assessment. These free text comments are reproduced below.

ARTICLE DETAILS

**TITLE (PROVISIONAL)**	Use of selective gut decontamination in critically ill children: protocol for the Paediatric Intensive Care and Infection Control (PICnIC) pilot study
**AUTHORS**	Brown, Alanna; Ferrando, Paloma; Popa, Mariana; de la Fuente, Gema Milla; Pappachan, John; Cuthbertson, Brian; Drikite, Laura; Feltbower, Richard; Gouliouris, Theodore; Sale, Isobel; Shulman, Robert; Tume, Lyvonne; Myburgh, John; Woolfall, Kerry; Harrison, David; Mouncey, Paul; Rowan, Kathryn; Pathan, Nazima

This article was not externally reviewed at BMJ Open. Protocols that have been independently assessed prior to submission to BMJ Open are usually fast-tracked to publication on the grounds that further substantial changes will not be possible. This independent assessment will usually be external, independent review for both a substantial grant award from a non-commercial or government funder and ethics approval.
